# A Novel Network Pharmacology Strategy to Decode Metabolic Biomarkers and Targets Interactions for Depression

**DOI:** 10.3389/fpsyt.2020.00667

**Published:** 2020-07-15

**Authors:** Yao Gao, Teng Xu, Ying-Xia Zhao, Ting Ling-Hu, Shao-Bo Liu, Jun-Sheng Tian, Xue-Mei Qin

**Affiliations:** ^1^Modern Research Center for Traditional Chinese Medicine, Shanxi University, Taiyuan, China; ^2^Shanxi Key Laboratory of Active Constituents Research and Utilization of TCM, Taiyuan, China

**Keywords:** metabolic biomarkers, depression, network pharmacology, drug-target network, docking score-weighted multiple pharmacology index (*DSWMP*)

## Abstract

Depression is one of the most prevalent and serious mental disorders with a worldwide significant health burden. Metabolic abnormalities and disorders in patients with depression have attracted great research attention. Thirty-six metabolic biomarkers of clinical plasma metabolomics were detected by platform technologies, including gas chromatography–mass spectrometry (GC–MS), liquid chromatography–mass spectrometry (LC–MS) and proton nuclear magnetic resonance (^1^H-NMR), combined with multivariate data analysis techniques in previous work. The principal objective of this study was to provide valuable information for the pathogenesis of depression by comprehensive analysis of 36 metabolic biomarkers in the plasma of depressed patients. The relationship between biomarkers and enzymes were collected from the HMDB database. Then the metabolic biomarkers-enzymes interactions (MEI) network was performed and analyzed to identify hub metabolic biomarkers and enzymes. In addition, the docking score-weighted multiple pharmacology index (*DSWMP*) was used to assess the important pathways of hub metabolic biomarkers involved. Finally, validated these pathways by published literature. The results show that stearic acid, phytosphingosine, glycine, glutamine and phospholipids were important metabolic biomarkers. Hydrolase, transferase and acyltransferase involve the largest number of metabolic biomarkers. Nine metabolite targets (TP53, IL1B, TNF, PTEN, HLA-DRB1, MTOR, HRAS, INS and PIK3CA) of potential drug proteins for treating depression are widely involved in the nervous system, immune system and endocrine system. Seven important pathways, such as PI3K-Akt signaling pathway and mTOR signaling pathway, are closely related to the pathology mechanisms of depression. The application of important biomarkers and pathways in clinical practice may help to improve the diagnosis of depression and the evaluation of antidepressant effect, which provides important clues for the study of metabolic characteristics of depression.

## Introduction

Depression is one of the most prevalent and serious mental disorders. In recent years, the number of patients with depression has increased dramatically. In the world’s population, the lifetime prevalence of depressed patients is about 17% with a significant burden of disease (1). Previous research reports have found that in the United States, more than 19 million adults suffer from depression in the USA and spend more than 30 billion annually, directly or indirectly (2). In addition, the incidence of depression is about 3 to 5% and currently 26 million Chinese people suffer from depression (3). Depression is a major cause of neuropsychiatric disability worldwide and the accurate diagnosis of depression before treatment is a hub change (4). Increased studies have reported the serotonin and norepinephrine dysfunction in the central nervous system of patients with depression (5). In addition, studies have also reported that hypothalamic pituitary adrenal (HPA) axis is one of the largest neuroendocrine findings of depression (6). Abnormal changes of inflammatory cytokines and endogenous metabolites are also involved in the molecular mechanism of pathology of depression (7, 8). The occurrence and development of depression is a complicated process, the etiology and pathogenesis mechanism of depression represent challenging issues in scientific and medical research.

A new psychological immune neuroendocrine (PINE) network model on depression has been proposed to provide an in-depth understanding of the pathogenesis of depression and the treatment of the disease with antidepressant drugs (1). The PINE network model is composed of three parts of the central nervous system, immune, and endocrine molecular networks, and the three networks are interconnected (9). The three molecular networks consist of many nodes and edges. The nodes in the network can be small molecules with different properties, such as genes, proteins or metabolites. If different nodes are related in biological function, it can be connected by edges. How to determine the key underlying molecular mechanism from PINE network that plays leading roles in the depression is a difficult problem due to the high complexity of the network composition and the incompletely understanding the complex multi-targets mechanism of depression.

Network pharmacology is constructed by integrating pharmacological data and network analysis methods to provide a comprehensive approach to explain the disease pathogenesis and drug treatment mechanism (10). The network pharmacology technology is considered to be one of the next frontiers of new drug research (11). Recently, network pharmacology has been widely used in the pathogenesis of complex diseases and the mechanism of drug action. For example, Huang decoded the mechanism of traditional Chinese medicine in treating depression by analyzing drug target networks and disease target networks (12).

Increased studies have reported metabolic disorders or abnormal metabolic pathways in the plasma of depressed patients (13, 14). Thirty-six metabolic biomarkers of clinical plasma metabolomics were detected by platform technologies, including gas chromatography–mass spectrometry (GC–MS), liquid chromatography–mass spectrometry (LC–MS) and proton nuclear magnetic resonance (^1^H-NMR), combined with multivariate data analysis techniques in previous our work (13, 14). These metabolic biomarkers are distributed in disordered metabolic pathways and maybe potential diagnostic biomarkers or therapeutic markers. Therefore, how to use the network pharmacology method to mine the existing markers is of great significance to the study of the metabolic mechanism of depression.

The main purposes of this study were to comprehensively analyze plasma metabolites by network pharmacology method and provide valuable information for the pathogenesis of depression. During this process, the relationship between biomarkers and enzymes were collected from the HMDB database and the metabolic biomarkers–enzymes interactions (MEI) network was performed and analyzed to identify hub metabolic biomarkers and enzymes. In addition, the interactions between each MB and each target of the nervous system, immune system and endocrine system was calculated by systemsDock, and then the docking score-weighted multiple pharmacology index (*DSWMP*) was used to assess the importance pathways of hub metabolic biomarkers involved. Finally, the important pathways were verified through published literature. The application of important biomarkers and pathways in clinical practice may help to improve the diagnosis of depression and the evaluation of antidepressant effect, which provides important clues for the study of metabolic characteristics of depression.

## Methods

### Metabolites Data Collection and Processing

For the research object, only the metabolic biomarkers of clinical plasma metabolomics in our consideration, because the excavation of metabolites is more meaningful in the same clinical sample. Candidate metabolites refer to the statistical significance found in the original study. Unique metabolites were obtained by removing duplicates. We obtained biological function information and structural data of identified metabolic biomarkers from the Human Metabolome Database (HMDB) (15). The structures of these compounds were downloaded from PubChem (16). Enzymes related to the metabolic biomarkers were summarized from HMDB, while the functional categories of the relevant proteins were found from the UniProt database (17). The target proteins related to the nervous system (**Table S1**), immune system (**Table S2**) and endocrine system (**Table S3**) were retrieved from several Published online databases. The FDA-approved drugs for the nervous system, immune system and endocrine system with their targets were collected from DrugBank (18). The depression-related proteins were retrieved from Genecards (19), OMIM (20) and TTD (21). Finally, we carefully checked the target proteins in the literature. Integrate all target proteins and divide them into three categories, including the nervous system, immunity and endocrine.

### Molecular Docking

Molecular docking was used to investigate the affinity between metabolic biomarkers and targets. The protein 3D structures were achieved from the RCSB protein data bank (http://www.rcsb.org) (22). We chose structures with more complete peptide chains, higher resolution, and better ligands as the selection criteria for the most appropriate protein structure.

SystemsDock (http://systemsdock.unit.oist.jp/) (23) was used for network pharmacology prediction and analysis, including four specific steps, selecting proteins by different parameters, defining binding sites by interactive molecular visualizer, preparing metabolic biomarkers for the test, and performing docking simulation and evaluating the result. The docking score calculated by systemsDock was used to assess the interactions between the metabolic biomarkers and the target proteins.

### Mapping Metabolic Biomarkers–Target/Enzyme Network

Metabolic biomarker–enzyme relationships were collected from HMDB and visualized by using Cytoscape 3.4.0 (24). The network topological properties of nodes (metabolic biomarkers and enzymes) were calculated by the NetworkAnalyzer Cytoscape plugin (25). In an undirected network, the degree centrality of a node denotes the number of edges connected to this node is commonly used to measure the importance of a node in a single-layer network (26, 27). Degree centrality is a key indicator in analyzing the network, thereby reflecting the importance and influence of a metabolome biomarker or an enzyme in the metabolic biomarkers–enzyme interactions (MEI) network.

The threshold of the docking score was set to 5.52 (p*K*_d_), which was equal to the dissociation constant (*K*_d_) of 3 μM. A docking score greater than the threshold was considered to be a very good correlation between the metabolic biomarker and the target protein (23). Based on previously reported works and experimental findings, a high accuracy level (80–83%) was found to evaluate the binding of a metabolomic biomarker and a protein, when the threshold score was in the range of 4.82 to 6.11 (p*K*_d_). The metabolic biomarkers-target interactions (MTI) network was visualized by Cytoscape software.

### Docking Score-Weighted Multiple Pharmacology Index

The target-related pathways were collected from Kyoto Encyclopedia of Genes and Genomes (KEGG) (28). One metabolic biomarker can be combined with many targets, and one target can be enriched to function in many different biological pathways. Therefore, a metabolic biomarker involves multiple different biological pathways. At present, there is no method to directly confirm that the metabolic biomarkers are directly enriched in the pathway through the targets, so we have defined a docking score-weighted multiple pharmacology index (*DSWMP*):

(1), $$DSWMP\;(P_{k})=\frac{\Sigma_{i}^{N}\Sigma_{j}^{M}DS_{BiTj}}{Count\;(B_{i})},\;T_{j}\;\in \;TP_{k}$$

where *DS_BiTj_* is the affinity between metabolic biomarker *B_i_* and target *T_j_*, and *TP_k_* is a group of targets involved in pathway *P_k_*. N and M are the numbers of metabolic biomarkers and targets, respectively.

### Experimental Evidence of Important Pathways

To find experimental evidence for the predicted important pathways of depression by *DSWMP* calculation, the published literature will be identified by searching Pubmed, Embase, Cochrane Central Register of Controlled Trials and Web of Science. In the end, we chose the literature to verify the important pathways we found and provide references for the next step of research.

## Results and Discussion

**Figure 1:**
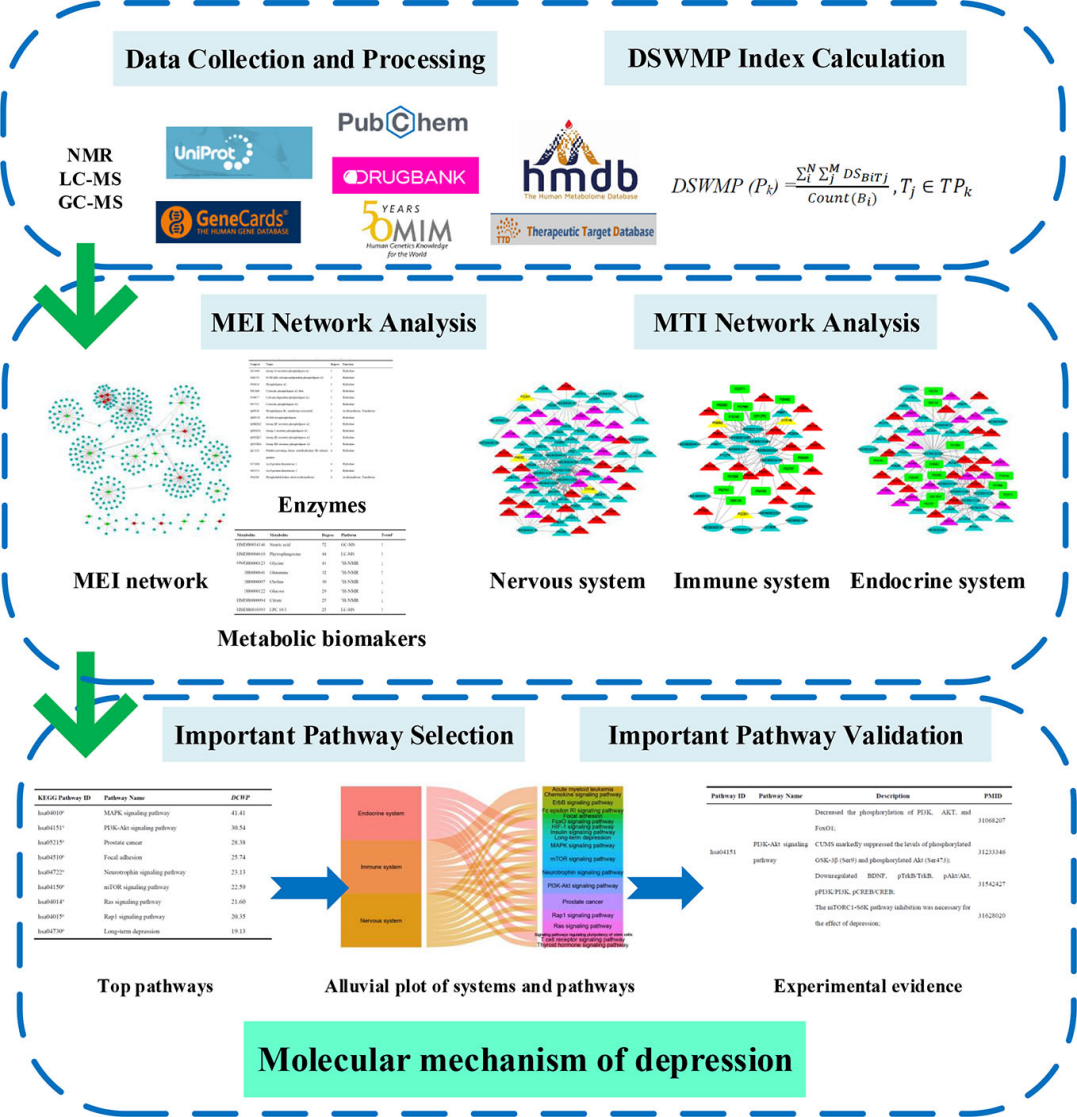
The flowchart of network pharmacology approach.

In this report, a novel network pharmacology strategy was designed to detect the important pathway and elucidate the molecular mechanisms of depression (**Figure 1**). Firstly, the relationship between biomarkers and enzymes were collected from the HMDB database and the MEI network was performed and analyzed to identify hub metabolic biomarkers and enzymes. Secondly, the interactions between each MB and each target were calculated by systemsDock, and then the *DSWMP* was used to assess the important pathways of hub metabolic biomarkers involved. Thirdly, the important pathways verified through published literature.

### Metabolic Biomarker–Enzyme Interactions (MEI) Network Analysis

The information of 36 metabonomic biomarkers and 350 enzymes are shown in **Table S4**. Most enzymes related to metabolic biomarkers belong to hydrolases, transferases and acyltransferases, which is consistent with our previous results. These enzymes mainly regulate metabolites such as energy metabolism, amino acid metabolism, gut microbe metabolism, glycerophospholipid metabolism and sphingolipid metabolism (13, 14). These enzymes play an important role in the regulation of metabolic pathways, which affect the molecular mechanism of depression. Overall, the results show that functional studies of enzymes related to metabolic biomarkers can reflect the molecular mechanism of depression, which also provides a reliable reference for the next network analysis.

**Figure 2:**
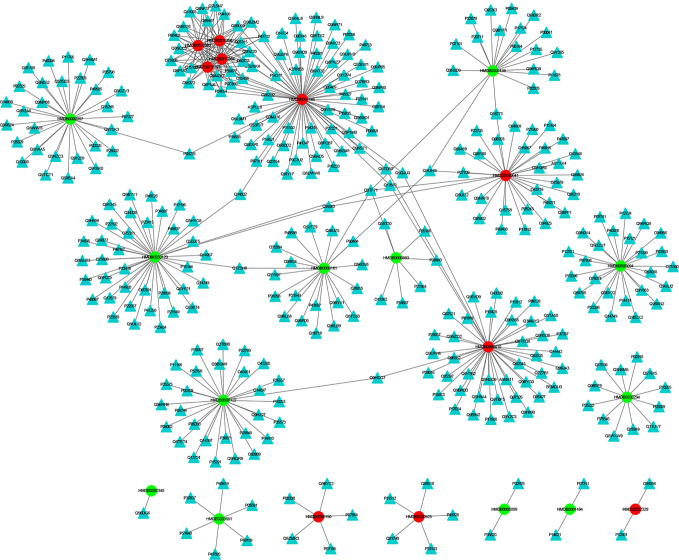
The MEI network. The cycle and triangle represent metabolic biomarker and enzyme, respectively. The color of cycle is green or red if the concentration of the metabonomic biomarker is down-regulated or up-regulated, respectively.

The MEI network was used to characterize the relationship between enzymes and metabolic biomarkers, as shown in **Figure 2**. In MEI network, the node represents a metabolic biomarker or an enzyme, and the edges represent the interactions between them originated from HMDB. It turns out that the concentrations of some biomarkers in depression patients are up-regulated, while others are down-regulated. Among these regulated biomarkers, their related enzymes form a particularly large cluster around them, suggesting that multiple enzymes regulate the same metabolite. **Tables 1** and **2** show the important enzymes and biomarkers identified by the network pharmacology approach, respectively. Among these data, previous literatures reported that cytosolic phospholipase A2 (cPLA2) is an important enzyme for PUFA metabolism and PGE 2 synthesis, which is the pivotal to the mode action of mood stabilizers in animal studies (29, 30). In addition, in peripheral blood cells of patients with depression, the mRNA expression of the gene encoding COX-2 increases sharply, which plays an important role in the pathogenesis of depression (31).

**Table 1:** Important enzymes identified by network topology analysis of MEI. network.

Uniprot	Name	Degree	Function
O15496	Group 10 secretory phospholipase A2	5	Hydrolase
O60733	85/88 kDa calcium-independent phospholipase A2	5	Hydrolase
P04054	Phospholipase A2	5	Hydrolase
P0C869	Cytosolic phospholipase A2 beta	5	Hydrolase
P39877	Calcium-dependent phospholipase A2	5	Hydrolase
P47712	Cytosolic phospholipase A2	5	Hydrolase
Q6P1J6	Phospholipase B1, membrane-associated	5	Acyltransferase, Transferase
Q86U10	60 kDa lysophospholipase	5	Hydrolase
Q9BZM2	Group IIF secretory phospholipase A2	5	Hydrolase
Q9NZ20	Group 3 secretory phospholipase A2	5	Hydrolase
Q9NZK7	Group IIE secretory phospholipase A2	5	Hydrolase
Q9UNK4	Group IID secretory phospholipase A2	5	Hydrolase
Q15102	Platelet-activating factor acetylhydrolase IB subunit gamma	4	Hydrolase
O75608	Acyl-protein thioesterase 1	4	Hydrolase
O95372	Acyl-protein thioesterase 2	4	Hydrolase
P04180	Phosphatidylcholine-sterol acyltransferase	4	Acyltransferase, Transferase
P68402	Platelet-activating factor acetylhydrolase IB subunit beta	4	Hydrolase
Q13093	Platelet-activating factor acetylhydrolase	4	Hydrolase
Q6P1A2	Lysophospholipid acyltransferase 5	4	Acyltransferase, Transferase
Q7L5N7	Lysophosphatidylcholine acyltransferase 2	4	Acyltransferase, Transferase
Q8NCC3	Group XV phospholipase A2	4	Acyltransferase, Hydrolase, Transferase
Q8NF37	Lysophosphatidylcholine acyltransferase 1	4	Acyltransferase, Transferase
Q99487	Platelet-activating factor acetylhydrolase 2, cytoplasmic	4	Hydrolase
Q14032	Bile acid-CoA:amino acid N-acyltransferase	2	Acyltransferase, Hydrolase, Serine esterase, Transferase
P06276	Cholinesterase	2	Hydrolase, Serine esterase
Q13510	Acid ceramidase	2	Hydrolase
Q16773	Kynurenine–oxoglutarate transaminase 1	2	Aminotransferase, Lyase, Transferase
Q5QJU3	Alkaline ceramidase 2	2	Hydrolase
Q8TDN7	Alkaline ceramidase 1	2	Hydrolase
Q969I3	Glycine N-acyltransferase-like protein 1	2	Acyltransferase, Transferase
Q9HCG7	Non-lysosomal glucosylceramidase	2	Glycosidase, Hydrolase

**Table 2:** Important metabolic biomarkers identified by network topology analysis of MEI network.

Metabolite	Metabolite	Degree	Platform	Trend^a^
HMDB0034146	Stearic acid	72	GC–MS	↑
HMDB0004610	Phytosphingosine	44	LC–MS	↑
HMDB0000123	Glycine	41	^1^H-NMR	↓
HMDB0000641	Glutamine	32	^1^H-NMR	↑
HMDB0000097	Choline	30	^1^H-NMR	↓
HMDB0000122	Glucose	29	^1^H-NMR	↓
HMDB0000094	Citrate	25	^1^H-NMR	↓
HMDB0010393	LPC 10:3	25	LC–MS	↑
HMDB0010383	LPC 16:1	25	LC–MS	↑
HMDB0010396	LPC 21:4	25	LC–MS	↑
HMDB0010384	LPC 18:0	25	LC–MS	↑
HMDB0000161	Alanine	21	^1^H-NMR	↓
HMDB0000159	Phenylalanine	19	^1^H-NMR	↓
HMDB0000294	Urea	12	GC–MS	↓
HMDB0000883	Valine	7	^1^H-NMR/GC–MS	↓
HMDB0003681	4-Acetamidobutanoic acid	6	LC–MS	↓
HMDB0000190	Lactate	5	^1^H-NMR	↑
HMDB0000925	TMAO	5	^1^H-NMR	↑
HMDB0002329	Oxalic acid	2	GC–MS	↑
HMDB0000099	L-Cystathionine	2	LC–MS	↓
HMDB0001494	Acetylphosphate	2	LC–MS	↓
HMDB0060348	4-oxohex-2-enedioic acid	1	LC–MS	↓

^a^:The fold change was calculated as the ratio of the concentration of metabolite of the depression to that of healthy control.

Among these metabolite-related enzymes, the phospholipase A2 family is related to many metabolites, so it is also important to study the function of depression. Previous research reports suggest that different phospholipase A2 types are associated with somatic symptoms of depression (32). It is believed that genetic variation of the phospholipase A2 gene increases the risk factor of depression induced by IFN-α (32). In addition, phospholipase A2 inhibitors have potential therapeutic effects in treating inflammation-related diseases, such as depression (33). Moreover, eicosapentaenoic acid (EPA) could up-regulate the expression of cytosolic phospholipase A2 gene and play an antidepressant effect in clinic, which shows the superiority of EPA antidepressant effect (34). Another research group also found that the same G allele of the PLA2 BanI polymorphism is one of the risk factors for depression in Korean populations (35). The expression levels of acyl protein thioesterase 2 (APT-2) and oleamide are increased in chronic mild stress (CMS). This provides a reference for the treatment of depression by targeting these proteins (36). At present, there is no literature on the relationship between acetylhydrolase and depression, which may be a novel research point.

Fourteen metabolic biomarkers are associated with more than ten enzymes in MEI network (**Table 2**). The concentrations of most MB were down-regulated, while stearic acid, phytosphingosine, glutamine and phospholipids were up-regulated. It is well known that fatty acids are an important source of human body production and storage. Acetyl-coenzyme A (CoA) produced by oxidation of β-fatty acids can produce adenosine triphosphate (ATP) in the TCA cycle and could be converted to ketone bodies for storage in the kidney and liver (37). There are reports showing that the concentration of stearic acid in plasma of patients with depression is significantly increased, which may lead to blockage of fatty acid transport and inhibition of TCA cycle, which is consistent with the previous research results (38, 39). Moreover, the AUCs for oxalic acid and stearic acid were >0.7, indicating a great clinical diagnostic value. Therefore, stearic acid may be a diagnostic indicator of depression. These results indicate that the metabolic biomarkers in MEI network are involved in the molecular mechanism of depression.

In addition, most metabolic biomarkers belong to long-chain fatty acids and phytosphingosine, which indicates that the lipid metabolism disorder plays a crucial role in the mechanism of depression. These metabolic biomarkers are involved in glycerophospholipid metabolism and sphingolipid metabolism. Among them, sphingosine involves various biological processes, including cell–cell interactions, cell proliferation, differentiation and apoptosis. Studies have reported that elevated levels of hemolytic phosphatidylcholine also increase oxidative stress, which is a key factor in the onset of depression (40). The results indicate that the long-chain fatty acids and phytosphingosine in MEI network is involved in the pathological mechanism of depression.

Glutamine and its related enzymes form an independent part of the MEI network, suggesting that glutamine may play an unusual role in the pathogenesis mechanism of depression. Glutamate is associated with the neurobiology of depression and can cause neurotoxicity if over-released (40). In addition, glutamine and glutamate can be converted between neurons and astrocytes, which is necessary for the steady state of the glutamine-glutamic acid cycle (41). Therefore, the increase in glutamine in the plasma of depression patients may be a compensatory adaptation to glutamate-induced neurotoxicity. The results show that the glutamine in the MEI network is involved in the pathological mechanism of depression.

### Metabolic Biomarker–Target Interactions (MTI) Network Analysis

**Figure 3:**
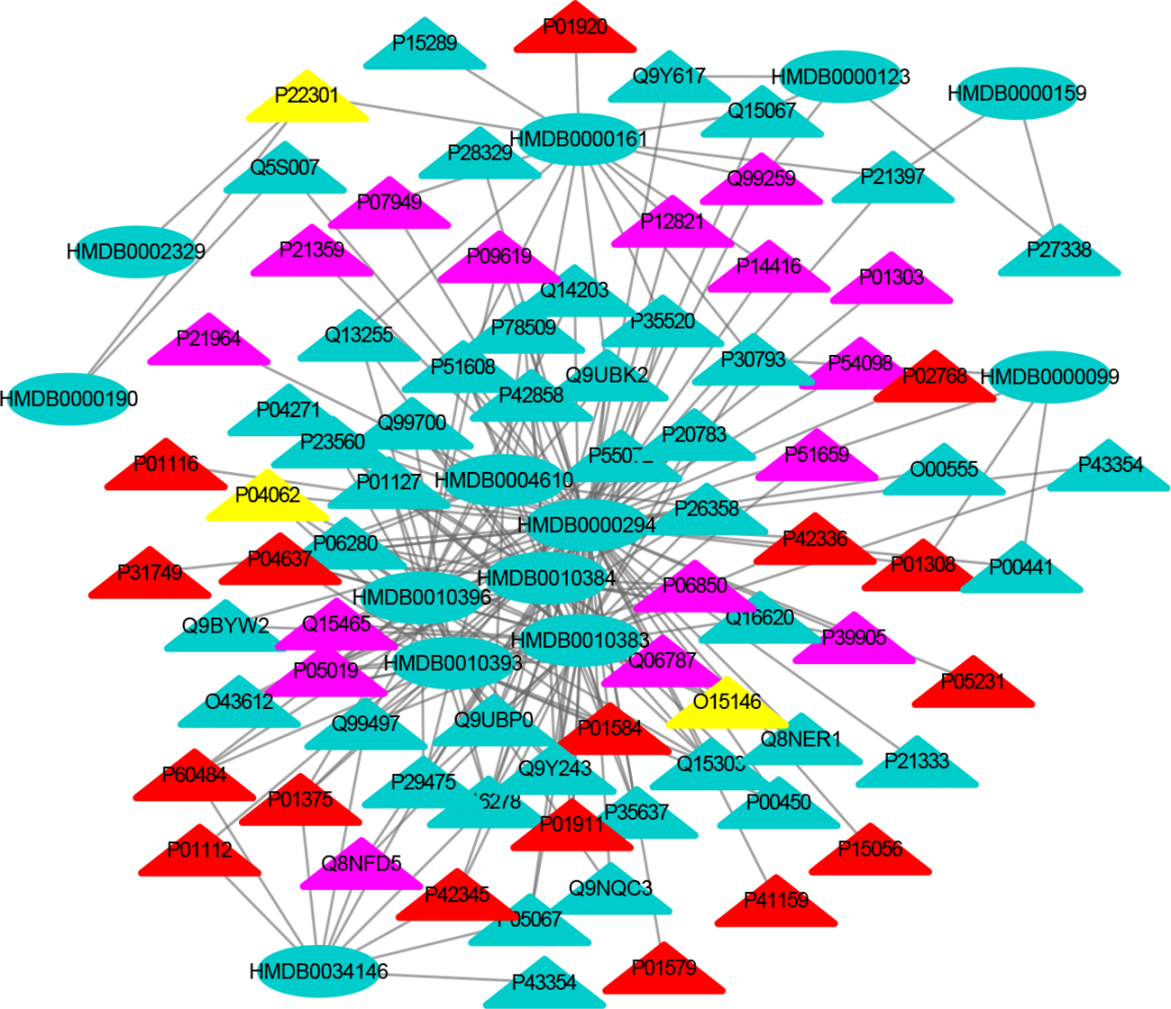
The MTI network of the nervous system. The ellipses refer to metabolic biomarkers, and triangles refer to targets, respectively. The color of the nodes indicates that the three systems connected to depression are different, including nervous system, immune system and endocrine system. The colors of the nodes are blue, yellow, and red, indicating that the target is connected to one system, two systems, and three systems, respectively.

**Figure 4:**
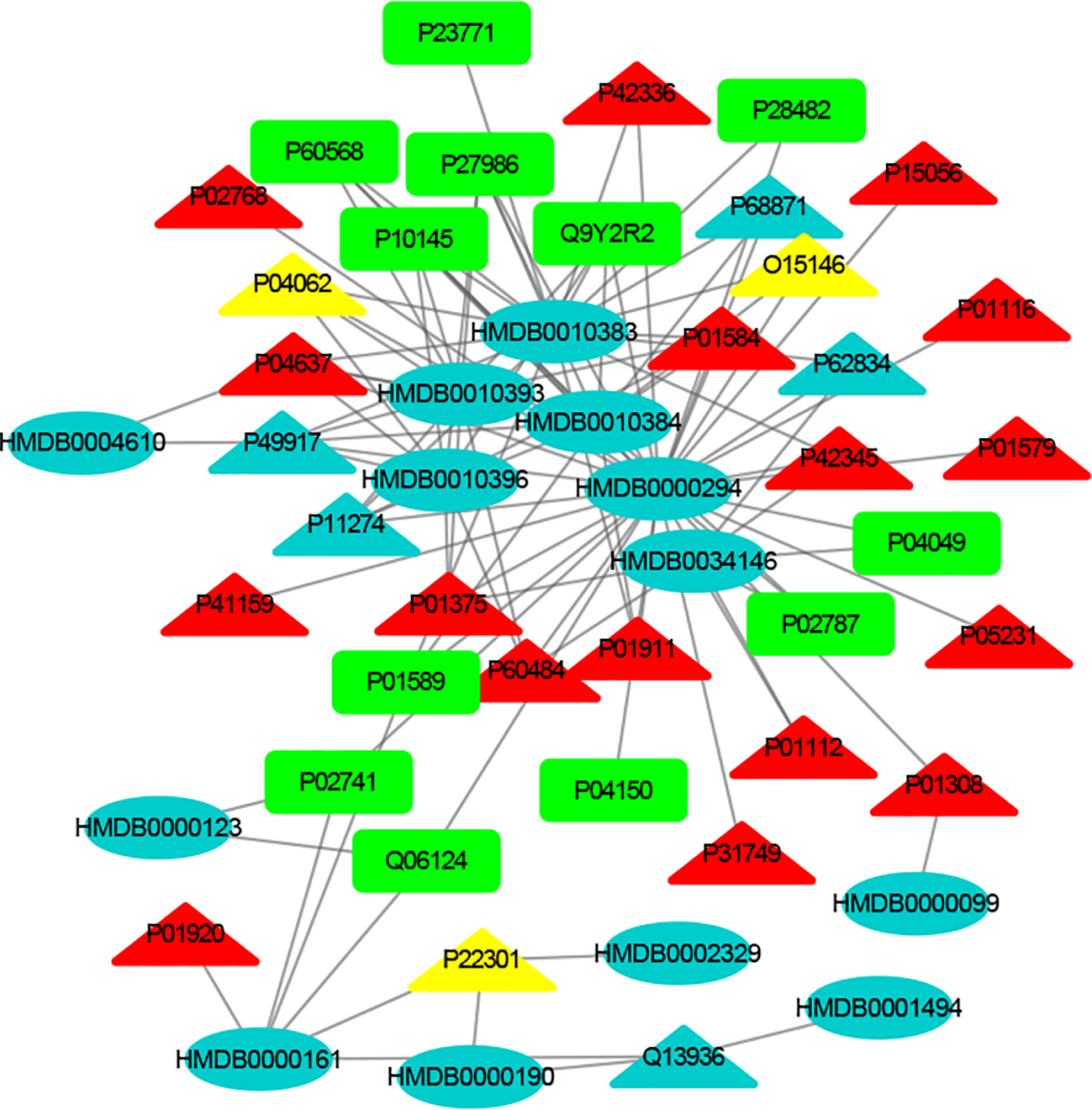
The MTI network of the immune system. The ellipses refer to metabolic biomarkers, and triangles refer to targets, respectively. The color of the nodes indicates that the three systems connected to depression are different, including nervous system, immune system and endocrine system. The colors of the nodes are blue, yellow, and red, indicating that the target is connected to one system, two systems, and three systems, respectively.

**Figure 5:**
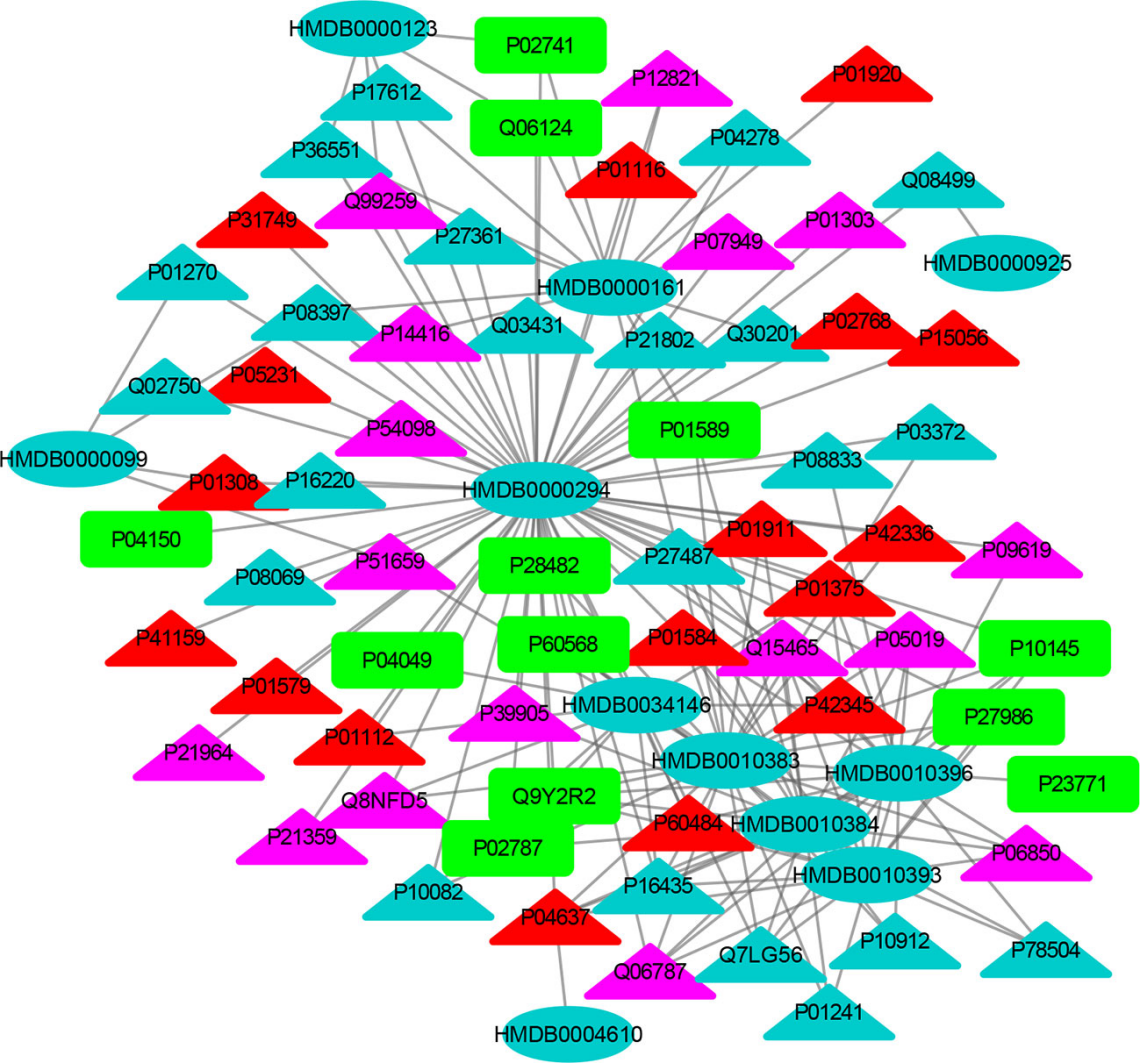
The MTI network of the endocrine system. The ellipses refer to metabolic biomarkers, and triangles refer to targets, respectively. The color of the nodes indicates that the three systems connected to depression are different, including nervous system, immune system and endocrine system. The colors of the nodes are blue, yellow, and red, indicating that the target is connected to one system, two systems, and three systems, respectively.

**Tables S1–S3** show the nervous system, immune system and endocrine system target proteins, and the nervous system, immune system and endocrine system target numbers are 155, 60 and 125, respectively. The interactions between each metabolic biomarker and the target protein were performed by molecular docking. The MTI network of nervous system, immune system and endocrine system were visualized by Cytoscape, respectively (**Figures 3**–**5**). In MTI network, the node represents a metabolic biomarker or a target, and the edges represent the interactions between them from the docking score meeting the set threshold. The metabolic biomarker links the target to a highly interconnected cluster. In order to analyze the correlation between metabolic biomarkers and targets and to mine important molecules, we calculated the topological parameter of the network, namely degree centrality.

The MTI network of the nervous system (**Figure 3**) contains 13 metabolic biomarkers and 78 targets, resulting in 232 edges associations between them. The average number of targets for each metabolic biomarker is 17.85 and the average number of metabolic biomarkers for each target is 2.97. Similarly, the MTI network of the immune system (**Figure 4**) contains 13 metabolic biomarkers and 37 targets, resulting in 107 edges associations between them. The average number of targets for each metabolic biomarker is 8.23 and the average number of metabolic biomarkers for each target is 2.89. Moreover, the MTI network of the endocrine system (**Figure 5**) contains 11 metabolic biomarkers and 67 targets, resulting in 78 edges associations between them. The average number of targets for each metabolic biomarker is 15.91 and the average number of metabolic biomarkers for each target is 2.61. This indicates that the molecular mechanism of depression in the nervous system, immune system and endocrine system has the characteristics of multiple metabolic biomarkers and multiple targets.

In these three MTI networks, the three metabolic biomarkers of urea, LPC 16:1 and LPC 18:0 have a higher degree centrality in the network. The number of targets that the three metabolic biomarkers could bind to in the depression-related nervous system, immune system and endocrine system are 68, 33, 61; 40, 18, 25; 29, 13, 20, respectively. The role of these metabolic biomarkers in the pathogenesis of depression is worthy of our in-depth study, because these metabolic biomarkers play a crucial role in the three networks. Among these metabolic biomarkers, urea, LPC 16:1 and LPC 18:0 can be classified as organic carbonic acids or glycerophospholipids, and further research is needed on their role in the pathogenesis of depression.

There are nine identical targets in these three systems, which simultaneously play an important role in the three MTI networks involved in the pathogenesis of depression. Among these targets related to metabolic biomarkers, P04637 (Tumor Protein P53), P01584 (Interleukin 1 Beta), P01375 (Tumor Necrosis Factor), P60484 (Phosphatase And Tensin Homolog), P01911 (Major Histocompatibility Complex, Class II, DR Beta 1), P42345 (Mechanistic Target of Rapamycin Kinase), P01112 (HRas Proto-Oncogene, GTPase), P01308 (Insulin), P42336 (Phosphatidylinositol-4,5-Bisphosphate 3-Kinase Catalytic Subunit Alpha), were widely involved in the nervous system, immune system and endocrine system.

The minor allele 72C of the tumor protein P53 (TP53) gene plays a protective role in the occurrence of depression. It participates in the pathological mechanism of depression through the cell survival and death regulation (42). Previous studies have reported that proinflammatory cytokines such as IL-1 beta (IL1B) (43) and tumor necrosis factor (TNF) (44) have been implicated in the pathogenesis molecular mechanism of depression. The evidence provided by Liu suggests that the rs701848, rs2735343 and rs112025902 polymorphisms in the phosphatase and tensin homologous gene (PTEN) genes may be related to the risk of depression in Chinese (45). Major Histocompatibility Complex (HLA-DRB1) plays a significant role in the immune system by presenting peptides derived from extracellular proteins (46). HRas Proto-Oncogene (HRAS) (46) can encode some genes in signal transduction pathways. These proteins can be linked to GTP and GDP, and they have their own GTPase activity. In addition, rapamycin kinase (mTOR) is a serine/threonine kinase that regulates cell proliferation (47). Li research found that the activation of mTOR in the prefrontal cortex of rats was one of the important mechanisms by which ketamine exerts antidepressant effects (48). Some related studies have also found that acute ketamine administration will activate mTOR in the peripheral blood of patients with depression (49). The antioxidant alpha lipoic acid has been shown to increase insulin (INS) sensitivity and has been used to treat diabetic patients. INS also plays an important role in the pathogenesis of depression. Therefore, the nutrient alpha lipoic acid should be clinically tested as an adjunct treatment for depression. Therefore, some scholars suggested that the nutrient alpha lipoic acid should be used as an adjunct treatment for depression (50). Phosphatidylinositol 4, 5-bisphosphate 3-kinase catalytic subunit alpha isoform (PIK3CA) inhibitors used in bipolar disease and depression (51). Consequently, these results demonstrated that the crucial roles of nine identical targets in the treatment of depression and further confirmed that drug works in a multi-targets manner to treat depression.

### Important Pathways Selection and Validation

In order to improve the therapeutic effect while reducing side effects, drug treatment of diseases usually go through a variety of target methods (52). These targets involve multiple different pathways of disease pathology. The target-pathway approach is a disease-specific research module, which has been widely used in disease pathology exploration and drug development. To find the important pathological mechanism of depression, the strategy of *DSWMP* index was applied and calculated to select crucial pathway, which provides a methodological reference for the research and development of disease. The *DSWMP* index is an indicator to evaluate the importance of the pathway. The size of the DSWMP index is determined by the number of metabolic biomarkers binding targets and the binding energy between them. In other words, the DSWMP index method can be used to evaluate the importance of metabolic biomarkers in pathways.

**Figure 6:**
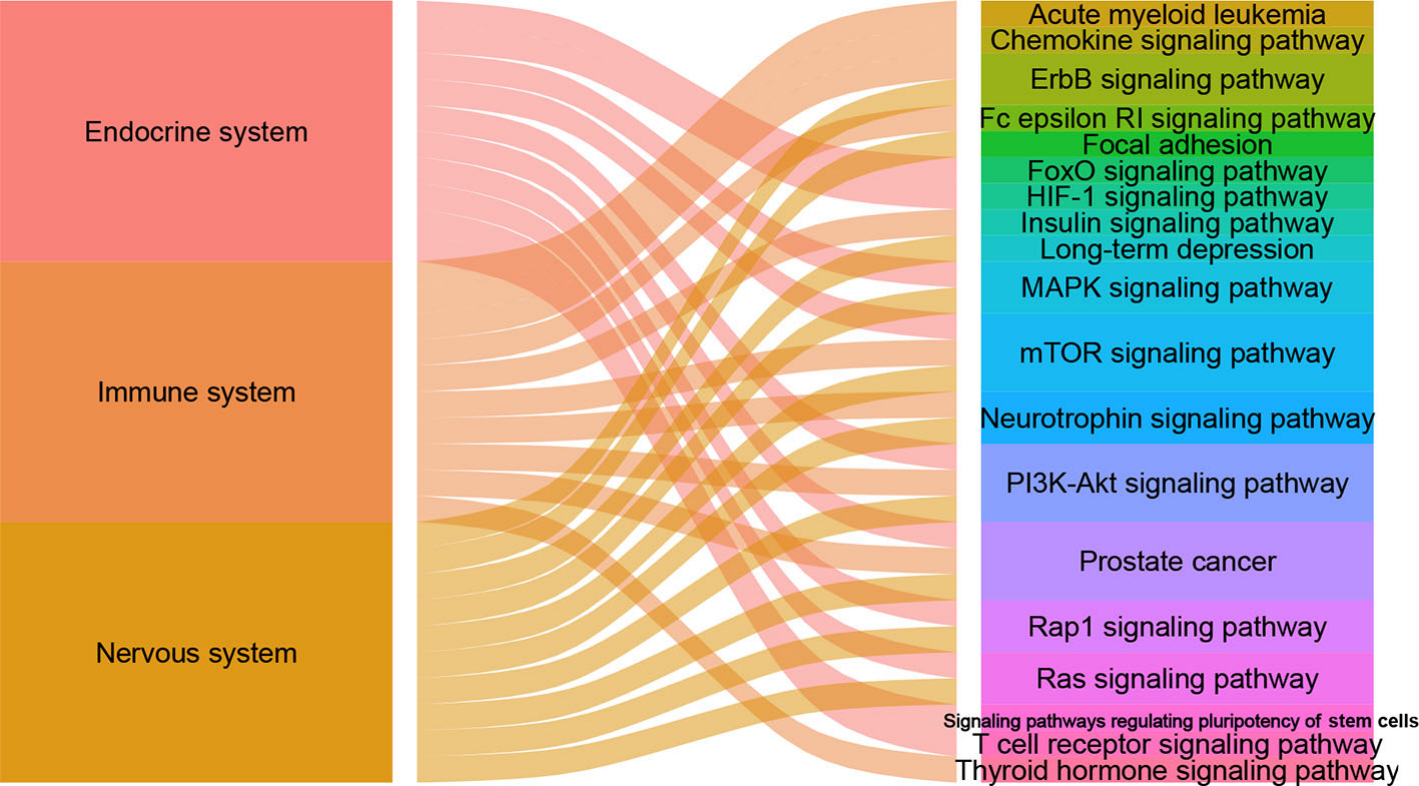
The alluvial plot of systems and pathways. The left column represents nervous system, immune system and endocrine system, the right represents pathways, and the edge represents the relationship between them. A larger edge width indicates the number of pathways-linked systems.

There are 105, 113 and 125 metabolic biomarkers-related targets involved in the nervous system pathways immune system and endocrine system of depression, respectively. In each function, the top 10 pathways of *DSWMP* index are shown in **Table 3** and **Figure 6**. Among these pathways, the pathways of hsa04151 (PI3K-Akt signaling pathway) and hsa04150 (mTOR signaling pathway) are top-ranked in the pathological mechanism of depression among the nervous system, immune system and endocrine system, indicating that these two pathways play an important role in the pathogenesis of depression. The hsa04151 has been reported involved in the inhibition of apoptosis, cell proliferation and expression of inflammatory cytokines (53, 54). The hsa04150 pathway connects intracellular and extracellular signaling communication, and plays an important role in the protein synthesis process of new synaptic connections (55). Recent research supports the hypothesis that major depression may be the result of disruption of mTOR-dependent translational regulation (13, 14). This result indicates that this pathway plays a crucial role in the molecular mechanism of depression. In addition, there are another five pathways, including hsa04010 (MAPK signaling pathway), hsa04012 (ErbB signaling pathway), hsa04722 (Neurotrophin signaling pathway), hsa04015 (Rap1 signaling pathway) and hsa04014 (Ras signaling pathway), were involved in two of the nervous system, immune system and endocrine system related to depression. Finally, the experimental evidence for the seven important signaling pathways with the most significant therapeutic relationships of depression is shown in **Table 4**. These pathways could have closely related to the pathological process of depression and need to research in depth.

**Table 3:** Top ten pathways for the nervous system, immune system and endocrine system.

KEGG Pathway ID	Pathway Name	*DCWP*
hsa04010^a^	MAPK signaling pathway	41.41
hsa04151^a^	PI3K-Akt signaling pathway	30.54
hsa05215^a^	Prostate cancer	28.38
hsa04510^a^	Focal adhesion	25.74
hsa04722^a^	Neurotrophin signaling pathway	23.13
hsa04150^a^	mTOR signaling pathway	22.59
hsa04014^a^	Ras signaling pathway	21.60
hsa04015^a^	Rap1 signaling pathway	20.35
hsa04730^a^	Long-term depression	19.13
hsa04012^a^	ErbB signaling pathway	18.50
hsa04151^b^	PI3K-Akt signaling pathway	25.21
hsa04062^b^	Chemokine signaling pathway	23.30
hsa05215^b^	Prostate cancer	22.58
hsa04150^b^	mTOR signaling pathway	22.29
hsa04919^b^	Thyroid hormone signaling pathway	19.97
hsa05221^b^	Acute myeloid leukemia	18.45
hsa04012^b^	ErbB signaling pathway	18.45
hsa04664^b^	Fc epsilon RI signaling pathway	18.41
hsa04722^b^	Neurotrophin signaling pathway	18.10
hsa04910^b^	Insulin signaling pathway	17.49
hsa04151^c^	PI3K-Akt signaling pathway	37.33
hsa04150^c^	mTOR signaling pathway	25.73
hsa04068^c^	FoxO signaling pathway	25.71
hsa05215^c^	Prostate cancer	25.11
hsa04660^c^	T cell receptor signaling pathway	25.07
hsa04066^c^	HIF-1 signaling pathway	23.17
hsa04015^c^	Rap1 signaling pathway	23.16
hsa04014^c^	Ras signaling pathway	23.14
hsa04010^c^	MAPK signaling pathway	21.95
hsa04550^c^	Signaling pathways regulating pluripotency of stem cells	20.65

^a^,^b^, and ^c^: the pathway was related to target of nervous system, immune system and endocrine system, respectively.

**Table 4:** Experimental evidence for the seven important signaling pathways with most significant therapeutic relationships of depression.

Pathway ID	Pathway Name	Description	PMID
hsa04151	PI3K-Akt signaling pathway	Decreased the phosphorylation of PI3K, AKT, and FoxO1;	31068207
		CUMS markedly suppressed the levels of phosphorylated GSK-3β (Ser9) and phosphorylated Akt (Ser473);	31233346
		Downregulated BDNF, pTrkB/TrkB, pAkt/Akt, pPI3K/PI3K, pCREB/CREB;	31542427
hsa04150	mTOR signaling pathway	The mTORC1-S6K pathway inhibition was necessary for the effect of depression;	31628020
		Reduction in the levels of mature BDNF and mTOR (Ser2448) phosphorylation in the hippocampus;	31078612
		UCMS-induced reductions of p70S6K and post-synaptic density 95 (PSD-95) mRNA levels, and of phospho-mTOR and phospho-4EBP1 in the prefrontal cortex, hippocampus, hypothalamus, and olfactory bulb;	27374162
hsa04010	MAPK signaling pathway	Downregulated expression of the mitogen activated protein kinase (MAPK) phosphatase 1 (MKP-1) and the downregulated phosphorylation of extracellular signal-regulated kinase (pERK) in the anterior cingulate cortex (ACC) of mice;	32109506
		CTRP3 may be an innovative therapeutic target for treating patients with depression through regulating p38 and JNK signaling;	31629950
		The MAPK/ERK signaling pathway has been shown to be involved in the pathogenesis of MDD and the rapid onset of action of antidepressant therapies;	30859414
hsa04012	ErbB signaling pathway	The stressed rats showed elevated expression of NRG1 and phosphorylated ErbB4 (pErbB4) in the myocardium, whereas ErbB2 and pErbB2 were inhibited;	27133902
		The stressed rats displayed elevated expression of NRG1 and phosphorylated ErbB4 (pErbB4) in the prefrontal cortex, whereas ErbB2 and pErbB2 were inhibited;	26626816
hsa04722	Neurotrophin signaling pathway	Decreased neurotrophic factors expression and neurotrophin receptors signaling have repeatedly been reported in association with stress, depression, and neurodegenerative disorders;	28315978
		p75 neurotrophin receptor/nerve growth factor signaling and innate immune toll-like receptor signaling in MDD;	31889537
		Reduced synaptic markers in hippocampus, demonstrated by reductions in β III-tubulin (neuronal marker), PSD-95, SNAP-25, and neurotrophin-3;	28451885
hsa04015	Rap1 signaling pathway	Rap1-MKK3/6-p38 MAPK pathway in the induction of mGluR-dependent long term depression (LTD) by directly coupling to receptor trafficking machineries to facilitate the loss of synaptic AMPA receptors;	14709549
		The small G-protein Rap and the transcription factor STAT-3 are also involved since reducing the levels of Rap1 (using small interfering RNA) or STAT-3 (using dominant negative STAT3) significantly blocks 5-HT1A-receptor-mediated neurite outgrowth;	15925428
hsa04014	Ras signaling pathway	Blood mononuclear cell proteome suggests integrin and Ras signaling as critical pathways for antidepressant treatment response;	24607422
		Ras-GRF proteins contribute to forms of synaptic plasticity that are required specifically for mature hippocampal function;	16467520
		RAS-GRF1 mediates NMDA-type glutamate receptor (NMDAR)-induction of long term depression in the CA1 region of the hippocampus of mice	23766509

It is undeniable that there are several limitations in this study. First, it is not sufficient to study based on the data currently available, because the technique of identifying metabolites for depression is still a continuous improvement process. Second, although we have used published literature to verify some results, validation of molecular mechanisms based on clinical samples are necessary to analysis the complex pathogenesis of depression. Our results provide good ideas and methods for studying the pathogenesis of depression.

## Conclusion

We identified 36 metabolic biomarkers of clinical plasma metabolomics using NMR and MS. The relationship between biomarkers and enzymes were collected from the HMDB database. The results show that stearic acid, phytosphingosine, glycine, glutamine and phospholipids were important metabolic biomarkers. Hydrolase, transferase and acyltransferase involve the largest number of metabolic biomarkers. The important metabolites and enzymes screened by the topology of the network may play a key role in the underlying molecular mechanism of depression.

The nervous system, immune system and endocrine system are mainly involved in the underlying pathological mechanism of depression. The *DSWMP* index was used to assess the importance pathways of hub metabolic biomarkers involved. Nine proteins (TP53, IL1B, TNF, PTEN, HLA-DRB1, MTOR, HRAS, INS and PIK3CA) are widely involved in the nervous system, immune system and endocrine system. These targets may be important targets for antidepressants in the treatment of depression. Seven important pathways, such as PI3K-Akt signaling pathway and mTOR signaling pathway, are closely related to the pathogenesis molecular mechanisms of depression and require further investigation. A combination of network pharmacology strategy and metabolomics approach has great potentials in comprehensively and deeply understanding the molecular mechanism of depression. The application of important biomarkers and pathways in clinical practice may help to improve the diagnosis of depression and the evaluation of antidepressant effect, which provides important clues for the study of metabolic characteristics of depression.

## Data Availability Statement

All datasets generated for this study are included in the article/**Supplementary Material**.

## Author Contributions

X-MQ and J-ST provided the concept and designed the study. YG, TX, Y-XZ, TL-H and S-BL conducted the analyses and wrote the manuscript. YG, TX, Y-XZ, TL-H and S-BL participated in data analysis. X-MQ and J-ST contributed to revise the manuscript. All authors contributed to the article and approved the submitted version.

## Funding

This work was supported by the National S&T Major Projects for “Major New Drugs Innovation and Development” (2017ZX09301047), the Science and Technology of Shanxi Province (No. 201701D121137 and No. 201903D321210).

## Conflict of Interest

The authors declare that the research was conducted in the absence of any commercial or financial relationships that could be construed as a potential conflict of interest.

## Abbreviations

^1^H-NMR, Proton nuclear magnetic resonance; APT-2, Acyl-protein thioesterase 2; ATP, Adenosine triphosphate; CMS, Chronic mild stress; CNS, Central nervous system; CoA, Acetyl-coenzyme A; CPLA2, Cytosolic phospholipase A2; EPA, Eicosapentaenoic acid; DSWP, Docking score-weighted polypharmacological index; HMDB, Human Metabolome Database; HPA, Hypothalamic pituitary adrenal; HRAS, HRas Proto-Oncogene; LC–MS, Liquid chromatography–mass spectrometry; GC–MS, Gas chromatography–mass spectrometer; MEI, Metabolic Biomarker–enzyme interactions; PTEN, Phosphatase and tensin homolog; MTI, Metabolic Biomarker–target interactions; MTOR, Rapamycin Kinase; PIK3CA, Phosphatidylinositol 4, 5-bisphosphate 3-kinase catalytic subunit alpha isoform; PINE, Psycho-immune-neuroendocrine; TP53, Tumor Protein P53; TNF, Tumor necrosis factor.
