# Supplementary material for: A Novel Network Pharmacology Strategy to Decode Metabolic Biomarkers and Targets Interactions for Depression
Source: Front Psychiatry. 2020 Jul 15;11:667. doi: 10.3389/fpsyt.2020.00667 (PMC7373779; doi:10.3389/fpsyt.2020.00667)
Supplement: Supplementary file 1 [file DataSheet_1.pdf]

**Supplementary Table S1. Targets of nervous system**

| UniProt ID | UniProt Name |
|---|---|
| O00255 | Menin 1 |
| O00555 | Calcium Voltage-Gated Channel Subunit Alpha1 A |
| O14497 | AT-Rich Interaction Domain 1A |
| O15146 | Muscle Associated Receptor Tyrosine Kinase |
| O43612 | Hypocretin Neuropeptide Precursor |
| O60260 | Parkin RBR E3 Ubiquitin Protein Ligase |
| O60733 | Phospholipase A2 Group VI |
| O75665 | OFD1, Centriole And Centriolar Satellite Protein |
| O95409 | Zic Family Member 2 |
| P00414 | Mitochondrially Encoded Cytochrome C Oxidase III |
| P00441 | Superoxide Dismutase 1 |
| P00450 | Ceruloplasmin |
| P01112 | HRas Proto-Oncogene, GTPase |
| P01116 | KRAS Proto-Oncogene, GTPase |
| P01127 | Platelet Derived Growth Factor Subunit B |
| P01138 | Nerve Growth Factor |
| P01189 | Proopiomelanocortin |
| P01213 | Prodynorphin |
| P01303 | Neuropeptide Y |
| P01308 | Insulin |
| P01375 | Tumor Necrosis Factor |
| P01579 | Interferon Gamma |
| P01584 | Interleukin 1 Beta |
| P01911 | Major Histocompatibility Complex, Class II, DR Beta 1 |
| P01920 | Major Histocompatibility Complex, Class II, DQ Beta 1 |
| P02649 | Apolipoprotein E |
| P02768 | Albumin |
| P03886 | Mitochondrially Encoded NADH:Ubiquinone Oxidoreductase Core Subunit 1 |
| P03915 | Mitochondrially Encoded NADH:Ubiquinone Oxidoreductase Core Subunit 5 |
| P03923 | Mitochondrially Encoded NADH:Ubiquinone Oxidoreductase Core Subunit 6 |
| P04062 | Glucosylceramidase Beta |
| P04156 | Prion Protein |
| P04271 | S100 Calcium Binding Protein B |
| P04637 | Tumor Protein P53 |
| P05019 | Insulin Like Growth Factor 1 |
| P05067 | Amyloid Beta Precursor Protein |
| P05231 | Interleukin 6 |
| P06280 | Galactosidase Alpha |
| P06850 | Corticotropin Releasing Hormone |
| P06881 | Calcitonin Related Polypeptide Alpha |
| P07101 | Tyrosine Hydroxylase |
| P07949 | Ret Proto-Oncogene |
| P08908 | 5-Hydroxytryptamine Receptor 1A |
| P09172 | Dopamine Beta-Hydroxylase |
| P09619 | Platelet Derived Growth Factor Receptor Beta |
| P10071 | GLI Family Zinc Finger 3 |
| P10636 | Microtubule Associated Protein Tau |
| P11166 | Solute Carrier Family 2 Member 1 |
| P11362 | Fibroblast Growth Factor Receptor 1 |
| P12821 | Angiotensin I Converting Enzyme |
| P13637 | ATPase Na+/K+ Transporting Subunit Alpha 3 |
| P14136 | Glial Fibrillary Acidic Protein |
| P14416 | Dopamine Receptor D2 |
| P15056 | B-Raf Proto-Oncogene, Serine/Threonine Kinase |
| P15104 | Glutamate-Ammonia Ligase |
| P15289 | Arylsulfatase A |
| P15884 | Transcription Factor 4 |
| P16278 | Galactosidase Beta 1 |
| P20226 | TATA-Box Binding Protein |

| | |
|---|---|
| P20700 | Lamin B1 |
| P20783 | Neurotrophin 3 |
| P21333 | Filamin A |
| P21359 | Neurofibromin 1 |
| P21397 | Monoamine Oxidase A |
| P21917 | Dopamine Receptor D4 |
| P21964 | Catechol-O-Methyltransferase |
| P22301 | Interleukin 10 |
| P22466 | Galanin And GMAP Prepropeptide |
| P22607 | Fibroblast Growth Factor Receptor 3 |
| P23560 | Brain Derived Neurotrophic Factor |
| P23760 | Paired Box 3 |
| P23975 | Solute Carrier Family 6 Member 2 |
| P26358 | DNA Methyltransferase 1 |
| P26367 | Paired Box 6 |
| P27338 | Monoamine Oxidase B |
| P28329 | Choline O-Acetyltransferase |
| P28799 | Granulin Precursor |
| P29475 | Nitric Oxide Synthase 1 |
| P29992 | G Protein Subunit Alpha 11 |
| P30793 | GTP Cyclohydrolase 1 |
| P31645 | Solute Carrier Family 6 Member 4 |
| P31749 | AKT Serine/Threonine Kinase 1 |
| P32243 | Orthodenticle Homeobox 2 |
| P35520 | Cystathionine-Beta-Synthase |
| P35637 | FUS RNA Binding Protein |
| P35716 | SRY-Box 11 |
| P37840 | Synuclein Alpha |
| P39905 | Glial Cell Derived Neurotrophic Factor |
| P41159 | Leptin |
| P42336 | Phosphatidylinositol-4,5-Bisphosphate 3-Kinase Catalytic Subunit Alpha |
| P42345 | Mechanistic Target Of Rapamycin Kinase |
| P42858 | Huntingtin |
| P42898 | Methylenetetrahydrofolate Reductase |
| P43004 | Solute Carrier Family 1 Member 2 |
| P43354 | Nuclear Receptor Subfamily 4 Group A Member 2 |
| P48436 | SRY-Box 9 |
| P49768 | Presenilin 1 |
| P51532 | SWI/SNF Related, Matrix Associated, Actin Dependent Regulator Of Chromatin, Subfamily A, Me |
| P51608 | Methyl-CpG Binding Protein 2 |
| P51659 | Hydroxysteroid 17-Beta Dehydrogenase 4 |
| P53667 | LIM Domain Kinase 1 |
| P54098 | DNA Polymerase Gamma, Catalytic Subunit |
| P55072 | Valosin Containing Protein |
| P55075 | Fibroblast Growth Factor 8 |
| P55316 | Forkhead Box G1 |
| P60484 | Phosphatase And Tensin Homolog |
| P61278 | Somatostatin |
| P78509 | Reelin |
| P98164 | LDL Receptor Related Protein 2 |
| Q01959 | Solute Carrier Family 6 Member 3 |
| Q05940 | Solute Carrier Family 18 Member A2 |
| Q06413 | Myocyte Enhancer Factor 2C |
| Q06787 | Fragile X Mental Retardation 1 |
| Q12824 | SWI/SNF Related, Matrix Associated, Actin Dependent Regulator Of Chromatin, Subfamily B, Me |
| Q12879 | Glutamate Ionotropic Receptor NMDA Type Subunit 2A |
| Q13148 | TAR DNA Binding Protein |
| Q13224 | Glutamate Ionotropic Receptor NMDA Type Subunit 2B |
| Q13255 | Glutamate Metabotropic Receptor 1 |
| Q13501 | Sequestosome 1 |
| Q13635 | Patched 1 |

| | |
|---|---|
| Q13702 | Receptor Associated Protein Of The Synapse |
| Q14203 | Dynactin Subunit 1 |
| Q15067 | Acyl-CoA Oxidase 1 |
| Q15303 | Erb-B2 Receptor Tyrosine Kinase 4 |
| Q15465 | Sonic Hedgehog |
| Q16620 | Neurotrophic Receptor Tyrosine Kinase 2 |
| Q5JTZ9 | Alanyl-TRNA Synthetase 2, Mitochondrial |
| Q5S007 | Leucine Rich Repeat Kinase 2 |
| Q8NER1 | Transient Receptor Potential Cation Channel Subfamily V Member 1 |
| Q8NFD5 | AT-Rich Interaction Domain 1B |
| Q969G3 | SWI/SNF Related, Matrix Associated, Actin Dependent Regulator Of Chromatin, Subfamily E, Me |
| Q96CV9 | Optineurin |
| Q96LT7 | Chromosome 9 Open Reading Frame 72 |
| Q96RR1 | Twinkle MtDNA Helicase |
| Q99259 | Glutamate Decarboxylase 1 |
| Q99497 | Parkinsonism Associated Deglycase |
| Q99700 | Ataxin 2 |
| Q9BYW2 | SET Domain Containing 2 |
| Q9BZ23 | Pantothenate Kinase 2 |
| Q9HBA0 | Transient Receptor Potential Cation Channel Subfamily V Member 4 |
| Q9NQC3 | Reticulon 4 |
| Q9NRA2 | Solute Carrier Family 17 Member 5 |
| Q9NSK7 | Chromosome 19 Open Reading Frame 12 |
| Q9NWW5 | CLN6, Transmembrane ER Protein |
| Q9P0N5 | Transmembrane Protein 216 |
| Q9UBK2 | PPARG Coactivator 1 Alpha |
| Q9UBP0 | Spastin |
| Q9UHD2 | TANK Binding Kinase 1 |
| Q9UM47 | Notch 3 |
| Q9UQN3 | Charged Multivesicular Body Protein 2B |
| Q9Y243 | AKT Serine/Threonine Kinase 3 |
| Q9Y276 | BCS1 Homolog, Ubiquinol-Cytochrome C Reductase Complex Chaperone |
| Q9Y617 | Phosphoserine Aminotransferase 1 |
| Q9Y6H5 | Synuclein Alpha Interacting Protein |
| Q9Y6K9 | Inhibitor Of Nuclear Factor Kappa B Kinase Subunit Gamma |

**Supplementary Table S2. Targets of immune system**

| UniProt ID | UniProt name |
|---|---|
| P02787 | Transferrin |
| P07202 | Thyroid Peroxidase |
| P98160 | Heparan Sulfate Proteoglycan 2 |
| Q06124 | Protein Tyrosine Phosphatase, Non-Receptor Type 11 |
| Q6UXH8 | Collagen And Calcium Binding EGF Domains 1 |
| O15146 | Muscle Associated Receptor Tyrosine Kinase |
| O43435 | T-Box 1 |
| O60934 | Nibrin |
| P00738 | Haptoglobin |
| P01112 | HRas Proto-Oncogene, GTPase |
| P01116 | KRAS Proto-Oncogene, GTPase |
| P01189 | Proopiomelanocortin |
| P01236 | Prolactin |
| P01308 | Insulin |
| P01375 | Tumor Necrosis Factor |
| P01562 | Interferon Alpha 1 |
| P01579 | Interferon Gamma |
| P01584 | Interleukin 1 Beta |
| P01589 | Interleukin 2 Receptor Subunit Alpha |
| P01911 | Major Histocompatibility Complex, Class II, DR Beta 1 |
| P01920 | Major Histocompatibility Complex, Class II, DQ Beta 1 |
| P02649 | Apolipoprotein E |
| P02741 | C-Reactive Protein |
| P02768 | Albumin |
| P04049 | Raf-1 Proto-Oncogene, Serine/Threonine Kinase |
| P04062 | Glucosylceramidase Beta |
| P04150 | Nuclear Receptor Subfamily 3 Group C Member 1 |
| P04637 | Tumor Protein P53 |
| P05231 | Interleukin 6 |
| P10145 | C-X-C Motif Chemokine Ligand 8 |
| P11274 | BCR, RhoGEF And GTPase Activating Protein |
| P14902 | Indoleamine 2,3-Dioxygenase 1 |
| P15056 | B-Raf Proto-Oncogene, Serine/Threonine Kinase |
| P15976 | GATA Binding Protein 1 |
| P22301 | Interleukin 10 |
| P22607 | Fibroblast Growth Factor Receptor 3 |
| P23771 | GATA Binding Protein 3 |
| P27986 | Phosphoinositide-3-Kinase Regulatory Subunit 1 |
| P28482 | Mitogen-Activated Protein Kinase 1 |
| P31749 | AKT Serine/Threonine Kinase 1 |
| P40200 | CD96 Molecule |
| P41159 | Leptin |
| P42336 | Phosphatidylinositol-4,5-Bisphosphate 3-Kinase Catalytic Subunit Alpha |
| P42345 | Mechanistic Target Of Rapamycin Kinase |
| P49913 | Cathelicidin Antimicrobial Peptide |
| P49917 | DNA Ligase 4 |
| P60484 | Phosphatase And Tensin Homolog |
| P60568 | Interleukin 2 |
| P62834 | RAP1A, Member Of RAS Oncogene Family |
| P68871 | Hemoglobin Subunit Beta |
| Q13936 | Calcium Voltage-Gated Channel Subunit Alpha1 C |
| Q14739 | Lamin B Receptor |
| Q99572 | Purinergic Receptor P2X 7 |
| Q9NRZ9 | Helicase, Lymphoid Specific |
| Q9NZC2 | Triggering Receptor Expressed On Myeloid Cells 2 |
| Q9P2D1 | Chromodomain Helicase DNA Binding Protein 7 |
| Q9UHD2 | TANK Binding Kinase 1 |
| Q9Y2R2 | Protein Tyrosine Phosphatase, Non-Receptor Type 22 |
| Q9Y6K9 | Inhibitor Of Nuclear Factor Kappa B Kinase Subunit Gamma |

**Supplementary Table S3. Targets of endocrine system**

| UniProt ID | UniProt name |
|---|---|
| O00170 | Aryl Hydrocarbon Receptor Interacting Protein |
| O00192 | ARVCF, Delta Catenin Family Member |
| O00230 | Cortistatin |
| O00255 | Menin 1 |
| O14497 | AT-Rich Interaction Domain 1A |
| O14662 | Syntaxin 16 |
| O43435 | T-Box 1 |
| O43933 | Peroxisomal Biogenesis Factor 1 |
| O75360 | PROP Paired-Like Homeobox 1 |
| O76024 | Wolframin ER Transmembrane Glycoprotein |
| O94972 | Tripartite Motif Containing 37 |
| P01112 | HRas Proto-Oncogene, GTPase |
| P01116 | KRAS Proto-Oncogene, GTPase |
| P01138 | Nerve Growth Factor |
| P01148 | Gonadotropin Releasing Hormone 1 |
| P01185 | Arginine Vasopressin |
| P01189 | Proopiomelanocortin |
| P01222 | Thyroid Stimulating Hormone Beta |
| P01236 | Prolactin |
| P01241 | Growth Hormone 1 |
| P01270 | Parathyroid Hormone |
| P01303 | Neuropeptide Y |
| P01308 | Insulin |
| P01375 | Tumor Necrosis Factor |
| P01579 | Interferon Gamma |
| P01584 | Interleukin 1 Beta |
| P01589 | Interleukin 2 Receptor Subunit Alpha |
| P01911 | Major Histocompatibility Complex, Class II, DR Beta 1 |
| P01920 | Major Histocompatibility Complex, Class II, DQ Beta 1 |
| P02741 | C-Reactive Protein |
| P02768 | Albumin |
| P02787 | Transferrin |
| P03372 | Estrogen Receptor 1 |
| P04049 | Raf-1 Proto-Oncogene, Serine/Threonine Kinase |
| P04150 | Nuclear Receptor Subfamily 3 Group C Member 1 |
| P04278 | Sex Hormone Binding Globulin |
| P04637 | Tumor Protein P53 |
| P05019 | Insulin Like Growth Factor 1 |
| P05231 | Interleukin 6 |
| P06307 | Cholecystokinin |
| P06850 | Corticotropin Releasing Hormone |
| P06881 | Calcitonin Related Polypeptide Alpha |
| P07101 | Tyrosine Hydroxylase |
| P07202 | Thyroid Peroxidase |
| P07949 | Ret Proto-Oncogene |
| P08069 | Insulin Like Growth Factor 1 Receptor |
| P08397 | Hydroxymethylbilane Synthase |
| P08833 | Insulin Like Growth Factor Binding Protein 1 |
| P09619 | Platelet Derived Growth Factor Receptor Beta |
| P10071 | GLI Family Zinc Finger 3 |
| P10082 | Peptide YY |
| P10145 | C-X-C Motif Chemokine Ligand 8 |
| P10644 | Protein Kinase CAMP-Dependent Type I Regulatory Subunit Alpha |
| P10912 | Growth Hormone Receptor |
| P11166 | Solute Carrier Family 2 Member 1 |
| P11362 | Fibroblast Growth Factor Receptor 1 |
| P12821 | Angiotensin I Converting Enzyme |
| P14136 | Glial Fibrillary Acidic Protein |
| P14416 | Dopamine Receptor D2 |

| | |
|---|---|
| P15056 | B-Raf Proto-Oncogene, Serine/Threonine Kinase |
| P16220 | CAMP Responsive Element Binding Protein 1 |
| P16435 | Cytochrome P450 Oxidoreductase |
| P17302 | Gap Junction Protein Alpha 1 |
| P17612 | Protein Kinase CAMP-Activated Catalytic Subunit Alpha |
| P20396 | Thyrotropin Releasing Hormone |
| P21359 | Neurofibromin 1 |
| P21802 | Fibroblast Growth Factor Receptor 2 |
| P21964 | Catechol-O-Methyltransferase |
| P22466 | Galanin And GMAP Prepropeptide |
| P23352 | Anosmin 1 |
| P23771 | GATA Binding Protein 3 |
| P26367 | Paired Box 6 |
| P27361 | Mitogen-Activated Protein Kinase 3 |
| P27487 | Dipeptidyl Peptidase 4 |
| P27986 | Phosphoinositide-3-Kinase Regulatory Subunit 1 |
| P28069 | POU Class 1 Homeobox 1 |
| P28482 | Mitogen-Activated Protein Kinase 1 |
| P30968 | Gonadotropin Releasing Hormone Receptor |
| P31749 | AKT Serine/Threonine Kinase 1 |
| P36551 | Coproporphyrinogen Oxidase |
| P39905 | Glial Cell Derived Neurotrophic Factor |
| P41159 | Leptin |
| P41180 | Calcium Sensing Receptor |
| P42336 | Phosphatidylinositol-4,5-Bisphosphate 3-Kinase Catalytic Subunit Alpha |
| P42345 | Mechanistic Target Of Rapamycin Kinase |
| P42898 | Methylenetetrahydrofolate Reductase |
| P48436 | SRY-Box 9 |
| P51532 | SWI/SNF Related, Matrix Associated, Actin Dependent Regulator Of Chromatin, Subfam |
| P51654 | Glypican 3 |
| P51659 | Hydroxysteroid 17-Beta Dehydrogenase 4 |
| P54098 | DNA Polymerase Gamma, Catalytic Subunit |
| P54198 | Histone Cell Cycle Regulator |
| P55075 | Fibroblast Growth Factor 8 |
| P60484 | Phosphatase And Tensin Homolog |
| P60568 | Interleukin 2 |
| P61278 | Somatostatin |
| P63092 | GNAS Complex Locus |
| P78504 | Jagged 1 |
| Q02750 | Mitogen-Activated Protein Kinase Kinase 1 |
| Q03431 | Parathyroid Hormone 1 Receptor |
| Q06124 | Protein Tyrosine Phosphatase, Non-Receptor Type 11 |
| Q06787 | Fragile X Mental Retardation 1 |
| Q08499 | Phosphodiesterase 4D |
| Q13635 | Patched 1 |
| Q14739 | Lamin B Receptor |
| Q14999 | Cullin 7 |
| Q15465 | Sonic Hedgehog |
| Q15652 | Jumonji Domain Containing 1C |
| Q15726 | KiSS-1 Metastasis Suppressor |
| Q30201 | Hemochromatosis |
| Q7LG56 | Ribonucleotide Reductase Regulatory TP53 Inducible Subunit M2B |
| Q8NEA6 | GLIS Family Zinc Finger 3 |
| Q8NFD5 | AT-Rich Interaction Domain 1B |
| Q8NFJ6 | Prokineticin Receptor 2 |
| Q969F8 | KISS1 Receptor |
| Q96L73 | Nuclear Receptor Binding SET Domain Protein 1 |
| Q96RR1 | Twinkle MtDNA Helicase |
| Q99259 | Glutamate Decarboxylase 1 |
| Q9H251 | Cadherin Related 23 |
| Q9H3D4 | Tumor Protein P63 |

| | |
|---|---|
| Q9HC23 | Prokineticin 2 |
| Q9HCR9 | Phosphodiesterase 11A |
| Q9P2D1 | Chromodomain Helicase DNA Binding Protein 7 |
| Q9UBU3 | Ghrelin And Obestatin Prepropeptide |
| Q9Y2R2 | Protein Tyrosine Phosphatase, Non-Receptor Type 22 |

**Supplementary Table S4. The information of enzymes for metabonomic biomarkers**

| UniProt | Gene ID | Kegg Gene | Name | Gene | Organism | Status | Function |
|---|---|---|---|---|---|---|---|
| A5PLL0 | 1375 | has1375 | CPT1B protein | CPT1B | Homo sapiens (Human) | Unreviewed(TrEMBL) | Acyltransferase |
| A6NH11 | | | Glycolipid transfer protein domain-containing prote | GLTPD2 | Homo sapiens (Human) | Reviewed(Swiss-Prot) | Intermembrane lipid transfer activity |
| A8YXX4 | 2752 | has2752 | Glutamine synthetase | PIG59 | Homo sapiens (Human) | Unreviewed(TrEMBL) | Ligase |
| B5MDU3 | | | Pleckstrin homology domain-containing family A m | PLEKHA8 | Homo sapiens (Human) | Unreviewed(TrEMBL) | Intermembrane lipid transfer activity |
| O00154 | 11332 | has11332 | Cytosolic acyl coenzyme A thioester hydrolase | ACOT7 | Homo sapiens (Human) | Reviewed(Swiss-Prot) | Hydrolase, Serine esterase |
| O00748 | 8824 | has8824 | Cocaine esterase | CES2 | Homo sapiens (Human) | Reviewed(Swiss-Prot) | Hydrolase, Serine esterase |
| O14734 | 10005 | has10005 | Acyl-coenzyme A thioesterase 8 | ACOT8 | Homo sapiens (Human) | Reviewed(Swiss-Prot) | Hydrolase, Serine esterase |
| O14939 | 5338 | has5338 | Phospholipase D2 | PLD2 | Homo sapiens (Human) | Reviewed(Swiss-Prot) | Hydrolase |
| O15067 | 5198 | has5198 | Phosphoribosylformylglycinamidine synthase | PFAS | Homo sapiens (Human) | Reviewed(Swiss-Prot) | Ligase |
| O15245 | | | Solute carrier family 22 member 1 | SLC22A1 | Homo sapiens (Human) | Reviewed(Swiss-Prot) | Acetylcholine transmembrane transpo |
| O15382 | 587 | has587 | Branched-chain-amino-acid aminotransferase, mito | BCAT2 | Homo sapiens (Human) | Reviewed(Swiss-Prot) | Aminotransferase, Transferase |
| O15496 | 8399 | has8399 | Group 10 secretory phospholipase A2 | PLA2G10 | Homo sapiens (Human) | Reviewed(Swiss-Prot) | Hydrolase |
| O43280 | 11181 | has11181 | Trehalase | TREH | Homo sapiens (Human) | Reviewed(Swiss-Prot) | Glycosidase, Hydrolase |
| O43292 | 8733 | has8733 | Glycosylphosphatidylinositol anchor attachment 1 p | GPAA1 | Homo sapiens (Human) | Reviewed(Swiss-Prot) | Tubulin binding |
| O43451 | 8972 | has8972 | Maltase-glucoamylase, intestinal | MGAM | Homo sapiens (Human) | Reviewed(Swiss-Prot) | Glycosidase, Hydrolase, Multifunctic |
| O43548 | 9333 | has9333 | Protein-glutamine gamma-glutamyltransferase 5 | TGM5 | Homo sapiens (Human) | Reviewed(Swiss-Prot) | Acyltransferase, Transferase |
| O43716 | 283459 | has283459 | Glutamyl-tRNA(Gln) amidotransferase subunit C, r | GATC | Homo sapiens (Human) | Reviewed(Swiss-Prot) | Ligase |
| O60391 | 116444 | has116444 | Glutamate receptor ionotropic, NMDA 3B | GRIN3B | Homo sapiens (Human) | Reviewed(Swiss-Prot) | Ion channel, Ligand-gated ion channe |
| O60733 | 8398 | has8398 | 85/88 kDa calcium-independent phospholipase A2 | PLA2G6 | Homo sapiens (Human) | Reviewed(Swiss-Prot) | Calmodulin-binding, Hydrolase |
| O60909 | 8704 | has8704 | Beta-1,4-galactosyltransferase 2 | B4GALT2 | Homo sapiens (Human) | Reviewed(Swiss-Prot) | Glycosyltransferase, Transferase |
| O75311 | 8001 | has8001 | Glycine receptor subunit alpha-3 | GLRA3 | Homo sapiens (Human) | Reviewed(Swiss-Prot) | Chloride channel, Ion channel, Ligan |
| O75390 | 1431 | has1431 | Citrate synthase, mitochondrial | CS | Homo sapiens (Human) | Reviewed(Swiss-Prot) | |
| O75594 | 8993 | has8993 | Peptidoglycan recognition protein 1 | PGLYRP1 | Homo sapiens (Human) | Reviewed(Swiss-Prot) | Antibiotic, Antimicrobial |
| O75600 | 23464 | has23464 | 2-amino-3-ketobutyrate coenzyme A ligase, mitoch | GCAT | Homo sapiens (Human) | Reviewed(Swiss-Prot) | Acyltransferase, Transferase |
| O75608 | 10434 | has10434 | Acyl-protein thioesterase 1 | LYPLA1 | Homo sapiens (Human) | Reviewed(Swiss-Prot) | Hydrolase |
| O75879 | 5188 | has5188 | Glutamyl-tRNA(Gln) amidotransferase subunit B, r | PET112 | Homo sapiens (Human) | Reviewed(Swiss-Prot) | Ligase |
| O76074 | 8654 | has8654 | cGMP-specific 3',5'-cyclic phosphodiesterase | PDE5A | Homo sapiens (Human) | Reviewed(Swiss-Prot) | Allosteric enzyme, Hydrolase |
| O94808 | 9945 | has9945 | Glutamine--fructose-6-phosphate aminotransferase | GFPT2 | Homo sapiens (Human) | Reviewed(Swiss-Prot) | Aminotransferase, Transferase |
| O94925 | 2744 | has2744 | Glutaminase kidney isoform, mitochondrial | GLS | Homo sapiens (Human) | Reviewed(Swiss-Prot) | Hydrolase |
| O94956 | 11309 | has11309 | Solute carrier organic anion transporter family mem | SLCO2B1 | Homo sapiens (Human) | Reviewed(Swiss-Prot) | Bile acid transmembrane transporter |
| O95363 | 10667 | has10667 | Phenylalanine--tRNA ligase, mitochondrial | FARS2 | Homo sapiens (Human) | Reviewed(Swiss-Prot) | Aminoacyl-tRNA synthetase, Ligase |
| O95372 | 11313 | has11313 | Acyl-protein thioesterase 2 | LYPLA2 | Homo sapiens (Human) | Reviewed(Swiss-Prot) | Hydrolase |
| O95397 | | | Protein PLEKHA9 | PLEKHA9 | Homo sapiens (Human) | Reviewed(Swiss-Prot) | Intermembrane lipid transfer activity |
| O95427 | 23556 | has23556 | GPI ethanolamine phosphate transferase 1 | PIGN | Homo sapiens (Human) | Reviewed(Swiss-Prot) | Transferase |
| O95932 | 343641 | has343641 | Protein-glutamine gamma-glutamyltransferase 6 | TGM6 | Homo sapiens (Human) | Reviewed(Swiss-Prot) | Acyltransferase, Transferase |
| P00325 | 125 | has125 | Alcohol dehydrogenase 1B | ADH1B | Homo sapiens (Human) | Reviewed(Swiss-Prot) | Oxidoreductase |
| P00326 | 126 | has126 | Alcohol dehydrogenase 1C | ADH1C | Homo sapiens (Human) | Reviewed(Swiss-Prot) | Oxidoreductase |
| P00338 | 3939 | has3939 | L-lactate dehydrogenase A chain | LDHA | Homo sapiens (Human) | Reviewed(Swiss-Prot) | Oxidoreductase |
| P00439 | 5053 | has5053 | Phenylalanine-4-hydroxylase | PAH | Homo sapiens (Human) | Reviewed(Swiss-Prot) | Allosteric enzyme, Monooxygenase, |
| P00488 | 2162 | has2162 | Coagulation factor XIII A chain | F13A1 | Homo sapiens (Human) | Reviewed(Swiss-Prot) | Acyltransferase, Transferase |
| P00505 | 2806 | has2806 | Aspartate aminotransferase, mitochondrial | GOT2 | Homo sapiens (Human) | Reviewed(Swiss-Prot) | Aminotransferase, Transferase |
| P00709 | 3906 | has3906 | Alpha-lactalbumin | LALBA | Homo sapiens (Human) | Reviewed(Swiss-Prot) | Milk protein |
| P00918 | 760 | has760 | Carbonic anhydrase 2 | CA2 | Homo sapiens (Human) | Reviewed(Swiss-Prot) | Lyase |
| P03950 | 283 | has283 | Angiogenin | ANG | Homo sapiens (Human) | Reviewed(Swiss-Prot) | Actin binding |
| P04054 | 5319 | has5319 | Phospholipase A2 | PLA2G1B | Homo sapiens (Human) | Reviewed(Swiss-Prot) | Function Hydrolase |
| P04062 | 2629 | has2629 | Glucosylceramidase | GBA | Homo sapiens (Human) | Reviewed(Swiss-Prot) | Glycosidase, Hydrolase |

| | | | | | | | |
|---|---|---|---|---|---|---|---|
| P04180 | 3931 | has3931 | Phosphatidylcholine-sterol acyltransferase | LCAT | Homo sapiens (Human) | Reviewed(Swiss-Prot) | Acyltransferase, Transferase |
| P05089 | 383 | has383 | Arginase-1 | ARG1 | Homo sapiens (Human) | Reviewed(Swiss-Prot) | Hydrolase |
| P05091 | 217 | has217 | Aldehyde dehydrogenase, mitochondrial | ALDH2 | Homo sapiens (Human) | Reviewed(Swiss-Prot) | Oxidoreductase |
| P05164 | 4353 | has4353 | Myeloperoxidase | MPO | Homo sapiens (Human) | Reviewed(Swiss-Prot) | Oxidoreductase, Peroxidase |
| P05166 | 5096 | has5096 | Propionyl-CoA carboxylase beta chain, mitochondr | PCCB | Homo sapiens (Human) | Reviewed(Swiss-Prot) | Ligase |
| P06126 | 909 | has909 | T-cell surface glycoprotein CD1a | CD1A | Homo sapiens (Human) | Reviewed(Swiss-Prot) | Beta-2-microglobulin binding |
| P06276 | 590 | has590 | Cholinesterase | BCHE | Homo sapiens (Human) | Reviewed(Swiss-Prot) | Hydrolase, Serine esterase |
| P06280 | 2717 | has2717 | Alpha-galactosidase A | GLA | Homo sapiens (Human) | Reviewed(Swiss-Prot) | Glycosidase, Hydrolase |
| P06744 | 2821 | has2821 | Glucose-6-phosphate isomerase | GPI | Homo sapiens (Human) | Reviewed(Swiss-Prot) | Cytokine, Growth factor, Isomerase |
| P06858 | 4023 | has4023 | Lipoprotein lipase | LPL | Homo sapiens (Human) | Reviewed(Swiss-Prot) | Heparin-binding, Hydrolase |
| P07098 | 8513 | has8513 | Gastric triacylglycerol lipase | LIPF | Homo sapiens (Human) | Reviewed(Swiss-Prot) | Hydrolase |
| P07101 | 7054 | has7054 | Tyrosine 3-monooxygenase | TH | Homo sapiens (Human) | Reviewed(Swiss-Prot) | Monooxygenase, Oxidoreductase |
| P07195 | 3945 | has3945 | L-lactate dehydrogenase B chain | LDHB | Homo sapiens (Human) | Reviewed(Swiss-Prot) | Oxidoreductase |
| P07311 | 97 | has97 | Acylphosphatase-1 | ACYP1 | Homo sapiens (Human) | Reviewed(Swiss-Prot) | Hydrolase |
| P07311 | 97 | has97 | Acylphosphatase-1 | ACYP1 | Homo sapiens (Human) | Reviewed(Swiss-Prot) | Hydrolase |
| P07327 | 124 | has124 | Alcohol dehydrogenase 1A | ADH1A | Homo sapiens (Human) | Reviewed(Swiss-Prot) | Oxidoreductase |
| P07741 | 353 | has353 | Adenine phosphoribosyltransferase | APRT | Homo sapiens (Human) | Reviewed(Swiss-Prot) | Glycosyltransferase, Transferase |
| P07864 | 3948 | has3948 | L-lactate dehydrogenase C chain | LDHC | Homo sapiens (Human) | Reviewed(Swiss-Prot) | Oxidoreductase |
| P07998 | 6035 | has6035 | Ribonuclease pancreatic | RNASE1 | Homo sapiens (Human) | Reviewed(Swiss-Prot) | Endonuclease activity |
| P08243 | 440 | has440 | Asparagine synthetase [glutamine-hydrolyzing] | ASNS | Homo sapiens (Human) | Reviewed(Swiss-Prot) | Ligase |
| P09848 | 3938 | has3938 | Lactase-phlorizin hydrolase | LCT | Homo sapiens (Human) | Reviewed(Swiss-Prot) | Glycosidase, Hydrolase, Multifunctic |
| P0C7U2 | | | Putative neutral ceramidase C | ASAH2C | Homo sapiens (Human) | Reviewed(Swiss-Prot) | |
| P0C869 | 1001370 | hsa:100137( | Cytosolic phospholipase A2 beta | PLA2G4B | Homo sapiens (Human) | Reviewed(Swiss-Prot) | Hydrolase |
| P10253 | 2548 | has2548 | Lysosomal alpha-glucosidase | GAA | Homo sapiens (Human) | Reviewed(Swiss-Prot) | Glycosidase, Hydrolase |
| P10265 | | has | HERV-K_5q33.3 provirus ancestral Pro protein | | Homo sapiens (Human) | Reviewed(Swiss-Prot) | Aspartyl protease, Hydrolase, Protea: |
| P11150 | 3990 | has3990 | Hepatic triacylglycerol lipase | LIPC | Homo sapiens (Human) | Reviewed(Swiss-Prot) | |
| P11166 | 6513 | has6513 | Solute carrier family 2, facilitated glucose transport | SLC2A1 | Homo sapiens (Human) | Reviewed(Swiss-Prot) | Dehydroascorbic acid transmembran |
| P11678 | 8288 | has8288 | Eosinophil peroxidase | EPX | Homo sapiens (Human) | Reviewed(Swiss-Prot) | Oxidoreductase, Peroxidase |
| P11766 | 128 | has128 | Alcohol dehydrogenase class-3 | ADH5 | Homo sapiens (Human) | Reviewed(Swiss-Prot) | Oxidoreductase |
| P12724 | 6037 | has6037 | Eosinophil cationic protein | RNASE3 | Homo sapiens (Human) | Reviewed(Swiss-Prot) | Endonuclease activity |
| P12931 | 6714 | has6714 | Proto-oncogene tyrosine-protein kinase Src | SRC | Homo sapiens (Human) | Reviewed(Swiss-Prot) | Kinase, Transferase, Tyrosine-protei |
| P12931 | 6714 | has6714 | Proto-oncogene tyrosine-protein kinase Src | SRC | Homo sapiens (Human) | Reviewed(Swiss-Prot) | ATP binding |
| P13196 | 211 | has211 | 5-aminolevulinate synthase, nonspecific, mitochonc | ALAS1 | Homo sapiens (Human) | Reviewed(Swiss-Prot) | Acyltransferase, Transferase |
| P14174 | 4282 | has4282 | Macrophage migration inhibitory factor | MIF | Homo sapiens (Human) | Reviewed(Swiss-Prot) | Chemoattractant activity |
| P14410 | 6476 | has6476 | Sucrase-isomaltase, intestinal | SI | Homo sapiens (Human) | Reviewed(Swiss-Prot) | Glycosidase, Hydrolase, Multifunctic |
| P14555 | 5320 | has5320 | Phospholipase A2, membrane associated | PLA2G2A | Homo sapiens (Human) | Reviewed(Swiss-Prot) | Hydrolase |
| P14621 | 98 | has98 | Acylphosphatase-2 | ACYP2 | Homo sapiens (Human) | Reviewed(Swiss-Prot) | Hydrolase |
| P14621 | 98 | has98 | Acylphosphatase-2 | ACYP2 | Homo sapiens (Human) | Reviewed(Swiss-Prot) | Hydrolase |
| P14920 | 1610 | has1610 | D-amino-acid oxidase | DAO | Homo sapiens (Human) | Reviewed(Swiss-Prot) | Oxidoreductase |
| P15086 | 1360 | has1360 | Carboxypeptidase B | CPB1 | Homo sapiens (Human) | Reviewed(Swiss-Prot) | Carboxypeptidase, Hydrolase, Metall |
| P15104 | 2752 | has2752 | Glutamine synthetase | GLUL | Homo sapiens (Human) | Reviewed(Swiss-Prot) | Ligase, Lyase |
| P15121 | 231 | has231 | Aldose reductase | AKR1B1 | Homo sapiens (Human) | Reviewed(Swiss-Prot) | Oxidoreductase |
| P15144 | 290 | has290 | Aminopeptidase N | ANPEP | Homo sapiens (Human) | Reviewed(Swiss-Prot) | Aminopeptidase, Developmental pro |
| P15291 | 2683 | has2683 | Beta-1,4-galactosyltransferase 1 | B4GALT1 | Homo sapiens (Human) | Reviewed(Swiss-Prot) | Glycosyltransferase, Transferase |
| P15812 | 913 | has913 | T-cell surface glycoprotein CD1e, membrane-assoc | CD1E | Homo sapiens (Human) | Reviewed(Swiss-Prot) | Beta-3-microglobulin binding |
| P15813 | 912 | has912 | Antigen-presenting glycoprotein CD1d | CD1D | Homo sapiens (Human) | Reviewed(Swiss-Prot) | Beta-4-microglobulin binding |
| P16233 | 5406 | has5406 | Pancreatic triacylglycerol lipase | PNLIP | Homo sapiens (Human) | Reviewed(Swiss-Prot) | Hydrolase |
| P17174 | | | Aspartate aminotransferase, cytoplasmic | GOT1 | Homo sapiens (Human) | Reviewed(Swiss-Prot) | Aminotransferase, Transferase |

| | | | | | | | |
|---|---|---|---|---|---|---|---|
| P17735 | 6898 | has6898 | Tyrosine aminotransferase | TAT | Homo sapiens (Human) | Reviewed(Swiss-Prot) | Aminotransferase, Transferase |
| P17812 | 1503 | has1503 | CTP synthase 1 | CTPS1 | Homo sapiens (Human) | Reviewed(Swiss-Prot) | Ligase |
| P17900 | 2760 | has2760 | Ganglioside GM2 activator | GM2A | Homo sapiens (Human) | Reviewed(Swiss-Prot) | Hydrolase |
| P18428 | 3929 | has3929 | Lipopolysaccharide-binding protein | LBP | Homo sapiens (Human) | Reviewed(Swiss-Prot) | Antibiotic, Antimicrobial |
| P19367 | 3098 | has3098 | Hexokinase-1 | HK1 | Homo sapiens (Human) | Reviewed(Swiss-Prot) | Allosteric enzyme, Kinase, Transfera |
| P19835 | | | Bile salt-activated lipase | CEL | Homo sapiens (Human) | Reviewed(Swiss-Prot) | Hydrolase, Serine esterase |
| P20711 | 1644 | has1644 | Aromatic-L-amino-acid decarboxylase | DDC | Homo sapiens (Human) | Reviewed(Swiss-Prot) | Decarboxylase, Lyase |
| P21399 | 48 | has48 | Cytoplasmic aconitate hydratase | ACO1 | Homo sapiens (Human) | Reviewed(Swiss-Prot) | |
| P21549 | 189 | has189 | Serine--pyruvate aminotransferase | AGXT | Homo sapiens (Human) | Reviewed(Swiss-Prot) | Aminotransferase, Transferase |
| P21549 | 189 | has189 | Serine--pyruvate aminotransferase | AGXT | Homo sapiens (Human) | Reviewed(Swiss-Prot) | Aminotransferase, Transferase |
| P21964 | 1312 | has1312 | Catechol O-methyltransferase | COMT | Homo sapiens (Human) | Reviewed(Swiss-Prot) | Methyltransferase, Transferase |
| P21980 | 7052 | has7052 | Protein-glutamine gamma-glutamyltransferase 2 | TGM2 | Homo sapiens (Human) | Reviewed(Swiss-Prot) | Acyltransferase, Transferase |
| P22079 | 4025 | has4025 | Lactoperoxidase | LPO | Homo sapiens (Human) | Reviewed(Swiss-Prot) | Antimicrobial, Oxidoreductase, Pero: |
| P22102 | 2618 | has2618 | Trifunctional purine biosynthetic protein adenosine | GART | Homo sapiens (Human) | Reviewed(Swiss-Prot) | Ligase, Multifunctional enzyme, Trai |
| P22303 | 43 | has43 | Acetylcholinesterase | ACHE | Homo sapiens (Human) | Reviewed(Swiss-Prot) | Blood group antigen, Hydrolase, Seri |
| P22557 | 212 | has212 | 5-aminolevulinate synthase, erythroid-specific, mitc | ALAS2 | Homo sapiens (Human) | Reviewed(Swiss-Prot) | Acyltransferase, Transferase |
| P22735 | 7051 | has7051 | Protein-glutamine gamma-glutamyltransferase K | TGM1 | Homo sapiens (Human) | Reviewed(Swiss-Prot) | Acyltransferase, Transferase |
| P23141 | 1066 | has1066 | Liver carboxylesterase 1 | CES1 | Homo sapiens (Human) | Reviewed(Swiss-Prot) | Hydrolase, Serine esterase |
| P23378 | 2731 | has2731 | Glycine dehydrogenase (decarboxylating), mitocho | GLDC | Homo sapiens (Human) | Reviewed(Swiss-Prot) | Oxidoreductase |
| P23415 | 2741 | has2741 | Glycine receptor subunit alpha-1 | GLRA1 | Homo sapiens (Human) | Reviewed(Swiss-Prot) | Chloride channel, Ion channel, Ligan |
| P23416 | 2742 | has2742 | Glycine receptor subunit alpha-2 | GLRA2 | Homo sapiens (Human) | Reviewed(Swiss-Prot) | Chloride channel, Ion channel, Ligan |
| P23434 | 2653 | has2653 | Glycine cleavage system H protein, mitochondrial | GCSH | Homo sapiens (Human) | Reviewed(Swiss-Prot) | Aminomethyltransferase activity |
| P24298 | 2875 | has2875 | Alanine aminotransferase 1 | GPT | Homo sapiens (Human) | Reviewed(Swiss-Prot) | Aminotransferase, Transferase |
| P26640 | 7407 | has7407 | Valine--tRNA ligase | VARS | Homo sapiens (Human) | Reviewed(Swiss-Prot) | Aminoacyl-tRNA synthetase, Ligase |
| P27708 | 790 | has790 | CAD protein | CAD | Homo sapiens (Human) | Reviewed(Swiss-Prot) | Allosteric enzyme, Hydrolase, Ligase |
| P28329 | 1103 | has1103 | Choline O-acetyltransferase | CHAT | Homo sapiens (Human) | Reviewed(Swiss-Prot) | Acyltransferase, Transferase |
| P28332 | 130 | has130 | Alcohol dehydrogenase 6 | ADH6 | Homo sapiens (Human) | Reviewed(Swiss-Prot) | Oxidoreductase |
| P28838 | 51056 | has51056 | Cytosol aminopeptidase | LAP3 | Homo sapiens (Human) | Reviewed(Swiss-Prot) | Aminopeptidase, Hydrolase, Protease |
| P29016 | 910 | has910 | T-cell surface glycoprotein CD1b | CD1B | Homo sapiens (Human) | Reviewed(Swiss-Prot) | Beta-5-microglobulin binding |
| P29017 | 911 | has911 | T-cell surface glycoprotein CD1c | CD1C | Homo sapiens (Human) | Reviewed(Swiss-Prot) | Beta-6-microglobulin binding |
| P30041 | 9588 | has9588 | Peroxiredoxin-6 | PRDX6 | Homo sapiens (Human) | Reviewed(Swiss-Prot) | Antioxidant, Hydrolase, Multifunctio |
| P30837 | 219 | has219 | Aldehyde dehydrogenase X, mitochondrial | ALDH1B1 | Homo sapiens (Human) | Reviewed(Swiss-Prot) | Oxidoreductase |
| P31512 | 2329 | has2329 | Dimethylaniline monooxygenase [N-oxide-forming | FMO4 | Homo sapiens (Human) | Reviewed(Swiss-Prot) | Monooxygenase, Oxidoreductase |
| P31513 | 2328 | has2328 | Dimethylaniline monooxygenase [N-oxide-forming | FMO3 | Homo sapiens (Human) | Reviewed(Swiss-Prot) | Monooxygenase, Oxidoreductase |
| P32929 | 1491 | has1491 | Cystathionine gamma-lyase | CTH | Homo sapiens (Human) | Reviewed(Swiss-Prot) | Calmodulin-binding, Lyase |
| P34896 | 6470 | has6470 | Serine hydroxymethyltransferase, cytosolic | SHMT1 | Homo sapiens (Human) | Reviewed(Swiss-Prot) | Transferase |
| P34897 | 6472 | has6472 | Serine hydroxymethyltransferase, mitochondrial | SHMT2 | Homo sapiens (Human) | Reviewed(Swiss-Prot) | Transferase |
| P35222 | | | Catenin beta-1 | CTNNB1 | Homo sapiens (Human) | Reviewed(Swiss-Prot) | |
| P35520 | 875 | hsa:875 | Cystathionine beta-synthase | CBS | Homo sapiens (Human) | Reviewed(Swiss-Prot) | Allosteric enzyme, Lyase |
| P35557 | 2645 | has2645 | Glucokinase | GCK | Homo sapiens (Human) | Reviewed(Swiss-Prot) | Allosteric enzyme, Kinase, Transfera |
| P35573 | 178 | has178 | Glycogen debranching enzyme | AGL | Homo sapiens (Human) | Reviewed(Swiss-Prot) | Glycosidase, Glycosyltransferase, Hy |
| P35575 | 2538 | has2538 | Glucose-6-phosphatase | G6PC | Homo sapiens (Human) | Reviewed(Swiss-Prot) | Hydrolase |
| P35790 | 1119 | has1119 | Choline kinase alpha | CHKA | Homo sapiens (Human) | Reviewed(Swiss-Prot) | Kinase, Transferase |
| P36871 | 5236 | has5236 | Phosphoglucomutase-1 | PGM1 | Homo sapiens (Human) | Reviewed(Swiss-Prot) | Isomerase |
| P37231 | 5468 | has5468 | Peroxisome proliferator-activated receptor gamma | PPARG | Homo sapiens (Human) | Reviewed(Swiss-Prot) | |
| P37287 | 5277 | has5277 | Phosphatidylinositol N-acetylglucosaminyltransfera | PIGA | Homo sapiens (Human) | Reviewed(Swiss-Prot) | Glycosyltransferase, Transferase |
| P39877 | 5322 | has5322 | Calcium-dependent phospholipase A2 | PLA2G5 | Homo sapiens (Human) | Reviewed(Swiss-Prot) | Hydrolase |
| P40394 | 131 | has131 | Alcohol dehydrogenase class 4 mu/sigma chain | ADH7 | Homo sapiens (Human) | Reviewed(Swiss-Prot) | Oxidoreductase |

| | | | | | | | |
|---|---|---|---|---|---|---|---|
| P40926 | 4191 | has4191 | Malate dehydrogenase, mitochondrial | MDH2 | Homo sapiens (Human) | Reviewed(Swiss-Prot) | Oxidoreductase |
| P41247 | 8228 | has8228 | Patatin-like phospholipase domain-containing prote | PNPLA4 | Homo sapiens (Human) | Reviewed(Swiss-Prot) | Hydrolase |
| P41250 | 2617 | has2617 | Glycine--tRNA ligase | GARS | Homo sapiens (Human) | Reviewed(Swiss-Prot) | Aminoacyl-tRNA synthetase, Hydrol |
| P43007 | 6509 | has6509 | Neutral amino acid transporter A | SLC1A4 | Homo sapiens (Human) | Reviewed(Swiss-Prot) | Amino acid transmembrane transport |
| P45381 | 443 | has443 | Aspartoacylase | ASPA | Homo sapiens (Human) | Reviewed(Swiss-Prot) | Hydrolase |
| P47712 | 5321 | has5321 | Cytosolic phospholipase A2 | PLA2G4A | Homo sapiens (Human) | Reviewed(Swiss-Prot) | Hydrolase |
| P47895 | 220 | has220 | Aldehyde dehydrogenase family 1 member A3 | ALDH1A3 | Homo sapiens (Human) | Reviewed(Swiss-Prot) | Oxidoreductase |
| P47897 | 5859 | has5859 | Glutamine--tRNA ligase | QARS | Homo sapiens (Human) | Reviewed(Swiss-Prot) | Aminoacyl-tRNA synthetase, Ligase |
| P48067 | 6536 | has6536 | Sodium- and chloride-dependent glycine transporte | SLC6A9 | Homo sapiens (Human) | Reviewed(Swiss-Prot) | Glycine:sodium symporter activity |
| P48167 | 2743 | has2743 | Glycine receptor subunit beta | GLRB | Homo sapiens (Human) | Reviewed(Swiss-Prot) | Chloride channel, Ion channel, Ligan |
| P48637 | 2937 | has2937 | Glutathione synthetase | GSS | Homo sapiens (Human) | Reviewed(Swiss-Prot) | Ligase |
| P48728 | 275 | has275 | Aminomethyltransferase, mitochondrial | AMT | Homo sapiens (Human) | Reviewed(Swiss-Prot) | Aminotransferase, Transferase |
| P49189 | 223 | has223 | 4-trimethylaminobutyraldehyde dehydrogenase | ALDH9A1 | Homo sapiens (Human) | Reviewed(Swiss-Prot) | Oxidoreductase |
| P49221 | 7047 | has7047 | Protein-glutamine gamma-glutamyltransferase 4 | TGM4 | Homo sapiens (Human) | Reviewed(Swiss-Prot) | Acyltransferase, Transferase |
| P49326 | 2330 | has2330 | Dimethylaniline monooxygenase [N-oxide-forming | FMO5 | Homo sapiens (Human) | Reviewed(Swiss-Prot) | Monooxygenase, Oxidoreductase |
| P49327 | 2194 | has2194 | Fatty acid synthase | FASN | Homo sapiens (Human) | Reviewed(Swiss-Prot) | Hydrolase, Lyase, Multifunctional en |
| P49419 | 501 | has501 | Alpha-aminoadipic semialdehyde dehydrogenase | ALDH7A1 | Homo sapiens (Human) | Reviewed(Swiss-Prot) | Oxidoreductase |
| P49585 | 5130 | has5130 | Choline-phosphate cytidylyltransferase A | PCYT1A | Homo sapiens (Human) | Reviewed(Swiss-Prot) | Nucleotidyltransferase, Transferase |
| P49588 | 16 | has16 | Alanine--tRNA ligase, cytoplasmic | AARS | Homo sapiens (Human) | Reviewed(Swiss-Prot) | Aminoacyl-tRNA synthetase, Ligase, |
| P49753 | 10965 | has10965 | Acyl-coenzyme A thioesterase 2, mitochondrial | ACOT2 | Homo sapiens (Human) | Reviewed(Swiss-Prot) | Hydrolase, Serine esterase |
| P49915 | 8833 | has8833 | GMP synthase [glutamine-hydrolyzing] | GMPS | Homo sapiens (Human) | Reviewed(Swiss-Prot) | Ligase |
| P50440 | 2628 | has2628 | Glycine amidinotransferase, mitochondrial | GATM | Homo sapiens (Human) | Reviewed(Swiss-Prot) | Transferase |
| P51648 | 224 | has224 | Fatty aldehyde dehydrogenase | ALDH3A2 | Homo sapiens (Human) | Reviewed(Swiss-Prot) | Oxidoreductase |
| P52789 | 3099 | has3099 | Hexokinase-2 | HK2 | Homo sapiens (Human) | Reviewed(Swiss-Prot) | Allosteric enzyme, Kinase, Transfera |
| P52790 | 3101 | has3101 | Hexokinase-3 | HK3 | Homo sapiens (Human) | Reviewed(Swiss-Prot) | Allosteric enzyme, Kinase, Transfera |
| P53396 | 47 | has47 | ATP-citrate synthase | ACLY | Homo sapiens (Human) | Reviewed(Swiss-Prot) | ATP binding |
| P54315 | 5407 | has5407 | Inactive pancreatic lipase-related protein 1 | PNLIPRP1 | Homo sapiens (Human) | Reviewed(Swiss-Prot) | Calcium ion binding |
| P54317 | 5408 | has5408 | Pancreatic lipase-related protein 2 | PNLIPRP2 | Homo sapiens (Human) | Reviewed(Swiss-Prot) | Hydrolase |
| P54687 | 586 | has586 | Branched-chain-amino-acid aminotransferase, cyto | BCAT1 | Homo sapiens (Human) | Reviewed(Swiss-Prot) | Aminotransferase, Transferase |
| P57054 | 51227 | has51227 | Phosphatidylinositol N-acetylglucosaminyltransfera | PIGP | Homo sapiens (Human) | Reviewed(Swiss-Prot) | Glycosyltransferase, Transferase |
| P61916 | 10577 | has10577 | Epididymal secretory protein E1 | NPC2 | Homo sapiens (Human) | Reviewed(Swiss-Prot) | Cholesterol binding |
| P68402 | 5049 | has5049 | Platelet-activating factor acetylhydrolase IB subuni | PAFAH1B2 | Homo sapiens (Human) | Reviewed(Swiss-Prot) | Hydrolase |
| P78540 | 384 | has384 | Arginase-2, mitochondrial | ARG2 | Homo sapiens (Human) | Reviewed(Swiss-Prot) | Hydrolase |
| P80404 | 18 | has18 | 4-aminobutyrate aminotransferase, mitochondrial | ABAT | Homo sapiens (Human) | Reviewed(Swiss-Prot) | Aminotransferase, Transferase |
| Q01740 | 2326 | has2326 | Dimethylaniline monooxygenase [N-oxide-forming | FMO1 | Homo sapiens (Human) | Reviewed(Swiss-Prot) | Monooxygenase, Oxidoreductase |
| Q03154 | 95 | has95 | Aminoacylase-1 | ACY1 | Homo sapiens (Human) | Reviewed(Swiss-Prot) | Hydrolase |
| Q05315 | 1178 | has1178 | Eosinophil lysophospholipase | CLC | Homo sapiens (Human) | Reviewed(Swiss-Prot) | Carbohydrate binding |
| Q05469 | 3991 | has3991 | Hormone-sensitive lipase | LIPE | Homo sapiens (Human) | Reviewed(Swiss-Prot) | Hydrolase |
| Q06203 | 5471 | has5471 | Amidophosphoribosyltransferase | PPAT | Homo sapiens (Human) | Reviewed(Swiss-Prot) | Allosteric enzyme, Glycosyltransfera |
| Q06210 | 2673 | has2673 | Glutamine--fructose-6-phosphate aminotransferase | GFPT1 | Homo sapiens (Human) | Reviewed(Swiss-Prot) | Aminotransferase, Transferase |
| Q07326 | 5281 | has5281 | Phosphatidylinositol-glycan biosynthesis class F pr | PIGF | Homo sapiens (Human) | Reviewed(Swiss-Prot) | Ethanolaminephosphotransferase acti |
| Q08188 | 7053 | has7053 | Protein-glutamine gamma-glutamyltransferase E | TGM3 | Homo sapiens (Human) | Reviewed(Swiss-Prot) | Acyltransferase, Transferase |
| Q12879 | 2903 | has2903 | Glutamate receptor ionotropic, NMDA 2A | GRIN2A | Homo sapiens (Human) | Reviewed(Swiss-Prot) | Ion channel, Ligand-gated ion chann |
| Q13093 | 7941 | has7941 | Platelet-activating factor acetylhydrolase | PLA2G7 | Homo sapiens (Human) | Reviewed(Swiss-Prot) | Hydrolase |
| Q13336 | 6563 | has6563 | Urea transporter 1 | SLC14A1 | Homo sapiens (Human) | Reviewed(Swiss-Prot) | Blood group antigen |
| Q13393 | 5337 | has5337 | Phospholipase D1 | PLD1 | Homo sapiens (Human) | Reviewed(Swiss-Prot) | Hydrolase |
| Q13510 | 427 | has427 | Acid ceramidase | ASAH1 | Homo sapiens (Human) | Reviewed(Swiss-Prot) | Hydrolase |
| Q13724 | 7841 | has7841 | Mannosyl-oligosaccharide glucosidase | MOGS | Homo sapiens (Human) | Reviewed(Swiss-Prot) | Glycosidase, Hydrolase |

| | | | | | | | |
|---|---|---|---|---|---|---|---|
| Q14032 | 570 | has570 | Bile acid-CoA:amino acid N-acyltransferase | BAAT | Homo sapiens (Human) | Reviewed(Swiss-Prot) | Acyltransferase, Hydrolase, Serine es |
| Q14330 | 2841 | has2841 | N-arachidonyl glycine receptor | GPR18 | Homo sapiens (Human) | Reviewed(Swiss-Prot) | G-protein coupled receptor, Receptor |
| Q14397 | 2646 | has2646 | Glucokinase regulatory protein | GCKR | Homo sapiens (Human) | Reviewed(Swiss-Prot) | Fructose-6-phosphate binding |
| Q14442 | 5283 | has5283 | Phosphatidylinositol N-acetylglucosaminyltransfera | PIGH | Homo sapiens (Human) | Reviewed(Swiss-Prot) | Glycosyltransferase, Transferase |
| Q14697 | 23193 | has23193 | Neutral alpha-glucosidase AB | GANAB | Homo sapiens (Human) | Reviewed(Swiss-Prot) | Glycosidase, Hydrolase |
| Q14749 | 27232 | has27232 | Glycine N-methyltransferase | GNMT | Homo sapiens (Human) | Reviewed(Swiss-Prot) | Methyltransferase, Transferase |
| Q14749 | 27232 | has27232 | Glycine N-methyltransferase | GNMT | Homo sapiens (Human) | Reviewed(Swiss-Prot) | Methyltransferase, Transferase |
| Q14957 | 2905 | has2905 | Glutamate receptor ionotropic, NMDA 2C | GRIN2C | Homo sapiens (Human) | Reviewed(Swiss-Prot) | Ion channel, Ligand-gated ion channe |
| Q15102 | 5050 | has5050 | Platelet-activating factor acetylhydrolase IB subuni | PAFAH1B3 | Homo sapiens (Human) | Reviewed(Swiss-Prot) | Hydrolase |
| Q15758 | 6510 | has6510 | Neutral amino acid transporter B(0) | SLC1A5 | Homo sapiens (Human) | Reviewed(Swiss-Prot) | Host cell receptor for virus entry, Re |
| Q15849 | 8170 | has8170 | Urea transporter 2 | SLC14A2 | Homo sapiens (Human) | Reviewed(Swiss-Prot) | Cell adhesion molecule binding |
| Q16719 | 8942 | has8942 | Kynureninase | KYNU | Homo sapiens (Human) | Reviewed(Swiss-Prot) | Hydrolase |
| Q16773 | 883 | has883 | Kynurenine--oxoglutarate transaminase 1 | CCBL1 | Homo sapiens (Human) | Reviewed(Swiss-Prot) | Aminotransferase, Lyase, Transferase |
| Q17RR3 | 119548 | has119548 | Pancreatic lipase-related protein 3 | PNLIPRP3 | Homo sapiens (Human) | Reviewed(Swiss-Prot) | Hydrolase |
| Q2TB90 | 80201 | has80201 | Putative hexokinase HKDC1 | HKDC1 | Homo sapiens (Human) | Reviewed(Swiss-Prot) | Kinase, Transferase |
| Q3MJ16 | 123745 | has123745 | Cytosolic phospholipase A2 epsilon | PLA2G4E | Homo sapiens (Human) | Reviewed(Swiss-Prot) | Hydrolase |
| Q3MUY | 84992 | has84992 | Phosphatidylinositol N-acetylglucosaminyltransfera | PIGY | Homo sapiens (Human) | Reviewed(Swiss-Prot) | |
| Q53ET4 | | | Serine hydroxymethyltransferase | N/A | Homo sapiens (Human) | Reviewed(Swiss-Prot) | Methyltransferase, Transferase |
| Q53EW1 | | | Peroxisome proliferative activated receptor gamma | isoform 2 va | Homo sapiens (Human) | Reviewed(Swiss-Prot) | |
| Q53FV7 | 1375 | has1375 | Carnitine palmitoyltransferase 1B isoform a variant | | Homo sapiens (Human) | Reviewed(Swiss-Prot) | Acyltransferase,Transferase |
| Q53GD3 | 80736 | has80736 | Choline transporter-like protein 4 | SLC44A4 | Homo sapiens (Human) | Reviewed(Swiss-Prot) | Choline transmembrane transporter a |
| Q59EA4 | 5337 | has5337 | Submitted name: Phospholipase D1 variant | N/A | Homo sapiens (Human) | Unreviewed(TrEMBL) | Phosphatidylinositol binding |
| Q5BJF5 | 6472 | has6472 | Serine hydroxymethyltransferase | SHMT2 | Homo sapiens (Human) | Reviewed(Swiss-Prot) | Transferase |
| Q5H8A4 | 54872 | has54872 | GPI ethanolamine phosphate transferase 2 | PIGG | Homo sapiens (Human) | Reviewed(Swiss-Prot) | Transferase |
| Q5HYG8 | 6472 | has6472 | Serine hydroxymethyltransferase | DKFZp686I | Homo sapiens (Human) | Reviewed(Swiss-Prot) | Transferase |
| Q5JTZ9 | 57505 | has57505 | Alanine--tRNA ligase, mitochondrial | AARS2 | Homo sapiens (Human) | Reviewed(Swiss-Prot) | Aminoacyl-tRNA synthetase, Ligase, |
| Q5K4L6 | 11000 | has11000 | Long-chain fatty acid transport protein 3 | SLC27A3 | Homo sapiens (Human) | Reviewed(Swiss-Prot) | Ligase |
| Q5QJU3 | 340485 | has340485 | Alkaline ceramidase 2 | ACER2 | Homo sapiens (Human) | Reviewed(Swiss-Prot) | Hydrolase |
| Q5ST30 | 57176 | has57176 | Valine--tRNA ligase, mitochondrial | VARS2 | Homo sapiens (Human) | Reviewed(Swiss-Prot) | Aminoacyl-tRNA synthetase, Ligase |
| Q5T6X5 | 222545 | has222545 | G-protein coupled receptor family C group 6 memb | GPRC6A | Homo sapiens (Human) | Reviewed(Swiss-Prot) | G-protein coupled receptor, Receptor |
| Q5TA50 | 80772 | has80772 | Glycolipid transfer protein domain-containing prote | GLTPD1 | Homo sapiens (Human) | Reviewed(Swiss-Prot) | Ceramide 1-phosphate binding |
| Q68DD2 | 255189 | has255189 | Cytosolic phospholipase A2 zeta | PLA2G4F | Homo sapiens (Human) | Reviewed(Swiss-Prot) | Hydrolase |
| Q6FHZ7 | 123 | has123 | ADFP protein | ADFP | Homo sapiens (Human) | Reviewed(Swiss-Prot) | |
| Q6IA69 | 55191 | has55191 | Glutamine-dependent NAD(+) synthetase | NADSYN1 | Homo sapiens (Human) | Reviewed(Swiss-Prot) | Ligase |
| Q6IB77 | 10249 | has10249 | Glycine N-acyltransferase | GLYAT | Homo sapiens (Human) | Reviewed(Swiss-Prot) | Acyltransferase, Transferase |
| Q6IBR2 | 2193 | has2193 | Submitted name: FARSLA protein | FARSLA | Homo sapiens (Human) | Reviewed(Swiss-Prot) | Aminoacyl-tRNA synthetase, Ligase |
| Q6L9M1 | | | Peroxisome proliferator-activated receptor | PPARG | Homo sapiens (Human) | Reviewed(Swiss-Prot) | |
| Q6NT32 | 221223 | has221223 | Carboxylesterase 5A | CES5A | Homo sapiens (Human) | Reviewed(Swiss-Prot) | Hydrolase, Serine esterase |
| Q6P1A2 | 10162 | has10162 | Lysophospholipid acyltransferase 5 | LPCAT3 | Homo sapiens (Human) | Reviewed(Swiss-Prot) | Acyltransferase, Transferase |
| Q6P1J6 | 151056 | has151056 | Phospholipase B1, membrane-associated | PLB1 | Homo sapiens (Human) | Reviewed(Swiss-Prot) | Acyltransferase, Transferase |
| Q6P1M0 | 10999 | has10999 | Long-chain fatty acid transport protein 4 | SLC27A4 | Homo sapiens (Human) | Reviewed(Swiss-Prot) | Ligase |
| Q6PCB7 | 376497 | has376497 | Long-chain fatty acid transport protein 1 | SLC27A1 | Homo sapiens (Human) | Reviewed(Swiss-Prot) | Ligase |
| Q6UWW | 23491 | has23491 | Carboxylesterase 3 | CES3 | Homo sapiens (Human) | Reviewed(Swiss-Prot) | Hydrolase, Serine esterase |
| Q6YP21 | 56267 | has56267 | Kynurenine--oxoglutarate transaminase 3 | CCBL2 | Homo sapiens (Human) | Reviewed(Swiss-Prot) | Aminotransferase, Lyase, Transferase |
| Q6ZMR3 | 160287 | has160287 | L-lactate dehydrogenase A-like 6A | LDHAL6A | Homo sapiens (Human) | Reviewed(Swiss-Prot) | Oxidoreductase |
| Q71UV7 | | | Blood group Kidd urea transporter | HUT11 | Homo sapiens (Human) | Reviewed(Swiss-Prot) | Acyltransferase, Transferase |
| Q7L5N7 | 54947 | has54947 | Lysophosphatidylcholine acyltransferase 2 | LPCAT2 | Homo sapiens (Human) | Reviewed(Swiss-Prot) | Acyltransferase, Transferase |
| Q7Z2H8 | 206358 | has206358 | Proton-coupled amino acid transporter 1 | SLC36A1 | Homo sapiens (Human) | Reviewed(Swiss-Prot) | Amino acid:proton symporter activity |

| | | | | | | | |
|---|---|---|---|---|---|---|---|
| Q7Z7B1 | 284098 | has284098 | Phosphatidylinositol-glycan biosynthesis class W p | PIGW | Homo sapiens (Human) | Reviewed(Swiss-Prot) | Acyltransferase, Transferase |
| Q86TX2 | 641371 | has641371 | Acyl-coenzyme A thioesterase 1 | ACOT1 | Homo sapiens (Human) | Reviewed(Swiss-Prot) | Hydrolase, Serine esterase |
| Q86U10 | 374569 | has374569 | 60 kDa lysophospholipase | ASPG | Homo sapiens (Human) | Reviewed(Swiss-Prot) | Hydrolase |
| Q86VD9 | 80235 | has80235 | GPI mannosyltransferase 4 | PIGZ | Homo sapiens (Human) | Reviewed(Swiss-Prot) | Glycosyltransferase, Transferase |
| Q86WD1 | | | CREB3L2-PPARgamma | | Homo sapiens (Human) | Reviewed(Swiss-Prot) | |
| Q86XP0 | 283748 | has283748 | Cytosolic phospholipase A2 delta | PLA2G4D | Homo sapiens (Human) | Reviewed(Swiss-Prot) | Hydrolase |
| Q86Z02 | 204851 | has204851 | Homeodomain-interacting protein kinase 1 | HIPK1 | Homo sapiens (Human) | Reviewed(Swiss-Prot) | Kinase, Serine/threonine-protein kina |
| Q8IUZ5 | 85007 | has85007 | 5-phosphohydroxy-L-lysine phospho-lyase | AGXT2L2 | Homo sapiens (Human) | Reviewed(Swiss-Prot) | Aminotransferase, Lyase, Transferase |
| Q8IV08 | 23646 | has23646 | Phospholipase D3 | PLD3 | Homo sapiens (Human) | Reviewed(Swiss-Prot) | Hydrolase |
| Q8IWA5 | 57153 | has57153 | Choline transporter-like protein 2 | SLC44A2 | Homo sapiens (Human) | Reviewed(Swiss-Prot) | Choline transmembrane transporter a |
| Q8IY17 | 10908 | has10908 | Neuropathy target esterase | PNPLA6 | Homo sapiens (Human) | Reviewed(Swiss-Prot) | Hydrolase |
| Q8N0X4 | 171425 | has171425 | Citramalyl-CoA lyase, mitochondrial | CLYBL | Homo sapiens (Human) | Reviewed(Swiss-Prot) | |
| Q8N2A8 | 201164 | has201164 | Mitochondrial cardiolipin hydrolase | PLD6 | Homo sapiens (Human) | Reviewed(Swiss-Prot) | Endonuclease, Hydrolase, Nuclease |
| Q8N4M1 | 126969 | has126969 | Choline transporter-like protein 3 | SLC44A3 | Homo sapiens (Human) | Reviewed(Swiss-Prot) | Choline transmembrane transporter a |
| Q8N5D6 | 26301 | has26301 | Globoside alpha-1,3-N-acetylgalactosaminyltransfe | GBGT1 | Homo sapiens (Human) | Reviewed(Swiss-Prot) | Glycosyltransferase, Transferase |
| Q8N6M5 | 55821 | has55821 | Probable allantoicase | ALLC | Homo sapiens (Human) | Reviewed(Swiss-Prot) | Hydrolase |
| Q8N9L9 | 122970 | has122970 | Acyl-coenzyme A thioesterase 4 | ACOT4 | Homo sapiens (Human) | Reviewed(Swiss-Prot) | Hydrolase, Serine esterase |
| Q8NCC3 | 23659 | has23659 | Group XV phospholipase A2 | PLA2G15 | Homo sapiens (Human) | Reviewed(Swiss-Prot) | Acyltransferase, Hydrolase, Transfera |
| Q8NE62 | 55349 | has55349 | Choline dehydrogenase, mitochondrial | CHDH | Homo sapiens (Human) | Reviewed(Swiss-Prot) | Oxidoreductase |
| Q8NF37 | 79888 | has79888 | Lysophosphatidylcholine acyltransferase 1 | LPCAT1 | Homo sapiens (Human) | Reviewed(Swiss-Prot) | Acyltransferase, Transferase |
| Q8TBF5 | 54965 | has54965 | Phosphatidylinositol-glycan biosynthesis class X pr | PIGX | Homo sapiens (Human) | Reviewed(Swiss-Prot) | |
| Q8TCT1 | 162466 | has162466 | Phosphoethanolamine/phosphocholine phosphatase | PHOSPHO1 | Homo sapiens (Human) | Reviewed(Swiss-Prot) | Hydrolase |
| Q8TD30 | 84706 | has84706 | Alanine aminotransferase 2 | GPT2 | Homo sapiens (Human) | Reviewed(Swiss-Prot) | Aminotransferase, Transferase |
| Q8TDN7 | 125981 | has125981 | Alkaline ceramidase 1 | ACER1 | Homo sapiens (Human) | Reviewed(Swiss-Prot) | Hydrolase |
| Q8TEQ8 | 84720 | has84720 | GPI ethanolamine phosphate transferase 3 | PIGO | Homo sapiens (Human) | Reviewed(Swiss-Prot) | Transferase |
| Q8TET4 | 2595 | has2595 | Neutral alpha-glucosidase C | GANC | Homo sapiens (Human) | Reviewed(Swiss-Prot) | Glycosidase, Hydrolase |
| Q8TF71 | 117247 | has117247 | Monocarboxylate transporter 10 | SLC16A10 | Homo sapiens (Human) | Reviewed(Swiss-Prot) | Amino acid transmembrane transport |
| Q8WU03 | 219970 | has219970 | Glycine N-acyltransferase-like protein 2 | GLYATL2 | Homo sapiens (Human) | Reviewed(Swiss-Prot) | Acyltransferase, Transferase |
| Q8WWI1 | 23446 | has23446 | Choline transporter-like protein 1 | SLC44A1 | Homo sapiens (Human) | Reviewed(Swiss-Prot) | Choline transmembrane transporter a |
| Q8WXW8 | | | Urea transporter JK glycoprotein | JK | Homo sapiens (Human) | Reviewed(Swiss-Prot) | Urea transmembrane transporter activ |
| Q92521 | 9488 | has9488 | GPI mannosyltransferase 3 | PIGB | Homo sapiens (Human) | Reviewed(Swiss-Prot) | Glycosyltransferase, Transferase |
| Q92535 | 5279 | has5279 | Phosphatidylinositol N-acetylglucosaminyltransfera | PIGC | Homo sapiens (Human) | Reviewed(Swiss-Prot) | Glycosyltransferase, Transferase |
| Q92643 | 10026 | has10026 | GPI-anchor transamidase | PIGK | Homo sapiens (Human) | Reviewed(Swiss-Prot) | Hydrolase, Protease, Thiol protease |
| Q93088 | 635 | has635 | Betaine--homocysteine S-methyltransferase 1 | BHMT | Homo sapiens (Human) | Reviewed(Swiss-Prot) | Methyltransferase, Transferase |
| Q969I3 | 92292 | has92292 | Glycine N-acyltransferase-like protein 1 | GLYATL1 | Homo sapiens (Human) | Reviewed(Swiss-Prot) | Acyltransferase, Transferase |
| Q969N2 | 51604 | has51604 | GPI transamidase component PIG-T | PIGT | Homo sapiens (Human) | Reviewed(Swiss-Prot) | GPI-anchor transamidase activity |
| Q96AD5 | 57104 | has57104 | Patatin-like phospholipase domain-containing prote | PNPLA2 | Homo sapiens (Human) | Reviewed(Swiss-Prot) | Hydrolase |
| Q96BZ4 | 122618 | has122618 | Phospholipase D4 | PLD4 | Homo sapiens (Human) | Reviewed(Swiss-Prot) | Hydrolase |
| Q96DG6 | 134147 | has134147 | Carboxymethylenebutenolidase homolog | CMBL | Homo sapiens (Human) | Reviewed(Swiss-Prot) | Hydrolase |
| Q96EN8 | 55034 | has55034 | Molybdenum cofactor sulfurase | MOCOS | Homo sapiens (Human) | Reviewed(Swiss-Prot) | Molybdenum cofactor sulfurtransfera |
| Q96HD9 | 91703 | has91703 | Aspartoacylase-2 | ACY3 | Homo sapiens (Human) | Reviewed(Swiss-Prot) | Hydrolase |
| Q96I15 | 51540 | has51540 | Selenocysteine lyase | SCLY | Homo sapiens (Human) | Reviewed(Swiss-Prot) | Lyase, Transferase |
| Q96JA3 | 84725 | has84725 | Pleckstrin homology domain-containing family A n | PLEKHA8 | Homo sapiens (Human) | Reviewed(Swiss-Prot) | Ceramide binding |
| Q96KN2 | 84735 | has84735 | Beta-Ala-His dipeptidase | CNDP1 | Homo sapiens (Human) | Reviewed(Swiss-Prot) | Carboxypeptidase activity |
| Q96LB8 | 57115 | has57115 | Peptidoglycan recognition protein 4 | PGLYRP4 | Homo sapiens (Human) | Reviewed(Swiss-Prot) | Antibiotic, Antimicrobial |
| Q96LB9 | 114771 | has114771 | Peptidoglycan recognition protein 3 | PGLYRP3 | Homo sapiens (Human) | Reviewed(Swiss-Prot) | Antibiotic, Antimicrobial |
| Q96PD5 | 114770 | has114770 | N-acetylmuramoyl-L-alanine amidase | PGLYRP2 | Homo sapiens (Human) | Reviewed(Swiss-Prot) | N-acetylmuramoyl-L-alanine amidase |
| Q96PF1 | 116179 | has116179 | Protein-glutamine gamma-glutamyltransferase Z | TGM7 | Homo sapiens (Human) | Reviewed(Swiss-Prot) | Acyltransferase, Transferase |

| | | | | | | | |
|---|---|---|---|---|---|---|---|
| Q96RQ9 | 259307 | has259307 | L-amino-acid oxidase | IL4I1 | Homo sapiens (Human) | Reviewed(Swiss-Prot) | Oxidoreductase |
| Q96RQ9 | 259307 | has259307 | L-amino-acid oxidase | IL4I1 | Homo sapiens (Human) | Reviewed(Swiss-Prot) | Oxidoreductase |
| Q96S52 | 94005 | has94005 | GPI transamidase component PIG-S | PIGS | Homo sapiens (Human) | Reviewed(Swiss-Prot) | GPI-anchor transamidase activity |
| Q99487 | 5051 | has5051 | Platelet-activating factor acetylhydrolase 2, cytopla | PAFAH2 | Homo sapiens (Human) | Reviewed(Swiss-Prot) | Hydrolase |
| Q99518 | 2327 | has2327 | Dimethylaniline monooxygenase [N-oxide-forming | FMO2 | Homo sapiens (Human) | Reviewed(Swiss-Prot) | Monooxygenase, Oxidoreductase |
| Q99624 | 10991 | has10991 | Sodium-coupled neutral amino acid transporter 3 | SLC38A3 | Homo sapiens (Human) | Reviewed(Swiss-Prot) | Amino acid transmembrane transport |
| Q99624 | 10991 | has10991 | Sodium-coupled neutral amino acid transporter 3 | SLC38A3 | Homo sapiens (Human) | Reviewed(Swiss-Prot) | Amino acid transmembrane transport |
| Q99798 | 50 | has50 | Aconitate hydratase, mitochondrial | ACO2 | Homo sapiens (Human) | Reviewed(Swiss-Prot) | |
| Q9BRB3 | 9091 | has9091 | Phosphatidylinositol N-acetylglucosaminyltransfer | PIGQ | Homo sapiens (Human) | Reviewed(Swiss-Prot) | Glycosyltransferase, Transferase |
| Q9BRR6 | 83440 | has83440 | ADP-dependent glucokinase | ADPGK | Homo sapiens (Human) | Reviewed(Swiss-Prot) | Kinase, Transferase |
| Q9BSE5 | 79814 | has79814 | Agmatinase, mitochondrial | AGMAT | Homo sapiens (Human) | Reviewed(Swiss-Prot) | Hydrolase |
| Q9BUM | 92579 | has92579 | Glucose-6-phosphatase 3 | G6PC3 | Homo sapiens (Human) | Reviewed(Swiss-Prot) | Hydrolase |
| Q9BX93 | 84647 | has84647 | Group XIIB secretory phospholipase A2-like protei | PLA2G12B | Homo sapiens (Human) | Reviewed(Swiss-Prot) | Calcium ion binding |
| Q9BY32 | 3704 | has3704 | Inosine triphosphate pyrophosphatase | ITPA | Homo sapiens (Human) | Reviewed(Swiss-Prot) | Hydrolase |
| Q9BYF1 | 59272 | has59272 | Angiotensin-converting enzyme 2 | ACE2 | Homo sapiens (Human) | Reviewed(Swiss-Prot) | Carboxypeptidase, Host cell receptor |
| Q9BYG0 | 84002 | has84002 | Lactosylceramide 1,3-N-acetyl-beta-D-glucosaminy | B3GNT5 | Homo sapiens (Human) | Reviewed(Swiss-Prot) | Developmental protein, Glycosyltran |
| Q9BYV1 | 64902 | has64902 | Alanine--glyoxylate aminotransferase 2, mitochond | AGXT2 | Homo sapiens (Human) | Reviewed(Swiss-Prot) | Aminotransferase, Transferase |
| Q9BYV1 | 64902 | has64902 | Alanine--glyoxylate aminotransferase 2, mitochond | AGXT2 | Homo sapiens (Human) | Reviewed(Swiss-Prot) | Aminotransferase, Transferase |
| Q9BYZ2 | 92483 | has92483 | L-lactate dehydrogenase A-like 6B | LDHAL6B | Homo sapiens (Human) | Reviewed(Swiss-Prot) | Oxidoreductase |
| Q9BZM1 | 81579 | has81579 | Group XIIA secretory phospholipase A2 | PLA2G12A | Homo sapiens (Human) | Reviewed(Swiss-Prot) | Hydrolase |
| Q9BZM2 | 64600 | has64600 | Group IIF secretory phospholipase A2 | PLA2G2F | Homo sapiens (Human) | Reviewed(Swiss-Prot) | Hydrolase |
| Q9BZX2 | 7371 | has7371 | Uridine-cytidine kinase 2 | UCK2 | Homo sapiens (Human) | Reviewed(Swiss-Prot) | Kinase, Transferase |
| Q9GZU7 | 58190 | has58190 | Carboxy-terminal domain RNA polymerase II poly | CTDSP1 | Homo sapiens (Human) | Reviewed(Swiss-Prot) | Hydrolase, Protein phosphatase |
| Q9GZV3 | 60482 | has60482 | High affinity choline transporter 1 | SLC5A7 | Homo sapiens (Human) | Reviewed(Swiss-Prot) | Choline transmembrane transporter a |
| Q9H0R6 | 55278 | has55278 | Glutamyl-tRNA(Gln) amidotransferase subunit A, r | QRSL1 | Homo sapiens (Human) | Reviewed(Swiss-Prot) | Ligase |
| Q9H227 | 57733 | has57733 | Cytosolic beta-glucosidase | GBA3 | Homo sapiens (Human) | Reviewed(Swiss-Prot) | Glycosidase, Hydrolase |
| Q9H3S5 | 93183 | has93183 | GPI mannosyltransferase 1 | PIGM | Homo sapiens (Human) | Reviewed(Swiss-Prot) | Glycosyltransferase, Transferase |
| Q9H490 | 128869 | has128869 | Phosphatidylinositol glycan anchor biosynthesis cla | PIGU | Homo sapiens (Human) | Reviewed(Swiss-Prot) | |
| Q9H598 | 140679 | has140679 | Vesicular inhibitory amino acid transporter | SLC32A1 | Homo sapiens (Human) | Reviewed(Swiss-Prot) | Amino acid transmembrane transport |
| Q9HCG7 | 57704 | has57704 | Non-lysosomal glucosylceramidase | GBA2 | Homo sapiens (Human) | Reviewed(Swiss-Prot) | Glycosidase, Hydrolase |
| Q9NP80 | 50640 | has50640 | Calcium-independent phospholipase A2-gamma | PNPLA8 | Homo sapiens (Human) | Reviewed(Swiss-Prot) | Hydrolase |
| Q9NPB8 | 56261 | has56261 | Glycerophosphocholine phosphodiesterase GPCPD | GPCPD1 | Homo sapiens (Human) | Reviewed(Swiss-Prot) | Hydrolase |
| Q9NQR9 | 57818 | has57818 | Glucose-6-phosphatase 2 | G6PC2 | Homo sapiens (Human) | Reviewed(Swiss-Prot) | Hydrolase |
| Q9NR71 | 56624 | has56624 | Neutral ceramidase | ASAH2 | Homo sapiens (Human) | Reviewed(Swiss-Prot) | Hydrolase |
| Q9NRF8 | 56474 | has56474 | CTP synthase 2 | CTPS2 | Homo sapiens (Human) | Reviewed(Swiss-Prot) | Ligase |
| Q9NSD9 | 10056 | has10056 | Phenylalanine--tRNA ligase beta subunit | FARSB | Homo sapiens (Human) | Reviewed(Swiss-Prot) | Aminoacyl-tRNA synthetase, Ligase |
| Q9NST1 | 80339 | has80339 | Patatin-like phospholipase domain-containing prote | PNPLA3 | Homo sapiens (Human) | Reviewed(Swiss-Prot) | Acyltransferase, Hydrolase, Transfer |
| Q9NUD9 | 55650 | has55650 | GPI mannosyltransferase 2 | PIGV | Homo sapiens (Human) | Reviewed(Swiss-Prot) | Glycosyltransferase, Transferase |
| Q9NV23 | 55301 | has55301 | S-acyl fatty acid synthase thioesterase, medium cha | OLAH | Homo sapiens (Human) | Reviewed(Swiss-Prot) | Hydrolase |
| Q9NZ20 | 50487 | has50487 | Group 3 secretory phospholipase A2 | PLA2G3 | Homo sapiens (Human) | Reviewed(Swiss-Prot) | Hydrolase |
| Q9NZC3 | 51573 | has51573 | Glycerophosphodiester phosphodiesterase 1 | GDE1 | Homo sapiens (Human) | Reviewed(Swiss-Prot) | Hydrolase |
| Q9NZD2 | 51228 | has51228 | Glycolipid transfer protein | GLTP | Homo sapiens (Human) | Reviewed(Swiss-Prot) | Glycolipid binding |
| Q9NZK7 | 30814 | has30814 | Group IIE secretory phospholipase A2 | PLA2G2E | Homo sapiens (Human) | Reviewed(Swiss-Prot) | Hydrolase |
| Q9P0Z9 | 51268 | has51268 | Peroxisomal sarcosine oxidase | PIPOX | Homo sapiens (Human) | Reviewed(Swiss-Prot) | Oxidoreductase |
| Q9UEF6 | | | Peroxisome proliferator activated-receptor gamma | ppar gamma | Homo sapiens (Human) | Unreviewed(TrEMBL) | Receptor |
| Q9UFH6 | | | Putative uncharacterized protein DKFZp434L0435 | DKFZp434l | Homo sapiens (Human) | Unreviewed(TrEMBL) | Receptor |
| Q9UHI5 | 23428 | has23428 | Large neutral amino acids transporter small subunit | SLC7A8 | Homo sapiens (Human) | Reviewed(Swiss-Prot) | Amino acid transmembrane transport |
| Q9UHV8 | 29124 | has29124 | Galactoside-binding soluble lectin 13 | LGALS13 | Homo sapiens (Human) | Reviewed(Swiss-Prot) | Carbohydrate binding |

| | | | | | | | |
|---|---|---|---|---|---|---|---|
| Q9UI32 | 27165 | has27165 | Glutaminase liver isoform, mitochondrial | GLS2 | Homo sapiens (Human) | Reviewed(Swiss-Prot) | Hydrolase |
| Q9UL12 | 1757 | has1757 | Sarcosine dehydrogenase, mitochondrial | SARDH | Homo sapiens (Human) | Reviewed(Swiss-Prot) | Oxidoreductase |
| Q9ULI2 | 57494 | has57494 | Beta-citrylglutamate synthase B | RIMKLB | Homo sapiens (Human) | Reviewed(Swiss-Prot) | Ligase |
| Q9UNK4 | 26279 | has26279 | Group IID secretory phospholipase A2 | PLA2G2D | Homo sapiens (Human) | Reviewed(Swiss-Prot) | Hydrolase |
| Q9UP65 | 8605 | has8605 | Cytosolic phospholipase A2 gamma | PLA2G4C | Homo sapiens (Human) | Reviewed(Swiss-Prot) | Hydrolase |
| Q9Y259 | 1120 | has1120 | Choline/ethanolamine kinase | CHKB | Homo sapiens (Human) | Reviewed(Swiss-Prot) | Kinase, Transferase |
| Q9Y285 | 2193 | has2193 | Phenylalanine--tRNA ligase alpha subunit | FARSA | Homo sapiens (Human) | Reviewed(Swiss-Prot) | Aminoacyl-tRNA synthetase, Ligase |
| Q9Y2B2 | 9487 | has9487 | N-acetylglucosaminyl-phosphatidylinositol de-N-ac | PIGL | Homo sapiens (Human) | Reviewed(Swiss-Prot) | Hydrolase |
| Q9Y2C3 | 10317 | has10317 | Beta-1,3-galactosyltransferase 5 | B3GALT5 | Homo sapiens (Human) | Reviewed(Swiss-Prot) | Glycosyltransferase, Transferase |
| Q9Y2P4 | 28965 | has28965 | Long-chain fatty acid transport protein 6 | SLC27A6 | Homo sapiens (Human) | Reviewed(Swiss-Prot) | Ligase |
| Q9Y345 | 9152 | has9152 | Sodium- and chloride-dependent glycine transporte | SLC6A5 | Homo sapiens (Human) | Reviewed(Swiss-Prot) | Glycine:sodium symporter activity |
| Q9Y5K3 | 9468 | has9468 | Choline-phosphate cytidylyltransferase B | PCYT1B | Homo sapiens (Human) | Reviewed(Swiss-Prot) | Nucleotidyltransferase, Transferase |
| Q9Y5X9 | 9388 | has9388 | Endothelial lipase | LIPG | Homo sapiens (Human) | Reviewed(Swiss-Prot) | Heparin-binding, Hydrolase |
| Q9Y663 | 9955 | has9955 | Heparan sulfate glucosamine 3-O-sulfotransferase 3 | HS3ST3A1 | Homo sapiens (Human) | Reviewed(Swiss-Prot) | Transferase |
| Q9Y697 | 9054 | has9054 | Cysteine desulfurase, mitochondrial | NFS1 | Homo sapiens (Human) | Reviewed(Swiss-Prot) | Transferase |
| Q9Y6K0 | 10390 | has10390 | Choline/ethanolaminephosphotransferase 1 | CEPT1 | Homo sapiens (Human) | Reviewed(Swiss-Prot) | Transferase |

**Supplementary Table S5. The docking score between metabonomic biomarker and target calculate by systemsDock**

| Uniprot ID | PDB | Biomaker | Docking Scores (pKd/pKi) |
|---|---|---|---|
| P04150 | 1P93 | HMDB0004610 | 7.628 |
| P01116 | 1N4R | HMDB0004610 | 7.618 |
| P21359 | 2D4Q | HMDB0004610 | 7.563 |
| P68871 | 1BAB | HMDB0004610 | 7.446 |
| P36551 | 2AEX | HMDB0004610 | 7.41 |
| P14902 | 2D0T | HMDB0004610 | 7.401 |
| P21397 | 2Z5Y | HMDB0010393 | 7.401 |
| P27338 | 1OJA | HMDB0010396 | 7.38 |
| P21397 | 2Z5Y | HMDB0010396 | 7.38 |
| P27338 | 1OJA | HMDB0010393 | 7.376 |
| P28329 | 2FY3 | HMDB0004610 | 7.375 |
| P21397 | 2Z5Y | HMDB0004610 | 7.356 |
| P01308 | 1EVR | HMDB0004610 | 7.353 |
| P51659 | 1IKT | HMDB0004610 | 7.353 |
| P02768 | 1E7A | HMDB0004610 | 7.329 |
| P42345 | 4FAP | HMDB0004610 | 7.324 |
| P04271 | 2H61 | HMDB0004610 | 7.32 |
| P04278 | 1LHV | HMDB0004610 | 7.302 |
| P21802 | 1DJS | HMDB0004610 | 7.292 |
| P21397 | 2Z5Y | HMDB0010384 | 7.283 |
| P02741 | 1CGP | HMDB0004610 | 7.274 |
| Q08499 | 1MKD | HMDB0004610 | 7.274 |
| P01270 | 3C4M | HMDB0004610 | 7.27 |
| P27338 | 1OJA | HMDB0004610 | 7.242 |
| Q30201 | 1DEA | HMDB0004610 | 7.232 |
| Q15067 | 1W07 | HMDB0004610 | 7.209 |
| P12821 | 1UZE | HMDB0004610 | 7.199 |
| Q9Y617 | 3E77 | HMDB0010393 | 7.183 |
| P00441 | 1HL5 | HMDB0004610 | 7.17 |
| Q9Y617 | 3E77 | HMDB0004610 | 7.154 |
| Q99259 | 2OKJ | HMDB0010396 | 7.147 |
| P01375 | 2AZ5 | HMDB0004610 | 7.128 |
| P07949 | 2IVS | HMDB0004610 | 7.124 |
| P28482 | 1PME | HMDB0004610 | 7.115 |
| Q9Y617 | 3E77 | HMDB0010396 | 7.111 |
| Q99259 | 2OKJ | HMDB0010393 | 7.088 |
| P21964 | 3HVH | HMDB0004610 | 7.082 |
| Q9BYW2 | 3H6L | HMDB0004610 | 7.06 |
| P30793 | 1N3T | HMDB0004610 | 7.053 |
| P14416 | 2HLB | HMDB0004610 | 7.049 |
| P42336 | 3HHM | HMDB0004610 | 7.032 |
| Q08499 | 1MKD | HMDB0010393 | 7.023 |
| P16278 | 1BOB | HMDB0004610 | 7.017 |
| P21397 | 2Z5Y | HMDB0010383 | 6.996 |
| P01138 | 1SG1 | HMDB0004610 | 6.988 |
| Q08499 | 1MKD | HMDB0010396 | 6.987 |
| P12821 | 1UZE | HMDB0010396 | 6.979 |
| P17612 | 3MVJ | HMDB0010393 | 6.955 |
| Q9Y617 | 3E77 | HMDB0010384 | 6.947 |
| P08397 | 3ECR | HMDB0004610 | 6.944 |
| P36551 | 2AEX | HMDB0010393 | 6.941 |
| P17612 | 3MVJ | HMDB0010396 | 6.936 |
| P36551 | 2AEX | HMDB0010396 | 6.927 |
| P28329 | 2FY3 | HMDB0010384 | 6.922 |
| P12821 | 1UZE | HMDB0010393 | 6.913 |
| Q15067 | 1W07 | HMDB0010393 | 6.913 |
| P27338 | 1OJA | HMDB0010383 | 6.907 |
| P01116 | 1N4R | HMDB0010393 | 6.903 |
| P36551 | 2AEX | HMDB0010384 | 6.903 |
| P28329 | 2FY3 | HMDB0010393 | 6.901 |
| P28329 | 2FY3 | HMDB0010396 | 6.886 |
| P00441 | 1HL5 | HMDB0010393 | 6.877 |
| P02768 | 1E7A | HMDB0010396 | 6.876 |
| P01116 | 1N4R | HMDB0010396 | 6.876 |
| P00441 | 1HL5 | HMDB0010384 | 6.873 |
| P04278 | 1LHV | HMDB0010396 | 6.873 |

| | | | |
|---|---|---|---|
| Q9BYW2 | 3H6L | HMDB0010396 | 6.862 |
| Q99259 | 2OKJ | HMDB0010384 | 6.861 |
| P01579 | 1EKU | HMDB0010396 | 6.85 |
| P02768 | 1E7A | HMDB0010393 | 6.845 |
| P01138 | 1SG1 | HMDB0010393 | 6.838 |
| P43354 | 1OVL | HMDB0004610 | 6.836 |
| Q8NFD5 | 2EH9 | HMDB0004610 | 6.826 |
| P02741 | 1CGP | HMDB0010393 | 6.824 |
| P04278 | 1LHV | HMDB0010393 | 6.822 |
| Q15067 | 1W07 | HMDB0010396 | 6.818 |
| Q06124 | 2SHP | HMDB0010393 | 6.818 |
| Q06124 | 2SHP | HMDB0010396 | 6.814 |
| P01579 | 1EKU | HMDB0010393 | 6.797 |
| P02741 | 1CGP | HMDB0010396 | 6.789 |
| P01138 | 1SG1 | HMDB0010396 | 6.775 |
| P02741 | 1CGP | HMDB0000099 | 6.767 |
| P00441 | 1HL5 | HMDB0010396 | 6.765 |
| P17612 | 3MVJ | HMDB0010384 | 6.761 |
| P28329 | 2FY3 | HMDB0010383 | 6.756 |
| P42336 | 3HHM | HMDB0010396 | 6.751 |
| Q9BYW2 | 3H6L | HMDB0010393 | 6.749 |
| P01116 | 1N4R | HMDB0010384 | 6.747 |
| P08397 | 3ECR | HMDB0010396 | 6.73 |
| P36551 | 2AEX | HMDB0010383 | 6.725 |
| P12821 | 1UZE | HMDB0010384 | 6.712 |
| P04150 | 1P93 | HMDB0034146 | 6.703 |
| P02768 | 1E7A | HMDB0010384 | 6.702 |
| P21397 | 2Z5Y | HMDB0000883 | 6.702 |
| P02741 | 1CGP | HMDB0010384 | 6.698 |
| P21397 | 2Z5Y | HMDB0000099 | 6.683 |
| P14416 | 2HLB | HMDB0010396 | 6.682 |
| Q08499 | 1MKD | HMDB0010384 | 6.681 |
| P23771 | 3DFV | HMDB0004610 | 6.68 |
| P01270 | 3C4M | HMDB0010396 | 6.679 |
| P01116 | 1N4R | HMDB0034146 | 6.677 |
| P00441 | 1HL5 | HMDB0010383 | 6.668 |
| P27338 | 1OJA | HMDB0000099 | 6.668 |
| P05067 | 2FK3 | HMDB0004610 | 6.666 |
| P14416 | 2HLB | HMDB0010393 | 6.666 |
| P42336 | 3HHM | HMDB0010393 | 6.662 |
| Q06124 | 2SHP | HMDB0010384 | 6.661 |
| P08397 | 3ECR | HMDB0010393 | 6.656 |
| P01308 | 1EVR | HMDB0010393 | 6.648 |
| P62834 | 3KUC | HMDB0010393 | 6.643 |
| P01308 | 1EVR | HMDB0010396 | 6.642 |
| P21802 | 1DJS | HMDB0010393 | 6.641 |
| P21802 | 1DJS | HMDB0010396 | 6.64 |
| P04271 | 2H61 | HMDB0010396 | 6.638 |
| P07949 | 2IVS | HMDB0010383 | 6.634 |
| Q30201 | 1DEA | HMDB0010393 | 6.629 |
| P04150 | 1P93 | HMDB0010384 | 6.628 |
| Q03431 | 3L2J | HMDB0010393 | 6.627 |
| P21359 | 2D4Q | HMDB0010384 | 6.626 |
| P62834 | 3KUC | HMDB0010396 | 6.621 |
| P01138 | 1SG1 | HMDB0010384 | 6.619 |
| P04278 | 1LHV | HMDB0000099 | 6.618 |
| P04150 | 1P93 | HMDB0010393 | 6.618 |
| Q99259 | 2OKJ | HMDB0010383 | 6.616 |
| P10082 | 2ZA5 | HMDB0004610 | 6.613 |
| P12821 | 1UZE | HMDB0000099 | 6.608 |
| P14416 | 2HLB | HMDB0010384 | 6.608 |
| P21359 | 2D4Q | HMDB0010393 | 6.605 |
| P21359 | 2D4Q | HMDB0010383 | 6.603 |
| P21359 | 2D4Q | HMDB0010396 | 6.595 |
| P43354 | 1OVL | HMDB0010384 | 6.593 |
| P04271 | 2H61 | HMDB0010393 | 6.586 |
| P01116 | 1N4R | HMDB0010383 | 6.58 |
| P04150 | 1P93 | HMDB0010396 | 6.579 |
| P27361 | 3FXW | HMDB0010396 | 6.575 |

| | | | |
|---|---|---|---|
| P10082 | 2ZA5 | HMDB0010393 | 6.574 |
| P54098 | 1A1V | HMDB0010396 | 6.57 |
| Q08499 | 1MKD | HMDB0010383 | 6.567 |
| P12821 | 1UZE | HMDB0010383 | 6.566 |
| P54098 | 1A1V | HMDB0010393 | 6.565 |
| P29475 | 1F20 | HMDB0004610 | 6.563 |
| P02768 | 1E7A | HMDB0010383 | 6.556 |
| Q9Y617 | 3E77 | HMDB0010383 | 6.551 |
| P42345 | 4FAP | HMDB0010396 | 6.546 |
| Q15067 | 1W07 | HMDB0010384 | 6.542 |
| P01308 | 1EVR | HMDB0034146 | 6.541 |
| Q9BYW2 | 3H6L | HMDB0010384 | 6.54 |
| Q03431 | 3L2J | HMDB0010396 | 6.538 |
| P30793 | 1N3T | HMDB0010396 | 6.536 |
| P01308 | 1EVR | HMDB0010384 | 6.528 |
| Q30201 | 1DEA | HMDB0000099 | 6.521 |
| P43354 | 1OVL | HMDB0010393 | 6.517 |
| Q8NFD5 | 2EH9 | HMDB0010393 | 6.516 |
| P01112 | 1NVU | HMDB0004610 | 6.515 |
| P14902 | 2D0T | HMDB0010393 | 6.512 |
| P07949 | 2IVS | HMDB0010396 | 6.508 |
| P04278 | 1LHV | HMDB0010384 | 6.503 |
| P01270 | 3C4M | HMDB0010393 | 6.503 |
| P62834 | 3KUC | HMDB0010384 | 6.503 |
| P08069 | 3F5P | HMDB0010384 | 6.494 |
| P42345 | 4FAP | HMDB0010384 | 6.494 |
| P05067 | 2FK3 | HMDB0010393 | 6.488 |
| P15056 | 3II5 | HMDB0010384 | 6.483 |
| P15056 | 3II5 | HMDB0010396 | 6.482 |
| P08069 | 3F5P | HMDB0010393 | 6.481 |
| P01270 | 3C4M | HMDB0010384 | 6.48 |
| P02741 | 1CGP | HMDB0010383 | 6.475 |
| P30793 | 1N3T | HMDB0010393 | 6.469 |
| Q03431 | 3L2J | HMDB0010384 | 6.468 |
| P23771 | 3DFV | HMDB0034146 | 6.46 |
| P16435 | 3FJO | HMDB0004610 | 6.46 |
| P21397 | 2Z5Y | HMDB0034146 | 6.457 |
| P03372 | 1HCQ | HMDB0004610 | 6.456 |
| P01579 | 1EKU | HMDB0010383 | 6.455 |
| P07949 | 2IVS | HMDB0010393 | 6.454 |
| P03372 | 1HCQ | HMDB0034146 | 6.453 |
| Q9Y617 | 3E77 | HMDB0000099 | 6.447 |
| P62834 | 3KUC | HMDB0004610 | 6.444 |
| P04049 | 3CU8 | HMDB0010396 | 6.441 |
| P01308 | 1EVR | HMDB0010383 | 6.44 |
| P27338 | 1OJA | HMDB0034146 | 6.436 |
| Q02750 | 3EQC | HMDB0010396 | 6.436 |
| Q99259 | 2OKJ | HMDB0034146 | 6.435 |
| P51659 | 1IKT | HMDB0010393 | 6.434 |
| P04278 | 1LHV | HMDB0034146 | 6.434 |
| P04049 | 3CU8 | HMDB0010393 | 6.432 |
| P23771 | 3DFV | HMDB0010393 | 6.427 |
| P23771 | 3DFV | HMDB0010396 | 6.427 |
| P05067 | 2FK3 | HMDB0010396 | 6.424 |
| P15056 | 3II5 | HMDB0010393 | 6.423 |
| P42345 | 4FAP | HMDB0010393 | 6.422 |
| P27361 | 3FXW | HMDB0010383 | 6.418 |
| Q08499 | 1MKD | HMDB0000099 | 6.417 |
| P01270 | 3C4M | HMDB0034146 | 6.417 |
| P08069 | 3F5P | HMDB0034146 | 6.415 |
| P17612 | 3MVJ | HMDB0034146 | 6.413 |
| Q30201 | 1DEA | HMDB0010384 | 6.411 |
| P43354 | 1OVL | HMDB0010396 | 6.411 |
| P28482 | 1PME | HMDB0010396 | 6.411 |
| Q15067 | 1W07 | HMDB0034146 | 6.411 |
| P54098 | 1A1V | HMDB0010384 | 6.409 |
| P04049 | 3CU8 | HMDB0010384 | 6.403 |
| P27361 | 3FXW | HMDB0034146 | 6.403 |
| Q02750 | 3EQC | HMDB0010384 | 6.402 |

| | | | |
|---|---|---|---|
| P04150 | 1P93 | HMDB0010383 | 6.399 |
| P27361 | 3FXW | HMDB0010393 | 6.396 |
| P36551 | 2AEX | HMDB0034146 | 6.394 |
| P00441 | 1HL5 | HMDB0034146 | 6.393 |
| P08397 | 3ECR | HMDB0010384 | 6.393 |
| P07949 | 2IVS | HMDB0010384 | 6.39 |
| P21802 | 1DJS | HMDB0034146 | 6.385 |
| P01112 | 1NVU | HMDB0010396 | 6.383 |
| P05067 | 2FK3 | HMDB0010384 | 6.377 |
| P03372 | 1HCQ | HMDB0010396 | 6.375 |
| Q15067 | 1W07 | HMDB0010383 | 6.371 |
| P02768 | 1E7A | HMDB0034146 | 6.369 |
| P78509 | 2DDU | HMDB0010396 | 6.369 |
| P08397 | 3ECR | HMDB0010383 | 6.366 |
| P02787 | 1DAN | HMDB0004610 | 6.365 |
| Q03431 | 3L2J | HMDB0034146 | 6.365 |
| P30793 | 1N3T | HMDB0010384 | 6.364 |
| Q8NFD5 | 2EH9 | HMDB0010396 | 6.364 |
| P14416 | 2HLB | HMDB0010383 | 6.364 |
| P01112 | 1NVU | HMDB0010384 | 6.363 |
| P08069 | 3F5P | HMDB0010396 | 6.362 |
| P28329 | 2FY3 | HMDB0034146 | 6.357 |
| P01112 | 1NVU | HMDB0010393 | 6.355 |
| P01270 | 3C4M | HMDB0010383 | 6.354 |
| P02741 | 1CGP | HMDB0034146 | 6.352 |
| P28482 | 1PME | HMDB0034146 | 6.344 |
| P17612 | 3MVJ | HMDB0010383 | 6.344 |
| Q30201 | 1DEA | HMDB0034146 | 6.342 |
| Q02750 | 3EQC | HMDB0010383 | 6.339 |
| P68871 | 1BAB | HMDB0010393 | 6.338 |
| P27361 | 3FXW | HMDB0010384 | 6.338 |
| P02787 | 1DAN | HMDB0010396 | 6.337 |
| Q03431 | 3L2J | HMDB0010383 | 6.337 |
| P01112 | 1NVU | HMDB0010383 | 6.335 |
| P21964 | 3HVH | HMDB0010396 | 6.333 |
| Q06124 | 2SHP | HMDB0034146 | 6.332 |
| P03372 | 1HCQ | HMDB0010384 | 6.329 |
| P10082 | 2ZA5 | HMDB0010396 | 6.329 |
| Q9BYW2 | 3H6L | HMDB0034146 | 6.329 |
| P51659 | 1IKT | HMDB0010396 | 6.325 |
| Q02750 | 3EQC | HMDB0010393 | 6.325 |
| P28482 | 1PME | HMDB0010393 | 6.324 |
| Q08499 | 1MKD | HMDB0034146 | 6.318 |
| P14416 | 2HLB | HMDB0034146 | 6.316 |
| P02787 | 1DAN | HMDB0010393 | 6.315 |
| P30793 | 1N3T | HMDB0034146 | 6.312 |
| P14902 | 2D0T | HMDB0010396 | 6.312 |
| Q02750 | 3EQC | HMDB0034146 | 6.312 |
| P35520 | 1M54 | HMDB0010393 | 6.307 |
| P10082 | 2ZA5 | HMDB0010384 | 6.302 |
| P21802 | 1DJS | HMDB0010384 | 6.298 |
| P42336 | 3HHM | HMDB0034146 | 6.296 |
| Q9Y617 | 3E77 | HMDB0034146 | 6.293 |
| P51659 | 1IKT | HMDB0034146 | 6.291 |
| P54098 | 1A1V | HMDB0010383 | 6.288 |
| P08397 | 3ECR | HMDB0034146 | 6.287 |
| P68871 | 1BAB | HMDB0034146 | 6.286 |
| P15056 | 3II5 | HMDB0034146 | 6.285 |
| P14902 | 2D0T | HMDB0034146 | 6.283 |
| P23771 | 3DFV | HMDB0010384 | 6.276 |
| P60484 | 1D5R | HMDB0004610 | 6.273 |
| P21802 | 1DJS | HMDB0010383 | 6.27 |
| P35637 | 1G5G | HMDB0010393 | 6.269 |
| P04278 | 1LHV | HMDB0010383 | 6.268 |
| P68871 | 1BAB | HMDB0010396 | 6.265 |
| P21964 | 3HVH | HMDB0034146 | 6.262 |
| P14902 | 2D0T | HMDB0010384 | 6.258 |
| P54098 | 1A1V | HMDB0004610 | 6.257 |
| Q9Y243 | 2X18 | HMDB0004610 | 6.248 |

| | | | |
|---|---|---|---|
| P35637 | 1G5G | HMDB0010396 | 6.247 |
| P03372 | 1HCQ | HMDB0010393 | 6.247 |
| Q30201 | 1DEA | HMDB0010396 | 6.246 |
| P01138 | 1SG1 | HMDB0034146 | 6.246 |
| Q06124 | 2SHP | HMDB0000099 | 6.242 |
| P12821 | 1UZE | HMDB0034146 | 6.229 |
| P11274 | 1K1F | HMDB0004610 | 6.223 |
| Q06124 | 2SHP | HMDB0004610 | 6.223 |
| P21964 | 3HVH | HMDB0010384 | 6.223 |
| P35520 | 1M54 | HMDB0010396 | 6.218 |
| P21964 | 3HVH | HMDB0010393 | 6.217 |
| P35520 | 1M54 | HMDB0034146 | 6.214 |
| P35520 | 1M54 | HMDB0010384 | 6.202 |
| P01589 | 3IU3 | HMDB0010393 | 6.202 |
| Q13255 | 1EWT | HMDB0010396 | 6.197 |
| P01920 | 1UVQ | HMDB0010396 | 6.19 |
| P27487 | 1W1I | HMDB0010393 | 6.189 |
| P04271 | 2H61 | HMDB0010384 | 6.186 |
| P01920 | 1UVQ | HMDB0010384 | 6.185 |
| P01920 | 1UVQ | HMDB0010383 | 6.183 |
| P15289 | 2AIJ | HMDB0010396 | 6.182 |
| P28482 | 1PME | HMDB0010384 | 6.181 |
| P01920 | 1UVQ | HMDB0010393 | 6.181 |
| P01241 | 1AXI | HMDB0010393 | 6.18 |
| P10912 | 1AXI | HMDB0010393 | 6.18 |
| P15056 | 3II5 | HMDB0010383 | 6.18 |
| P15289 | 2AIJ | HMDB0010384 | 6.179 |
| P15289 | 2AIJ | HMDB0010393 | 6.179 |
| P22301 | 2ILK | HMDB0010396 | 6.179 |
| P11362 | 1CVS | HMDB0000294 | 6.175 |
| Q13936 | 1T0J | HMDB0010384 | 6.174 |
| Q13936 | 1T0J | HMDB0010393 | 6.173 |
| P01589 | 3IU3 | HMDB0010396 | 6.169 |
| P51608 | 3C2I | HMDB0010393 | 6.168 |
| Q30201 | 1DEA | HMDB0010383 | 6.167 |
| P51659 | 1IKT | HMDB0010384 | 6.166 |
| P07949 | 2IVS | HMDB0000099 | 6.166 |
| Q13936 | 1T0J | HMDB0010396 | 6.165 |
| P01138 | 1SG1 | HMDB0010383 | 6.163 |
| P08069 | 3F5P | HMDB0010383 | 6.151 |
| Q13255 | 1EWT | HMDB0010384 | 6.146 |
| Q13936 | 1T0J | HMDB0010383 | 6.146 |
| P42336 | 3HHM | HMDB0010384 | 6.144 |
| P15289 | 2AIJ | HMDB0010383 | 6.143 |
| Q16620 | 1HCF | HMDB0000294 | 6.137 |
| P04049 | 3CU8 | HMDB0010383 | 6.137 |
| P35520 | 1M54 | HMDB0004610 | 6.131 |
| P21359 | 2D4Q | HMDB0034146 | 6.131 |
| P04271 | 2H61 | HMDB0034146 | 6.129 |
| Q99700 | 3KTR | HMDB0010396 | 6.128 |
| P54098 | 1A1V | HMDB0034146 | 6.127 |
| Q8NFD5 | 2EH9 | HMDB0010384 | 6.127 |
| P22301 | 2ILK | HMDB0010383 | 6.126 |
| P22301 | 2ILK | HMDB0010393 | 6.126 |
| P22301 | 2ILK | HMDB0010384 | 6.121 |
| P07949 | 2IVS | HMDB0034146 | 6.111 |
| P05019 | 1H02 | HMDB0010396 | 6.109 |
| P68871 | 1BAB | HMDB0010384 | 6.102 |
| P05231 | 1ALU | HMDB0000294 | 6.1 |
| P35520 | 1M54 | HMDB0000294 | 6.095 |
| P23771 | 3DFV | HMDB0010383 | 6.092 |
| P30793 | 1N3T | HMDB0010383 | 6.088 |
| P21333 | 2WFN | HMDB0000294 | 6.085 |
| P29475 | 1F20 | HMDB0000294 | 6.08 |
| P01584 | 2NVH | HMDB0000294 | 6.077 |
| Q9BYW2 | 3H6L | HMDB0010383 | 6.077 |
| P28482 | 1PME | HMDB0010383 | 6.075 |
| P14902 | 2D0T | HMDB0010383 | 6.072 |
| P04637 | 1GZH | HMDB0010396 | 6.062 |

| | | | |
|---|---|---|---|
| P39905 | 2V5E | HMDB0000294 | 6.057 |
| P01589 | 3IU3 | HMDB0010383 | 6.052 |
| P10145 | 1QE6 | HMDB0010396 | 6.051 |
| P43354 | 1OVL | HMDB0010383 | 6.048 |
| P36551 | 2AEX | HMDB0000161 | 6.047 |
| Q15465 | 3M1N | HMDB0010384 | 6.037 |
| P35520 | 1M54 | HMDB0010383 | 6.034 |
| Q06787 | 2QND | HMDB0010393 | 6.033 |
| Q15465 | 3M1N | HMDB0010396 | 6.033 |
| P51659 | 1IKT | HMDB0010383 | 6.03 |
| P05067 | 2FK3 | HMDB0034146 | 6.029 |
| P16278 | 1BOB | HMDB0000294 | 6.019 |
| P01308 | 1EVR | HMDB0000099 | 6.016 |
| P16435 | 3FJO | HMDB0010396 | 6.01 |
| P01589 | 3IU3 | HMDB0010384 | 6.01 |
| Q9Y243 | 2X18 | HMDB0010396 | 6.008 |
| Q99497 | 1PDW | HMDB0000294 | 6.006 |
| P03372 | 1HCQ | HMDB0010383 | 6.003 |
| P04637 | 1GZH | HMDB0000294 | 5.999 |
| Q9Y243 | 2X18 | HMDB0000294 | 5.996 |
| Q9Y243 | 2X18 | HMDB0010393 | 5.992 |
| Q8NER1 | 2NYJ | HMDB0000294 | 5.99 |
| P41159 | 3IIQ | HMDB0000294 | 5.99 |
| P01579 | 1EKU | HMDB0000294 | 5.986 |
| Q9NQC3 | 1OZN | HMDB0010393 | 5.986 |
| P01911 | 2WBJ | HMDB0000294 | 5.986 |
| Q15465 | 3M1N | HMDB0000294 | 5.986 |
| P16278 | 1BOB | HMDB0010393 | 5.982 |
| P27487 | 1W1I | HMDB0000294 | 5.979 |
| Q9Y243 | 2X18 | HMDB0010383 | 5.978 |
| Q9UBP0 | 3B9P | HMDB0010396 | 5.977 |
| P04049 | 3CU8 | HMDB0034146 | 5.977 |
| P51608 | 3C2I | HMDB0010384 | 5.974 |
| Q06787 | 2QND | HMDB0010396 | 5.969 |
| P10145 | 1QE6 | HMDB0010393 | 5.968 |
| P14902 | 2D0T | HMDB0000294 | 5.961 |
| P02741 | 1CGP | HMDB0000161 | 5.96 |
| P10082 | 2ZA5 | HMDB0034146 | 5.96 |
| P15056 | 3II5 | HMDB0000294 | 5.959 |
| P04062 | 2NT0 | HMDB0000294 | 5.956 |
| Q99259 | 2OKJ | HMDB0000161 | 5.953 |
| P78509 | 2DDU | HMDB0010384 | 5.951 |
| P68871 | 1BAB | HMDB0000294 | 5.948 |
| Q13255 | 1EWT | HMDB0010383 | 5.943 |
| P60484 | 1D5R | HMDB0010393 | 5.939 |
| P02741 | 1CGP | HMDB0000294 | 5.938 |
| P60484 | 1D5R | HMDB0010396 | 5.933 |
| O00555 | 3BXK | HMDB0000294 | 5.933 |
| P42336 | 3HHM | HMDB0010383 | 5.933 |
| O43612 | 1UV0 | HMDB0000294 | 5.932 |
| Q06124 | 2SHP | HMDB0000161 | 5.931 |
| P06280 | 3HG3 | HMDB0010393 | 5.931 |
| P16435 | 3FJO | HMDB0010393 | 5.93 |
| P01303 | 1R9N | HMDB0000294 | 5.929 |
| P31749 | 2UZS | HMDB0000294 | 5.928 |
| Q03431 | 3L2J | HMDB0000294 | 5.923 |
| Q9Y2R2 | 2P6X | HMDB0010393 | 5.919 |
| P00450 | 2J5W | HMDB0000294 | 5.918 |
| P09619 | 1GQ5 | HMDB0000294 | 5.914 |
| Q13255 | 1EWT | HMDB0010393 | 5.909 |
| P17612 | 3MVJ | HMDB0000161 | 5.908 |
| Q06124 | 2SHP | HMDB0000294 | 5.907 |
| P21359 | 2D4Q | HMDB0000294 | 5.906 |
| P35637 | 1G5G | HMDB0000294 | 5.905 |
| P42345 | 4FAP | HMDB0034146 | 5.905 |
| P01375 | 2AZ5 | HMDB0034146 | 5.904 |
| P06280 | 3HG3 | HMDB0010396 | 5.904 |
| P05019 | 1H02 | HMDB0010393 | 5.903 |
| Q99259 | 2OKJ | HMDB0000294 | 5.901 |

| | | | |
|---|---|---|---|
| P42345 | 4FAP | HMDB0010383 | 5.899 |
| P43354 | 1OVL | HMDB0000294 | 5.896 |
| P01270 | 3C4M | HMDB0000294 | 5.895 |
| P14416 | 2HLB | HMDB0000294 | 5.891 |
| P29475 | 1F20 | HMDB0010393 | 5.89 |
| P01241 | 1AXI | HMDB0010396 | 5.885 |
| P10912 | 1AXI | HMDB0010396 | 5.885 |
| P27487 | 1W1I | HMDB0010396 | 5.884 |
| Q9Y2R2 | 2P6X | HMDB0000294 | 5.883 |
| P08833 | 1ZT3 | HMDB0000294 | 5.882 |
| Q15067 | 1W07 | HMDB0000294 | 5.879 |
| P04278 | 1LHV | HMDB0000294 | 5.878 |
| P01241 | 1AXI | HMDB0010384 | 5.876 |
| P10912 | 1AXI | HMDB0010384 | 5.876 |
| P16278 | 1BOB | HMDB0010384 | 5.876 |
| P29475 | 1F20 | HMDB0010396 | 5.873 |
| P62834 | 3KUC | HMDB0000294 | 5.873 |
| P02787 | 1DAN | HMDB0034146 | 5.871 |
| P26358 | 3EPZ | HMDB0000294 | 5.871 |
| Q9UBK2 | 1XB7 | HMDB0000294 | 5.869 |
| P01375 | 2AZ5 | HMDB0010396 | 5.869 |
| P04637 | 1GZH | HMDB0010393 | 5.868 |
| Q8NFD5 | 2EH9 | HMDB0000294 | 5.868 |
| P42858 | 3IO4 | HMDB0000294 | 5.867 |
| Q9NQC3 | 1OZN | HMDB0000294 | 5.864 |
| P12821 | 1UZE | HMDB0000294 | 5.864 |
| Q9UBP0 | 3B9P | HMDB0010393 | 5.863 |
| Q9UBP0 | 3B9P | HMDB0000294 | 5.862 |
| P07949 | 2IVS | HMDB0000294 | 5.859 |
| P00441 | 1HL5 | HMDB0000099 | 5.858 |
| P36551 | 2AEX | HMDB0000294 | 5.858 |
| P30793 | 1N3T | HMDB0000099 | 5.857 |
| P04271 | 2H61 | HMDB0000294 | 5.854 |
| P06850 | 1MO1 | HMDB0010393 | 5.853 |
| P42858 | 3IO4 | HMDB0010393 | 5.851 |
| P51659 | 1IKT | HMDB0000294 | 5.846 |
| P10145 | 1QE6 | HMDB0010383 | 5.84 |
| P04049 | 3CU8 | HMDB0000294 | 5.84 |
| P68871 | 1BAB | HMDB0010383 | 5.838 |
| P04637 | 1GZH | HMDB0010384 | 5.837 |
| P08397 | 3ECR | HMDB0000294 | 5.837 |
| P06280 | 3HG3 | HMDB0010384 | 5.836 |
| Q14203 | 2HL5 | HMDB0010383 | 5.834 |
| P27986 | 1H9O | HMDB0000294 | 5.833 |
| P01270 | 3C4M | HMDB0000099 | 5.833 |
| P21802 | 1DJS | HMDB0000294 | 5.829 |
| P10082 | 2ZA5 | HMDB0010383 | 5.829 |
| P35637 | 1G5G | HMDB0010383 | 5.824 |
| Q15465 | 3M1N | HMDB0010393 | 5.824 |
| P78504 | 2VJ2 | HMDB0010396 | 5.821 |
| P10082 | 2ZA5 | HMDB0000294 | 5.821 |
| P01112 | 1NVU | HMDB0000294 | 5.82 |
| P06280 | 3HG3 | HMDB0010383 | 5.819 |
| P49917 | 3II6 | HMDB0004610 | 5.818 |
| P01112 | 1NVU | HMDB0034146 | 5.815 |
| Q9Y2R2 | 2P6X | HMDB0010383 | 5.814 |
| Q9Y243 | 2X18 | HMDB0010384 | 5.812 |
| Q7LG56 | 3HF1 | HMDB0000294 | 5.809 |
| Q15067 | 1W07 | HMDB0000161 | 5.808 |
| P06280 | 3HG3 | HMDB0000294 | 5.808 |
| P11274 | 1K1F | HMDB0000294 | 5.807 |
| P29475 | 1F20 | HMDB0010384 | 5.805 |
| P30793 | 1N3T | HMDB0000294 | 5.804 |
| P29475 | 1F20 | HMDB0010383 | 5.8 |
| P17612 | 3MVJ | HMDB0000294 | 5.8 |
| P16278 | 1BOB | HMDB0010396 | 5.797 |
| P28482 | 1PME | HMDB0000294 | 5.794 |
| P42345 | 4FAP | HMDB0000294 | 5.793 |
| Q15303 | 2AHX | HMDB0010384 | 5.792 |

| | | | |
|---|---|---|---|
| P06280 | 3HG3 | HMDB0004610 | 5.79 |
| P04271 | 2H61 | HMDB0010383 | 5.784 |
| P01584 | 2NVH | HMDB0010384 | 5.784 |
| P54098 | 1A1V | HMDB0000294 | 5.783 |
| P29475 | 1F20 | HMDB0034146 | 5.776 |
| P01375 | 2AZ5 | HMDB0010393 | 5.776 |
| P11274 | 1K1F | HMDB0010383 | 5.773 |
| P16435 | 3FJO | HMDB0010384 | 5.772 |
| Q30201 | 1DEA | HMDB0000294 | 5.771 |
| P27986 | 1H9O | HMDB0010383 | 5.771 |
| P60568 | 1PY2 | HMDB0000294 | 5.769 |
| P06850 | 1MO1 | HMDB0010383 | 5.764 |
| P26358 | 3EPZ | HMDB0010383 | 5.762 |
| P10145 | 1QE6 | HMDB0000294 | 5.761 |
| P27338 | 1OJA | HMDB0000159 | 5.758 |
| Q14203 | 2HL5 | HMDB0010396 | 5.758 |
| P21397 | 2Z5Y | HMDB0000294 | 5.758 |
| Q06787 | 2QND | HMDB0000294 | 5.757 |
| P05019 | 1H02 | HMDB0010383 | 5.756 |
| Q99700 | 3KTR | HMDB0010384 | 5.752 |
| P43354 | 1OVL | HMDB0034146 | 5.751 |
| Q9Y2R2 | 2P6X | HMDB0010396 | 5.751 |
| P62834 | 3KUC | HMDB0010383 | 5.749 |
| P01375 | 2AZ5 | HMDB0000294 | 5.746 |
| Q15303 | 2AHX | HMDB0010393 | 5.743 |
| P42336 | 3HHM | HMDB0000294 | 5.743 |
| P10145 | 1QE6 | HMDB0010384 | 5.742 |
| P11274 | 1K1F | HMDB0010396 | 5.739 |
| P05019 | 1H02 | HMDB0010384 | 5.738 |
| Q9BYW2 | 3H6L | HMDB0000294 | 5.733 |
| P51608 | 3C2I | HMDB0010396 | 5.732 |
| P55072 | 1Y8E | HMDB0010383 | 5.731 |
| P04062 | 2NT0 | HMDB0010396 | 5.731 |
| P60568 | 1PY2 | HMDB0010393 | 5.725 |
| P62834 | 3KUC | HMDB0034146 | 5.723 |
| Q08499 | 1MKD | HMDB0000294 | 5.717 |
| Q8NFD5 | 2EH9 | HMDB0010383 | 5.716 |
| P16435 | 3FJO | HMDB0034146 | 5.716 |
| Q99497 | 1PDW | HMDB0004610 | 5.714 |
| P16435 | 3FJO | HMDB0010383 | 5.714 |
| P20783 | 1BND | HMDB0010383 | 5.713 |
| P23560 | 1BND | HMDB0010383 | 5.713 |
| P42858 | 3IO4 | HMDB0010383 | 5.712 |
| P16278 | 1BOB | HMDB0010383 | 5.711 |
| P22301 | 2ILK | HMDB0000161 | 5.711 |
| Q9UBK2 | 1XB7 | HMDB0010383 | 5.71 |
| P22301 | 2ILK | HMDB0002329 | 5.707 |
| P01116 | 1N4R | HMDB0000294 | 5.705 |
| Q9Y2R2 | 2P6X | HMDB0010384 | 5.705 |
| P04637 | 1GZH | HMDB0010383 | 5.704 |
| P55072 | 1Y8E | HMDB0010393 | 5.704 |
| P11274 | 1K1F | HMDB0010393 | 5.701 |
| P42858 | 3IO4 | HMDB0010384 | 5.698 |
| P27986 | 1H9O | HMDB0010384 | 5.697 |
| P14416 | 2HLB | HMDB0000161 | 5.697 |
| P01584 | 2NVH | HMDB0010383 | 5.697 |
| P60484 | 1D5R | HMDB0000294 | 5.694 |
| P22301 | 2ILK | HMDB0000190 | 5.694 |
| P16435 | 3FJO | HMDB0000294 | 5.691 |
| P21964 | 3HVH | HMDB0000294 | 5.689 |
| P01127 | 3MJG | HMDB0010393 | 5.689 |
| P21802 | 1DJS | HMDB0000161 | 5.688 |
| P06850 | 1MO1 | HMDB0010396 | 5.686 |
| P01127 | 3MJG | HMDB0010396 | 5.684 |
| P21397 | 2Z5Y | HMDB0000161 | 5.683 |
| P00441 | 1HL5 | HMDB0000294 | 5.679 |
| O15146 | 2IEP | HMDB0010384 | 5.679 |
| P01308 | 1EVR | HMDB0000294 | 5.678 |
| P01584 | 2NVH | HMDB0010396 | 5.678 |

| | | | |
|---|---|---|---|
| P49917 | 3II6 | HMDB0010396 | 5.677 |
| Q13936 | 1T0J | HMDB0000161 | 5.676 |
| Q15303 | 2AHX | HMDB0010396 | 5.675 |
| P02768 | 1E7A | HMDB0000294 | 5.672 |
| P21397 | 2Z5Y | HMDB0000159 | 5.672 |
| P51608 | 3C2I | HMDB0010383 | 5.671 |
| P01127 | 3MJG | HMDB0010383 | 5.671 |
| P26358 | 3EPZ | HMDB0010393 | 5.666 |
| Q99700 | 3KTR | HMDB0010393 | 5.666 |
| P01127 | 3MJG | HMDB0000294 | 5.666 |
| P01589 | 3IU3 | HMDB0000161 | 5.664 |
| P01920 | 1UVQ | HMDB0000161 | 5.663 |
| P35637 | 1G5G | HMDB0010384 | 5.661 |
| Q13936 | 1T0J | HMDB0000190 | 5.661 |
| Q9Y617 | 3E77 | HMDB0000294 | 5.66 |
| P08833 | 1ZT3 | HMDB0010396 | 5.658 |
| P78504 | 2VJ2 | HMDB0010393 | 5.658 |
| P55072 | 1Y8E | HMDB0010384 | 5.657 |
| P01589 | 3IU3 | HMDB0000294 | 5.657 |
| Q5S007 | 3D6T | HMDB0000294 | 5.656 |
| P27361 | 3FXW | HMDB0000294 | 5.656 |
| P36551 | 2AEX | HMDB0000123 | 5.654 |
| Q13255 | 1EWT | HMDB0000161 | 5.652 |
| P27487 | 1W1I | HMDB0000161 | 5.652 |
| P42858 | 3IO4 | HMDB0010396 | 5.652 |
| P35520 | 1M54 | HMDB0000161 | 5.651 |
| O43612 | 1UV0 | HMDB0010393 | 5.651 |
| P15289 | 2AIJ | HMDB0000161 | 5.651 |
| P27986 | 1H9O | HMDB0010396 | 5.65 |
| P16278 | 1BOB | HMDB0034146 | 5.645 |
| P42858 | 3IO4 | HMDB0000161 | 5.645 |
| P20783 | 1BND | HMDB0010384 | 5.644 |
| P23560 | 1BND | HMDB0010384 | 5.644 |
| P51608 | 3C2I | HMDB0000161 | 5.643 |
| P03372 | 1HCQ | HMDB0000294 | 5.639 |
| P30793 | 1N3T | HMDB0000161 | 5.637 |
| P26358 | 3EPZ | HMDB0004610 | 5.637 |
| Q15303 | 2AHX | HMDB0010383 | 5.636 |
| P01911 | 2WBJ | HMDB0010383 | 5.635 |
| P11274 | 1K1F | HMDB0010384 | 5.633 |
| P60568 | 1PY2 | HMDB0010384 | 5.631 |
| O15146 | 2IEP | HMDB0010383 | 5.629 |
| P27487 | 1W1I | HMDB0010384 | 5.628 |
| P26358 | 3EPZ | HMDB0010396 | 5.628 |
| P06850 | 1MO1 | HMDB0010384 | 5.627 |
| P55072 | 1Y8E | HMDB0010396 | 5.625 |
| P01584 | 2NVH | HMDB0010393 | 5.623 |
| Q9UBP0 | 3B9P | HMDB0004610 | 5.621 |
| Q9UBK2 | 1XB7 | HMDB0010396 | 5.618 |
| Q99497 | 1PDW | HMDB0010393 | 5.617 |
| O43612 | 1UV0 | HMDB0010384 | 5.617 |
| P49917 | 3II6 | HMDB0000294 | 5.617 |
| P05067 | 2FK3 | HMDB0010383 | 5.616 |
| P60484 | 1D5R | HMDB0034146 | 5.613 |
| Q7LG56 | 3HF1 | HMDB0010383 | 5.612 |
| Q99259 | 2OKJ | HMDB0000123 | 5.608 |
| Q99700 | 3KTR | HMDB0000294 | 5.608 |
| P01127 | 3MJG | HMDB0010384 | 5.608 |
| Q9Y617 | 3E77 | HMDB0000123 | 5.606 |
| Q06787 | 2QND | HMDB0010383 | 5.606 |
| Q16620 | 1HCF | HMDB0010393 | 5.605 |
| P04150 | 1P93 | HMDB0000294 | 5.603 |
| P26358 | 3EPZ | HMDB0010384 | 5.602 |
| Q7LG56 | 3HF1 | HMDB0010384 | 5.602 |
| P04062 | 2NT0 | HMDB0010393 | 5.601 |
| P08397 | 3ECR | HMDB0000099 | 5.6 |
| P04062 | 2NT0 | HMDB0010383 | 5.599 |
| Q06787 | 2QND | HMDB0010384 | 5.599 |
| O43612 | 1UV0 | HMDB0010383 | 5.597 |

| | | | |
|---|---|---|---|
| Q99700 | 3KTR | HMDB0010383 | 5.597 |
| P60568 | 1PY2 | HMDB0010383 | 5.595 |
| P08397 | 3ECR | HMDB0000161 | 5.595 |
| P49917 | 3II6 | HMDB0010384 | 5.595 |
| P51659 | 1IKT | HMDB0000099 | 5.593 |
| O15146 | 2IEP | HMDB0000294 | 5.593 |
| P00450 | 2J5W | HMDB0010384 | 5.591 |
| P39905 | 2V5E | HMDB0010384 | 5.59 |
| Q99497 | 1PDW | HMDB0034146 | 5.588 |
| Q02750 | 3EQC | HMDB0000294 | 5.588 |
| Q16620 | 1HCF | HMDB0010384 | 5.587 |
| Q15465 | 3M1N | HMDB0010383 | 5.586 |
| P27338 | 1OJA | HMDB0000123 | 5.585 |
| P00450 | 2J5W | HMDB0010393 | 5.585 |
| P04278 | 1LHV | HMDB0000161 | 5.582 |
| Q13936 | 1T0J | HMDB0001494 | 5.581 |
| P49917 | 3II6 | HMDB0010383 | 5.579 |
| P08069 | 3F5P | HMDB0000294 | 5.577 |
| P04637 | 1GZH | HMDB0004610 | 5.576 |
| P78509 | 2DDU | HMDB0004610 | 5.576 |
| P28329 | 2FY3 | HMDB0000294 | 5.575 |
| P78504 | 2VJ2 | HMDB0010384 | 5.574 |
| Q30201 | 1DEA | HMDB0000161 | 5.573 |
| P02741 | 1CGP | HMDB0000123 | 5.572 |
| P09619 | 1GQ5 | HMDB0010396 | 5.572 |
| P20783 | 1BND | HMDB0000294 | 5.571 |
| P23560 | 1BND | HMDB0000294 | 5.571 |
| P27986 | 1H9O | HMDB0010393 | 5.571 |
| P16220 | 1DH3 | HMDB0000294 | 5.57 |
| P17612 | 3MVJ | HMDB0000123 | 5.568 |
| P78509 | 2DDU | HMDB0000294 | 5.566 |
| P49917 | 3II6 | HMDB0010393 | 5.565 |
| P02787 | 1DAN | HMDB0000294 | 5.562 |
| P20783 | 1BND | HMDB0010396 | 5.56 |
| P23560 | 1BND | HMDB0010396 | 5.56 |
| P01911 | 2WBJ | HMDB0010384 | 5.558 |
| P01241 | 1AXI | HMDB0010383 | 5.557 |
| P10912 | 1AXI | HMDB0010383 | 5.557 |
| P07949 | 2IVS | HMDB0000161 | 5.557 |
| Q06124 | 2SHP | HMDB0000123 | 5.557 |
| Q9UBP0 | 3B9P | HMDB0010384 | 5.557 |
| Q8NER1 | 2NYJ | HMDB0010383 | 5.556 |
| P12821 | 1UZE | HMDB0000161 | 5.555 |
| Q15303 | 2AHX | HMDB0000294 | 5.552 |
| Q7LG56 | 3HF1 | HMDB0010393 | 5.552 |
| P27487 | 1W1I | HMDB0010383 | 5.55 |
| P02787 | 1DAN | HMDB0010384 | 5.548 |
| Q15067 | 1W07 | HMDB0000123 | 5.541 |
| Q5S007 | 3D6T | HMDB0000190 | 5.537 |
| P05019 | 1H02 | HMDB0000294 | 5.534 |
| P14902 | 2D0T | HMDB0000161 | 5.534 |
| Q9UBK2 | 1XB7 | HMDB0000161 | 5.533 |
| Q7LG56 | 3HF1 | HMDB0010396 | 5.533 |
| P05067 | 2FK3 | HMDB0000294 | 5.531 |
| Q9UBK2 | 1XB7 | HMDB0010384 | 5.53 |
| Q99497 | 1PDW | HMDB0010383 | 5.527 |
| Q9UBP0 | 3B9P | HMDB0010383 | 5.523 |
| P51608 | 3C2I | HMDB0000294 | 5.523 |
| Q8NFD5 | 2EH9 | HMDB0034146 | 5.521 |
| Q08499 | 1MKD | HMDB0000925 | 5.52 |
| Q14203 | 2HL5 | HMDB0010393 | 5.517 |
| Q30201 | 1DEA | HMDB0000123 | 5.516 |
| Q08499 | 1MKD | HMDB0000123 | 5.513 |
| Q8NER1 | 2NYJ | HMDB0010396 | 5.513 |
| Q5S007 | 3D6T | HMDB0010396 | 5.511 |
| P01579 | 1EKU | HMDB0010384 | 5.507 |
| Q9UBK2 | 1XB7 | HMDB0010393 | 5.503 |
| Q99700 | 3KTR | HMDB0000161 | 5.503 |
| Q99497 | 1PDW | HMDB0010396 | 5.5 |

| | | | |
|---|---|---|---|
| P27986 | 1H9O | HMDB0004610 | 5.496 |
| P12821 | 1UZE | HMDB0000123 | 5.496 |
| Q5S007 | 3D6T | HMDB0010383 | 5.495 |
| P04062 | 2NT0 | HMDB0010384 | 5.494 |
| Q16620 | 1HCF | HMDB0010383 | 5.493 |
| O43612 | 1UV0 | HMDB0010396 | 5.493 |
| P78509 | 2DDU | HMDB0010383 | 5.493 |
| P21333 | 2WFN | HMDB0010393 | 5.493 |
| P11362 | 1CVS | HMDB0010383 | 5.492 |
| P23771 | 3DFV | HMDB0000294 | 5.49 |
| P20783 | 1BND | HMDB0010393 | 5.489 |
| P23560 | 1BND | HMDB0010393 | 5.489 |
| Q9NQC3 | 1OZN | HMDB0010383 | 5.488 |
| Q9Y617 | 3E77 | HMDB0000161 | 5.487 |
| P08833 | 1ZT3 | HMDB0010384 | 5.487 |
| Q8NER1 | 2NYJ | HMDB0010393 | 5.487 |
| Q03431 | 3L2J | HMDB0000161 | 5.487 |
| P08397 | 3ECR | HMDB0000123 | 5.486 |
| P21802 | 1DJS | HMDB0000123 | 5.483 |
| P31749 | 2UZS | HMDB0010393 | 5.48 |
| Q9NQC3 | 1OZN | HMDB0010384 | 5.477 |
| P30793 | 1N3T | HMDB0000925 | 5.475 |
| Q16620 | 1HCF | HMDB0010396 | 5.474 |
| Q9NQC3 | 1OZN | HMDB0010396 | 5.473 |
| P03372 | 1HCQ | HMDB0000099 | 5.471 |
| P08833 | 1ZT3 | HMDB0010393 | 5.471 |
| Q5S007 | 3D6T | HMDB0010393 | 5.47 |
| P68871 | 1BAB | HMDB0000122 | 5.469 |
| P27338 | 1OJA | HMDB0000925 | 5.467 |
| P01138 | 1SG1 | HMDB0000122 | 5.467 |
| O15146 | 2IEP | HMDB0004610 | 5.467 |
| P01270 | 3C4M | HMDB0000122 | 5.466 |
| Q16620 | 1HCF | HMDB0004610 | 5.465 |
| P14902 | 2D0T | HMDB0000122 | 5.465 |
| P78509 | 2DDU | HMDB0034146 | 5.465 |
| P01270 | 3C4M | HMDB0000161 | 5.465 |
| P51659 | 1IKT | HMDB0000122 | 5.464 |
| P10145 | 1QE6 | HMDB0004610 | 5.462 |
| Q03431 | 3L2J | HMDB0000123 | 5.461 |
| P14416 | 2HLB | HMDB0000123 | 5.46 |
| Q99700 | 3KTR | HMDB0000925 | 5.458 |
| O15146 | 2IEP | HMDB0010396 | 5.457 |
| Q5S007 | 3D6T | HMDB0010384 | 5.457 |
| P01241 | 1AXI | HMDB0000294 | 5.456 |
| P10912 | 1AXI | HMDB0000294 | 5.456 |
| P21964 | 3HVH | HMDB0000122 | 5.453 |
| P15056 | 3II5 | HMDB0000122 | 5.453 |
| P22301 | 2ILK | HMDB0000123 | 5.452 |
| P04278 | 1LHV | HMDB0000123 | 5.451 |
| Q8NER1 | 2NYJ | HMDB0010384 | 5.451 |
| Q03431 | 3L2J | HMDB0000122 | 5.45 |
| P09619 | 1GQ5 | HMDB0010393 | 5.449 |
| P01375 | 2AZ5 | HMDB0010383 | 5.447 |
| O15146 | 2IEP | HMDB0010393 | 5.447 |
| P01127 | 3MJG | HMDB0000925 | 5.447 |
| P36551 | 2AEX | HMDB0000122 | 5.446 |
| P14416 | 2HLB | HMDB0000925 | 5.445 |
| P04278 | 1LHV | HMDB0000122 | 5.444 |
| P17612 | 3MVJ | HMDB0000122 | 5.443 |
| Q16620 | 1HCF | HMDB0034146 | 5.442 |
| P05231 | 1ALU | HMDB0010384 | 5.441 |
| P11362 | 1CVS | HMDB0010384 | 5.439 |
| P31749 | 2UZS | HMDB0010384 | 5.439 |
| Q15067 | 1W07 | HMDB0000122 | 5.438 |
| P04062 | 2NT0 | HMDB0004610 | 5.438 |
| Q30201 | 1DEA | HMDB0000122 | 5.436 |
| P27361 | 3FXW | HMDB0000122 | 5.436 |
| P42336 | 3HHM | HMDB0000099 | 5.435 |
| P41159 | 3IIQ | HMDB0010393 | 5.435 |

| | | | |
|---|---|---|---|
| P05019 | 1H02 | HMDB0000925 | 5.434 |
| O00555 | 3BXK | HMDB0010393 | 5.434 |
| P15056 | 3II5 | HMDB0000099 | 5.434 |
| P28482 | 1PME | HMDB0000122 | 5.432 |
| P21802 | 1DJS | HMDB0000122 | 5.431 |
| Q99259 | 2OKJ | HMDB0000122 | 5.43 |
| P39905 | 2V5E | HMDB0010383 | 5.43 |
| P30793 | 1N3T | HMDB0000122 | 5.428 |
| P08833 | 1ZT3 | HMDB0010383 | 5.428 |
| P30793 | 1N3T | HMDB0000123 | 5.427 |
| P09619 | 1GQ5 | HMDB0004610 | 5.426 |
| P05231 | 1ALU | HMDB0010383 | 5.425 |
| P55072 | 1Y8E | HMDB0000161 | 5.425 |
| P14416 | 2HLB | HMDB0000122 | 5.425 |
| Q14203 | 2HL5 | HMDB0010384 | 5.424 |
| P04150 | 1P93 | HMDB0000122 | 5.423 |
| P01303 | 1R9N | HMDB0010384 | 5.423 |
| P39905 | 2V5E | HMDB0010393 | 5.423 |
| P41159 | 3IIQ | HMDB0010396 | 5.422 |
| P02787 | 1DAN | HMDB0010383 | 5.421 |
| P22301 | 2ILK | HMDB0000925 | 5.421 |
| Q9Y2R2 | 2P6X | HMDB0034146 | 5.421 |
| O00555 | 3BXK | HMDB0010396 | 5.421 |
| P01116 | 1N4R | HMDB0000122 | 5.42 |
| P21333 | 2WFN | HMDB0010383 | 5.419 |
| Q9BYW2 | 3H6L | HMDB0000122 | 5.419 |
| Q99497 | 1PDW | HMDB0010384 | 5.418 |
| P39905 | 2V5E | HMDB0001494 | 5.416 |
| P21333 | 2WFN | HMDB0034146 | 5.416 |
| Q06787 | 2QND | HMDB0004610 | 5.414 |
| P60568 | 1PY2 | HMDB0000925 | 5.414 |
| P51608 | 3C2I | HMDB0000925 | 5.413 |
| P26358 | 3EPZ | HMDB0001494 | 5.413 |
| P22301 | 2ILK | HMDB0001494 | 5.411 |
| P20783 | 1BND | HMDB0000925 | 5.41 |
| P23560 | 1BND | HMDB0000925 | 5.41 |
| P01584 | 2NVH | HMDB0000925 | 5.41 |
| P78504 | 2VJ2 | HMDB0010383 | 5.41 |
| P21333 | 2WFN | HMDB0004610 | 5.408 |
| Q13255 | 1EWT | HMDB0000294 | 5.407 |
| P04271 | 2H61 | HMDB0000122 | 5.407 |
| P01241 | 1AXI | HMDB0000925 | 5.406 |
| P10912 | 1AXI | HMDB0000925 | 5.406 |
| P02768 | 1E7A | HMDB0000122 | 5.405 |
| O00555 | 3BXK | HMDB0000925 | 5.405 |
| Q13936 | 1T0J | HMDB0000123 | 5.401 |
| Q9UBK2 | 1XB7 | HMDB0004610 | 5.4 |
| P00450 | 2J5W | HMDB0010383 | 5.4 |
| P00450 | 2J5W | HMDB0010396 | 5.4 |
| O00555 | 3BXK | HMDB0010384 | 5.4 |
| P01303 | 1R9N | HMDB0010393 | 5.399 |
| P39905 | 2V5E | HMDB0010396 | 5.399 |
| Q06124 | 2SHP | HMDB0000122 | 5.398 |
| P16278 | 1BOB | HMDB0001494 | 5.397 |
| Q06787 | 2QND | HMDB0001494 | 5.397 |
| P35520 | 1M54 | HMDB0000925 | 5.394 |
| P11362 | 1CVS | HMDB0001494 | 5.393 |
| Q5S007 | 3D6T | HMDB0000925 | 5.393 |
| P01920 | 1UVQ | HMDB0000123 | 5.392 |
| P01270 | 3C4M | HMDB0000123 | 5.392 |
| P06850 | 1MO1 | HMDB0000925 | 5.391 |
| P04062 | 2NT0 | HMDB0000925 | 5.391 |
| P08397 | 3ECR | HMDB0000122 | 5.391 |
| P05231 | 1ALU | HMDB0000925 | 5.389 |
| P09619 | 1GQ5 | HMDB0010383 | 5.389 |
| P01589 | 3IU3 | HMDB0000123 | 5.389 |
| P35637 | 1G5G | HMDB0000925 | 5.388 |
| P43354 | 1OVL | HMDB0001494 | 5.388 |
| O43612 | 1UV0 | HMDB0000925 | 5.388 |

| | | | |
|---|---|---|---|
| Q9UBK2 | 1XB7 | HMDB0000925 | 5.388 |
| P27338 | 1OJA | HMDB0000122 | 5.387 |
| P01911 | 2WBJ | HMDB0000925 | 5.387 |
| P27986 | 1H9O | HMDB0000122 | 5.386 |
| P42858 | 3IO4 | HMDB0000925 | 5.385 |
| P05231 | 1ALU | HMDB0034146 | 5.384 |
| P08397 | 3ECR | HMDB0000925 | 5.384 |
| P02741 | 1CGP | HMDB0000122 | 5.383 |
| Q9NQC3 | 1OZN | HMDB0000925 | 5.383 |
| Q9Y617 | 3E77 | HMDB0000122 | 5.382 |
| Q15303 | 2AHX | HMDB0000123 | 5.382 |
| P15289 | 2AIJ | HMDB0000123 | 5.382 |
| P35520 | 1M54 | HMDB0000123 | 5.381 |
| Q08499 | 1MKD | HMDB0000122 | 5.38 |
| P14902 | 2D0T | HMDB0000123 | 5.38 |
| P11362 | 1CVS | HMDB0010393 | 5.379 |
| P21359 | 2D4Q | HMDB0000641 | 5.379 |
| P05067 | 2FK3 | HMDB0000122 | 5.379 |
| P16435 | 3FJO | HMDB0000122 | 5.379 |
| Q02750 | 3EQC | HMDB0000122 | 5.378 |
| P22301 | 2ILK | HMDB0000294 | 5.377 |
| P60484 | 1D5R | HMDB0000122 | 5.375 |
| P29475 | 1F20 | HMDB0000122 | 5.375 |
| P60484 | 1D5R | HMDB0010383 | 5.374 |
| P11274 | 1K1F | HMDB0000925 | 5.374 |
| Q06124 | 2SHP | HMDB0010383 | 5.374 |
| P31749 | 2UZS | HMDB0010383 | 5.374 |
| P21397 | 2Z5Y | HMDB0000122 | 5.374 |
| P42336 | 3HHM | HMDB0000122 | 5.374 |
| P00450 | 2J5W | HMDB0000925 | 5.373 |
| P11362 | 1CVS | HMDB0034146 | 5.372 |
| P10145 | 1QE6 | HMDB0000122 | 5.372 |
| P11274 | 1K1F | HMDB0034146 | 5.371 |
| P27487 | 1W1I | HMDB0000123 | 5.371 |
| Q9Y2R2 | 2P6X | HMDB0000925 | 5.371 |
| P11362 | 1CVS | HMDB0000925 | 5.37 |
| P05019 | 1H02 | HMDB0000161 | 5.37 |
| P08833 | 1ZT3 | HMDB0004610 | 5.369 |
| P15289 | 2AIJ | HMDB0000294 | 5.369 |
| Q16620 | 1HCF | HMDB0001494 | 5.368 |
| Q15303 | 2AHX | HMDB0004610 | 5.368 |
| P54098 | 1A1V | HMDB0000099 | 5.367 |
| P05019 | 1H02 | HMDB0004610 | 5.366 |
| P01911 | 2WBJ | HMDB0001494 | 5.366 |
| P08069 | 3F5P | HMDB0000122 | 5.365 |
| P02787 | 1DAN | HMDB0000122 | 5.364 |
| P01920 | 1UVQ | HMDB0000294 | 5.363 |
| P35520 | 1M54 | HMDB0000122 | 5.362 |
| Q06787 | 2QND | HMDB0000925 | 5.362 |
| Q9Y243 | 2X18 | HMDB0000122 | 5.362 |
| P06850 | 1MO1 | HMDB0000294 | 5.361 |
| P78509 | 2DDU | HMDB0000925 | 5.361 |
| P00450 | 2J5W | HMDB0004610 | 5.361 |
| Q14203 | 2HL5 | HMDB0000294 | 5.36 |
| O43612 | 1UV0 | HMDB0004610 | 5.359 |
| Q9UBK2 | 1XB7 | HMDB0000123 | 5.359 |
| P05231 | 1ALU | HMDB0001494 | 5.358 |
| P01579 | 1EKU | HMDB0004610 | 5.357 |
| Q16620 | 1HCF | HMDB0000925 | 5.357 |
| P10082 | 2ZA5 | HMDB0000122 | 5.357 |
| P26358 | 3EPZ | HMDB0034146 | 5.357 |
| P04637 | 1GZH | HMDB0000122 | 5.356 |
| P42345 | 4FAP | HMDB0001494 | 5.354 |
| P01911 | 2WBJ | HMDB0010393 | 5.352 |
| P06280 | 3HG3 | HMDB0034146 | 5.352 |
| O00555 | 3BXK | HMDB0010383 | 5.351 |
| P11362 | 1CVS | HMDB0010396 | 5.35 |
| P09619 | 1GQ5 | HMDB0010384 | 5.35 |
| O15146 | 2IEP | HMDB0000122 | 5.35 |

| | | | |
|---|---|---|---|
| P04062 | 2NT0 | HMDB0000122 | 5.35 |
| P11362 | 1CVS | HMDB0004610 | 5.349 |
| P01138 | 1SG1 | HMDB0000294 | 5.347 |
| P21964 | 3HVH | HMDB0010383 | 5.347 |
| P01579 | 1EKU | HMDB0001494 | 5.346 |
| P21333 | 2WFN | HMDB0010396 | 5.346 |
| Q06124 | 2SHP | HMDB0000641 | 5.344 |
| P05231 | 1ALU | HMDB0010396 | 5.342 |
| P54098 | 1A1V | HMDB0000122 | 5.341 |
| Q8NER1 | 2NYJ | HMDB0034146 | 5.341 |
| P08833 | 1ZT3 | HMDB0034146 | 5.339 |
| P21964 | 3HVH | HMDB0001494 | 5.339 |
| P02741 | 1CGP | HMDB0001494 | 5.338 |
| P02768 | 1E7A | HMDB0000123 | 5.338 |
| P01308 | 1EVR | HMDB0000122 | 5.338 |
| P01303 | 1R9N | HMDB0004610 | 5.337 |
| P31749 | 2UZS | HMDB0010396 | 5.336 |
| P16220 | 1DH3 | HMDB0010384 | 5.335 |
| P41159 | 3IIQ | HMDB0010383 | 5.334 |
| P41159 | 3IIQ | HMDB0000925 | 5.333 |
| P41159 | 3IIQ | HMDB0010384 | 5.332 |
| P05231 | 1ALU | HMDB0010393 | 5.33 |
| P68871 | 1BAB | HMDB0000161 | 5.328 |
| Q13255 | 1EWT | HMDB0000123 | 5.328 |
| P28329 | 2FY3 | HMDB0000122 | 5.328 |
| P16220 | 1DH3 | HMDB0010383 | 5.327 |
| P03372 | 1HCQ | HMDB0000122 | 5.327 |
| P12821 | 1UZE | HMDB0000122 | 5.326 |
| P01584 | 2NVH | HMDB0004610 | 5.326 |
| Q9Y243 | 2X18 | HMDB0001494 | 5.326 |
| P08397 | 3ECR | HMDB0001494 | 5.326 |
| P21359 | 2D4Q | HMDB0000161 | 5.324 |
| Q06124 | 2SHP | HMDB0001494 | 5.323 |
| P42345 | 4FAP | HMDB0000122 | 5.322 |
| P01911 | 2WBJ | HMDB0010396 | 5.32 |
| P60568 | 1PY2 | HMDB0000161 | 5.32 |
| P01270 | 3C4M | HMDB0000925 | 5.316 |
| P27986 | 1H9O | HMDB0001494 | 5.315 |
| P27986 | 1H9O | HMDB0034146 | 5.315 |
| P23771 | 3DFV | HMDB0000122 | 5.314 |
| P05019 | 1H02 | HMDB0000122 | 5.313 |
| P21333 | 2WFN | HMDB0010384 | 5.313 |
| P41159 | 3IIQ | HMDB0034146 | 5.313 |
| P42858 | 3IO4 | HMDB0000123 | 5.313 |
| P43354 | 1OVL | HMDB0000099 | 5.311 |
| Q13936 | 1T0J | HMDB0000294 | 5.311 |
| P31749 | 2UZS | HMDB0004610 | 5.309 |
| P49917 | 3II6 | HMDB0000122 | 5.309 |
| P21359 | 2D4Q | HMDB0000122 | 5.308 |
| Q7LG56 | 3HF1 | HMDB0004610 | 5.307 |
| P01911 | 2WBJ | HMDB0000122 | 5.306 |
| P14416 | 2HLB | HMDB0000641 | 5.305 |
| P06280 | 3HG3 | HMDB0001494 | 5.305 |
| P30793 | 1N3T | HMDB0001494 | 5.303 |
| P62834 | 3KUC | HMDB0001494 | 5.303 |
| Q7LG56 | 3HF1 | HMDB0001494 | 5.302 |
| P04049 | 3CU8 | HMDB0000099 | 5.301 |
| P01303 | 1R9N | HMDB0010383 | 5.298 |
| Q08499 | 1MKD | HMDB0000161 | 5.297 |
| Q15067 | 1W07 | HMDB0001494 | 5.297 |
| P01375 | 2AZ5 | HMDB0010384 | 5.297 |
| P39905 | 2V5E | HMDB0000122 | 5.297 |
| Q99497 | 1PDW | HMDB0000161 | 5.296 |
| Q15303 | 2AHX | HMDB0000122 | 5.292 |
| Q06787 | 2QND | HMDB0034146 | 5.292 |
| P04278 | 1LHV | HMDB0000641 | 5.291 |
| P35520 | 1M54 | HMDB0001494 | 5.29 |
| P07949 | 2IVS | HMDB0000123 | 5.29 |
| P36551 | 2AEX | HMDB0000641 | 5.289 |

| | | | |
|---|---|---|---|
| Q9Y617 | 3E77 | HMDB0000925 | 5.288 |
| P01584 | 2NVH | HMDB0001494 | 5.288 |
| P15056 | 3II5 | HMDB0001494 | 5.288 |
| P01303 | 1R9N | HMDB0010396 | 5.287 |
| P49917 | 3II6 | HMDB0034146 | 5.286 |
| P16220 | 1DH3 | HMDB0010393 | 5.285 |
| P16220 | 1DH3 | HMDB0010396 | 5.285 |
| P51659 | 1IKT | HMDB0000161 | 5.283 |
| P00450 | 2J5W | HMDB0001494 | 5.283 |
| P41159 | 3IIQ | HMDB0004610 | 5.282 |
| O43612 | 1UV0 | HMDB0000122 | 5.28 |
| P14902 | 2D0T | HMDB0000641 | 5.279 |
| Q30201 | 1DEA | HMDB0001494 | 5.278 |
| P02768 | 1E7A | HMDB0000161 | 5.275 |
| P27338 | 1OJA | HMDB0000294 | 5.275 |
| P01911 | 2WBJ | HMDB0004610 | 5.275 |
| P36551 | 2AEX | HMDB0001494 | 5.272 |
| Q9UBK2 | 1XB7 | HMDB0000122 | 5.271 |
| P21359 | 2D4Q | HMDB0001494 | 5.271 |
| P20783 | 1BND | HMDB0004610 | 5.27 |
| P23560 | 1BND | HMDB0004610 | 5.27 |
| P09619 | 1GQ5 | HMDB0000122 | 5.269 |
| P78504 | 2VJ2 | HMDB0000122 | 5.269 |
| P02741 | 1CGP | HMDB0000641 | 5.268 |
| P35520 | 1M54 | HMDB0000641 | 5.268 |
| P14902 | 2D0T | HMDB0001494 | 5.268 |
| P04049 | 3CU8 | HMDB0001494 | 5.266 |
| P60568 | 1PY2 | HMDB0001494 | 5.266 |
| P04278 | 1LHV | HMDB0001494 | 5.265 |
| P04049 | 3CU8 | HMDB0000122 | 5.265 |
| P51659 | 1IKT | HMDB0001494 | 5.264 |
| P43354 | 1OVL | HMDB0000122 | 5.263 |
| Q16620 | 1HCF | HMDB0000122 | 5.262 |
| P78509 | 2DDU | HMDB0010393 | 5.258 |
| Q7LG56 | 3HF1 | HMDB0000123 | 5.258 |
| Q03431 | 3L2J | HMDB0000641 | 5.258 |
| P55072 | 1Y8E | HMDB0000294 | 5.257 |
| P12821 | 1UZE | HMDB0001494 | 5.255 |
| P20783 | 1BND | HMDB0001494 | 5.254 |
| P23560 | 1BND | HMDB0001494 | 5.254 |
| O00555 | 3BXK | HMDB0034146 | 5.254 |
| P54098 | 1A1V | HMDB0001494 | 5.253 |
| P78504 | 2VJ2 | HMDB0000294 | 5.253 |
| P14416 | 2HLB | HMDB0001494 | 5.251 |
| P07949 | 2IVS | HMDB0000122 | 5.251 |
| Q8NER1 | 2NYJ | HMDB0001494 | 5.251 |
| P60568 | 1PY2 | HMDB0004610 | 5.251 |
| P01127 | 3MJG | HMDB0001494 | 5.251 |
| Q15465 | 3M1N | HMDB0004610 | 5.25 |
| P55072 | 1Y8E | HMDB0000925 | 5.249 |
| P27487 | 1W1I | HMDB0004610 | 5.248 |
| P01127 | 3MJG | HMDB0000122 | 5.248 |
| P05231 | 1ALU | HMDB0004610 | 5.247 |
| P04637 | 1GZH | HMDB0034146 | 5.246 |
| P04278 | 1LHV | HMDB0003681 | 5.246 |
| P41159 | 3IIQ | HMDB0001494 | 5.246 |
| P62834 | 3KUC | HMDB0000925 | 5.246 |
| P35637 | 1G5G | HMDB0004610 | 5.245 |
| P01270 | 3C4M | HMDB0000641 | 5.24 |
| P31749 | 2UZS | HMDB0034146 | 5.235 |
| Q9BYW2 | 3H6L | HMDB0001494 | 5.234 |
| P01579 | 1EKU | HMDB0034146 | 5.233 |
| P02768 | 1E7A | HMDB0000925 | 5.231 |
| P09619 | 1GQ5 | HMDB0001494 | 5.231 |
| P54098 | 1A1V | HMDB0000641 | 5.23 |
| P39905 | 2V5E | HMDB0034146 | 5.23 |
| P55072 | 1Y8E | HMDB0000122 | 5.228 |
| P28482 | 1PME | HMDB0001494 | 5.227 |
| P11274 | 1K1F | HMDB0001494 | 5.226 |

| | | | |
|---|---|---|---|
| P16220 | 1DH3 | HMDB0000161 | 5.225 |
| P01303 | 1R9N | HMDB0034146 | 5.225 |
| Q15465 | 3M1N | HMDB0001494 | 5.223 |
| Q5S007 | 3D6T | HMDB0004610 | 5.221 |
| O43612 | 1UV0 | HMDB0001494 | 5.219 |
| Q99497 | 1PDW | HMDB0001494 | 5.218 |
| P04049 | 3CU8 | HMDB0000161 | 5.217 |
| Q9UBP0 | 3B9P | HMDB0001494 | 5.216 |
| P16278 | 1BOB | HMDB0000122 | 5.215 |
| P09619 | 1GQ5 | HMDB0034146 | 5.213 |
| P10145 | 1QE6 | HMDB0034146 | 5.213 |
| P01241 | 1AXI | HMDB0001494 | 5.209 |
| P10912 | 1AXI | HMDB0001494 | 5.209 |
| P68871 | 1BAB | HMDB0000123 | 5.209 |
| P21802 | 1DJS | HMDB0000925 | 5.209 |
| P01112 | 1NVU | HMDB0001494 | 5.209 |
| P01241 | 1AXI | HMDB0004610 | 5.207 |
| P10912 | 1AXI | HMDB0004610 | 5.207 |
| Q8NFD5 | 2EH9 | HMDB0000122 | 5.206 |
| Q9BYW2 | 3H6L | HMDB0000161 | 5.204 |
| P01116 | 1N4R | HMDB0003681 | 5.203 |
| P01911 | 2WBJ | HMDB0034146 | 5.203 |
| Q9Y617 | 3E77 | HMDB0001494 | 5.201 |
| P28482 | 1PME | HMDB0000161 | 5.199 |
| P01375 | 2AZ5 | HMDB0001494 | 5.199 |
| Q9NQC3 | 1OZN | HMDB0004610 | 5.198 |
| P78509 | 2DDU | HMDB0001494 | 5.198 |
| P01375 | 2AZ5 | HMDB0000122 | 5.196 |
| Q99259 | 2OKJ | HMDB0001494 | 5.195 |
| P01116 | 1N4R | HMDB0000641 | 5.194 |
| P78509 | 2DDU | HMDB0000161 | 5.194 |
| P04637 | 1GZH | HMDB0000925 | 5.193 |
| P06850 | 1MO1 | HMDB0004610 | 5.192 |
| P10145 | 1QE6 | HMDB0000161 | 5.192 |
| Q15303 | 2AHX | HMDB0001494 | 5.192 |
| P17612 | 3MVJ | HMDB0001494 | 5.192 |
| P51608 | 3C2I | HMDB0004610 | 5.188 |
| P03372 | 1HCQ | HMDB0000925 | 5.187 |
| P05231 | 1ALU | HMDB0000122 | 5.186 |
| P78504 | 2VJ2 | HMDB0001494 | 5.186 |
| P04049 | 3CU8 | HMDB0000123 | 5.18 |
| P20783 | 1BND | HMDB0000122 | 5.179 |
| P23560 | 1BND | HMDB0000122 | 5.179 |
| P04271 | 2H61 | HMDB0001494 | 5.179 |
| P06280 | 3HG3 | HMDB0000925 | 5.177 |
| P27487 | 1W1I | HMDB0001494 | 5.176 |
| P04062 | 2NT0 | HMDB0001494 | 5.176 |
| P35637 | 1G5G | HMDB0001494 | 5.173 |
| Q99700 | 3KTR | HMDB0000123 | 5.173 |
| P10145 | 1QE6 | HMDB0000123 | 5.169 |
| P10082 | 2ZA5 | HMDB0000641 | 5.169 |
| P68871 | 1BAB | HMDB0000925 | 5.168 |
| Q9Y243 | 2X18 | HMDB0000925 | 5.168 |
| P42345 | 4FAP | HMDB0000641 | 5.168 |
| P01589 | 3IU3 | HMDB0004610 | 5.167 |
| Q08499 | 1MKD | HMDB0000641 | 5.166 |
| P55072 | 1Y8E | HMDB0001494 | 5.165 |
| P42858 | 3IO4 | HMDB0004610 | 5.165 |
| P35637 | 1G5G | HMDB0000161 | 5.164 |
| P28329 | 2FY3 | HMDB0000161 | 5.164 |
| P11362 | 1CVS | HMDB0000122 | 5.163 |
| P55072 | 1Y8E | HMDB0004610 | 5.162 |
| P12821 | 1UZE | HMDB0000641 | 5.161 |
| P05067 | 2FK3 | HMDB0000925 | 5.16 |
| P28329 | 2FY3 | HMDB0000641 | 5.16 |
| P00441 | 1HL5 | HMDB0001494 | 5.159 |
| P20783 | 1BND | HMDB0000161 | 5.157 |
| P23560 | 1BND | HMDB0000161 | 5.157 |
| P27361 | 3FXW | HMDB0000925 | 5.157 |

| | | | |
|---|---|---|---|
| P62834 | 3KUC | HMDB0000122 | 5.157 |
| Q7LG56 | 3HF1 | HMDB0000122 | 5.156 |
| Q9NQC3 | 1OZN | HMDB0001494 | 5.155 |
| P15289 | 2AIJ | HMDB0001494 | 5.155 |
| P54098 | 1A1V | HMDB0000161 | 5.154 |
| P60484 | 1D5R | HMDB0010384 | 5.154 |
| P78504 | 2VJ2 | HMDB0004610 | 5.152 |
| Q02750 | 3EQC | HMDB0000641 | 5.151 |
| P01138 | 1SG1 | HMDB0000641 | 5.15 |
| P60568 | 1PY2 | HMDB0000122 | 5.149 |
| P01579 | 1EKU | HMDB0000122 | 5.148 |
| Q14203 | 2HL5 | HMDB0000161 | 5.147 |
| Q06787 | 2QND | HMDB0000122 | 5.147 |
| P04637 | 1GZH | HMDB0001494 | 5.146 |
| P21964 | 3HVH | HMDB0000123 | 5.146 |
| Q15067 | 1W07 | HMDB0000641 | 5.144 |
| P01584 | 2NVH | HMDB0000122 | 5.144 |
| P10082 | 2ZA5 | HMDB0000099 | 5.144 |
| P08069 | 3F5P | HMDB0001494 | 5.144 |
| P00450 | 2J5W | HMDB0000161 | 5.142 |
| Q99497 | 1PDW | HMDB0000123 | 5.14 |
| P15289 | 2AIJ | HMDB0004610 | 5.139 |
| Q9BYW2 | 3H6L | HMDB0000641 | 5.139 |
| P35637 | 1G5G | HMDB0034146 | 5.138 |
| P04062 | 2NT0 | HMDB0034146 | 5.138 |
| P68871 | 1BAB | HMDB0000641 | 5.134 |
| P11274 | 1K1F | HMDB0000122 | 5.132 |
| P43354 | 1OVL | HMDB0000641 | 5.131 |
| Q5S007 | 3D6T | HMDB0001494 | 5.131 |
| P21964 | 3HVH | HMDB0000641 | 5.131 |
| P26358 | 3EPZ | HMDB0000122 | 5.129 |
| Q14203 | 2HL5 | HMDB0004610 | 5.127 |
| Q99700 | 3KTR | HMDB0000122 | 5.124 |
| Q30201 | 1DEA | HMDB0000641 | 5.123 |
| Q9NQC3 | 1OZN | HMDB0000161 | 5.123 |
| P04150 | 1P93 | HMDB0000641 | 5.122 |
| P02768 | 1E7A | HMDB0000641 | 5.12 |
| Q13255 | 1EWT | HMDB0004610 | 5.119 |
| P05067 | 2FK3 | HMDB0000099 | 5.117 |
| P08833 | 1ZT3 | HMDB0000122 | 5.116 |
| P27361 | 3FXW | HMDB0000123 | 5.116 |
| Q99497 | 1PDW | HMDB0000122 | 5.114 |
| P01584 | 2NVH | HMDB0034146 | 5.114 |
| O00555 | 3BXK | HMDB0000122 | 5.114 |
| Q7LG56 | 3HF1 | HMDB0000161 | 5.114 |
| P62834 | 3KUC | HMDB0000641 | 5.113 |
| Q9Y2R2 | 2P6X | HMDB0000122 | 5.112 |
| P31749 | 2UZS | HMDB0000122 | 5.112 |
| P05019 | 1H02 | HMDB0000123 | 5.111 |
| P00450 | 2J5W | HMDB0000122 | 5.111 |
| P01116 | 1N4R | HMDB0000123 | 5.11 |
| Q15067 | 1W07 | HMDB0000190 | 5.109 |
| Q8NFD5 | 2EH9 | HMDB0000099 | 5.108 |
| P02787 | 1DAN | HMDB0000641 | 5.107 |
| P05067 | 2FK3 | HMDB0000641 | 5.107 |
| P01584 | 2NVH | HMDB0000161 | 5.107 |
| P42336 | 3HHM | HMDB0001494 | 5.106 |
| P16220 | 1DH3 | HMDB0004610 | 5.104 |
| P27338 | 1OJA | HMDB0000641 | 5.104 |
| P00441 | 1HL5 | HMDB0000122 | 5.103 |
| Q06787 | 2QND | HMDB0000161 | 5.103 |
| P06280 | 3HG3 | HMDB0000122 | 5.103 |
| Q9Y617 | 3E77 | HMDB0000641 | 5.102 |
| Q99259 | 2OKJ | HMDB0000641 | 5.101 |
| Q9UBK2 | 1XB7 | HMDB0034146 | 5.095 |
| P01112 | 1NVU | HMDB0000122 | 5.093 |
| P08397 | 3ECR | HMDB0000641 | 5.093 |
| Q9UBP0 | 3B9P | HMDB0000122 | 5.092 |
| P29475 | 1F20 | HMDB0000641 | 5.088 |

| | | | |
|---|---|---|---|
| P04062 | 2NT0 | HMDB0000161 | 5.088 |
| P60568 | 1PY2 | HMDB0034146 | 5.087 |
| P21397 | 2Z5Y | HMDB0000641 | 5.085 |
| P41159 | 3IIQ | HMDB0000122 | 5.082 |
| P01579 | 1EKU | HMDB0000161 | 5.077 |
| P27361 | 3FXW | HMDB0000641 | 5.077 |
| Q03431 | 3L2J | HMDB0003681 | 5.077 |
| P27338 | 1OJA | HMDB0003681 | 5.076 |
| P02741 | 1CGP | HMDB0000190 | 5.072 |
| P36551 | 2AEX | HMDB0000883 | 5.072 |
| P01303 | 1R9N | HMDB0000122 | 5.071 |
| P06850 | 1MO1 | HMDB0000122 | 5.068 |
| P20783 | 1BND | HMDB0034146 | 5.067 |
| P23560 | 1BND | HMDB0034146 | 5.067 |
| P00441 | 1HL5 | HMDB0000123 | 5.067 |
| P01589 | 3IU3 | HMDB0002329 | 5.067 |
| P01589 | 3IU3 | HMDB0034146 | 5.065 |
| Q8NER1 | 2NYJ | HMDB0000122 | 5.062 |
| P16220 | 1DH3 | HMDB0000122 | 5.059 |
| Q15303 | 2AHX | HMDB0000161 | 5.059 |
| Q9Y243 | 2X18 | HMDB0000099 | 5.057 |
| P27487 | 1W1I | HMDB0000122 | 5.056 |
| O15146 | 2IEP | HMDB0034146 | 5.055 |
| Q5S007 | 3D6T | HMDB0000122 | 5.055 |
| P01589 | 3IU3 | HMDB0000122 | 5.055 |
| P16220 | 1DH3 | HMDB0034146 | 5.052 |
| P01308 | 1EVR | HMDB0000641 | 5.052 |
| Q9NQC3 | 1OZN | HMDB0000122 | 5.052 |
| P04049 | 3CU8 | HMDB0000641 | 5.051 |
| P01127 | 3MJG | HMDB0000161 | 5.051 |
| P02787 | 1DAN | HMDB0000099 | 5.05 |
| P04150 | 1P93 | HMDB0000925 | 5.05 |
| P00441 | 1HL5 | HMDB0000161 | 5.049 |
| Q06787 | 2QND | HMDB0000641 | 5.049 |
| P04150 | 1P93 | HMDB0003681 | 5.048 |
| Q9Y243 | 2X18 | HMDB0000641 | 5.048 |
| P62834 | 3KUC | HMDB0000099 | 5.048 |
| Q16620 | 1HCF | HMDB0000099 | 5.047 |
| P07949 | 2IVS | HMDB0000641 | 5.047 |
| P78504 | 2VJ2 | HMDB0000161 | 5.047 |
| Q15465 | 3M1N | HMDB0000122 | 5.047 |
| P27361 | 3FXW | HMDB0000161 | 5.046 |
| P01241 | 1AXI | HMDB0000122 | 5.044 |
| P10912 | 1AXI | HMDB0000122 | 5.044 |
| Q14203 | 2HL5 | HMDB0000122 | 5.044 |
| P42345 | 4FAP | HMDB0000161 | 5.044 |
| Q9NQC3 | 1OZN | HMDB0034146 | 5.043 |
| P30793 | 1N3T | HMDB0000641 | 5.041 |
| P55072 | 1Y8E | HMDB0000190 | 5.041 |
| P42858 | 3IO4 | HMDB0000122 | 5.041 |
| P02768 | 1E7A | HMDB0003681 | 5.04 |
| P21802 | 1DJS | HMDB0000641 | 5.039 |
| P04271 | 2H61 | HMDB0000641 | 5.039 |
| P51608 | 3C2I | HMDB0000122 | 5.039 |
| Q7LG56 | 3HF1 | HMDB0034146 | 5.037 |
| P15056 | 3II5 | HMDB0000161 | 5.035 |
| P78509 | 2DDU | HMDB0000122 | 5.034 |
| P21333 | 2WFN | HMDB0000122 | 5.034 |
| P08069 | 3F5P | HMDB0000925 | 5.034 |
| P01241 | 1AXI | HMDB0000123 | 5.033 |
| P10912 | 1AXI | HMDB0000123 | 5.033 |
| Q30201 | 1DEA | HMDB0003681 | 5.033 |
| O43612 | 1UV0 | HMDB0034146 | 5.033 |
| P28329 | 2FY3 | HMDB0000123 | 5.031 |
| P01589 | 3IU3 | HMDB0000190 | 5.03 |
| P01920 | 1UVQ | HMDB0004610 | 5.027 |
| P20783 | 1BND | HMDB0000123 | 5.026 |
| P23560 | 1BND | HMDB0000123 | 5.026 |
| P08833 | 1ZT3 | HMDB0000161 | 5.026 |

| | | | |
|---|---|---|---|
| P21397 | 2Z5Y | HMDB0001494 | 5.026 |
| P35637 | 1G5G | HMDB0000122 | 5.021 |
| P28482 | 1PME | HMDB0000123 | 5.021 |
| P16435 | 3FJO | HMDB0000099 | 5.02 |
| P22301 | 2ILK | HMDB0004610 | 5.018 |
| P08069 | 3F5P | HMDB0000099 | 5.018 |
| P01308 | 1EVR | HMDB0000161 | 5.017 |
| P05067 | 2FK3 | HMDB0000161 | 5.016 |
| Q9BYW2 | 3H6L | HMDB0000123 | 5.015 |
| P06280 | 3HG3 | HMDB0000099 | 5.015 |
| P05019 | 1H02 | HMDB0000190 | 5.013 |
| P16278 | 1BOB | HMDB0000641 | 5.012 |
| Q16620 | 1HCF | HMDB0000161 | 5.011 |
| P39905 | 2V5E | HMDB0000161 | 5.011 |
| Q9BYW2 | 3H6L | HMDB0000925 | 5.011 |
| P42336 | 3HHM | HMDB0000641 | 5.006 |
| O00555 | 3BXK | HMDB0000161 | 5.004 |
| P05019 | 1H02 | HMDB0034146 | 5.003 |
| Q99700 | 3KTR | HMDB0034146 | 5.002 |
| P09619 | 1GQ5 | HMDB0000161 | 5.001 |
| P20783 | 1BND | HMDB0000190 | 5 |
| P23560 | 1BND | HMDB0000190 | 5 |
| O43612 | 1UV0 | HMDB0000161 | 4.998 |
| Q13255 | 1EWT | HMDB0000122 | 4.994 |
| Q06124 | 2SHP | HMDB0000190 | 4.994 |
| Q16620 | 1HCF | HMDB0000641 | 4.993 |
| Q99700 | 3KTR | HMDB0002329 | 4.992 |
| P15289 | 2AIJ | HMDB0000122 | 4.989 |
| P60568 | 1PY2 | HMDB0000190 | 4.988 |
| P11362 | 1CVS | HMDB0000161 | 4.987 |
| P28482 | 1PME | HMDB0000641 | 4.987 |
| P12821 | 1UZE | HMDB0000190 | 4.987 |
| P21964 | 3HVH | HMDB0000161 | 4.986 |
| P01116 | 1N4R | HMDB0000161 | 4.984 |
| P01112 | 1NVU | HMDB0000641 | 4.984 |
| P26358 | 3EPZ | HMDB0000641 | 4.982 |
| Q99497 | 1PDW | HMDB0000099 | 4.981 |
| P02787 | 1DAN | HMDB0000161 | 4.98 |
| P22301 | 2ILK | HMDB0000122 | 4.979 |
| P27986 | 1H9O | HMDB0000925 | 4.977 |
| P43354 | 1OVL | HMDB0000161 | 4.977 |
| Q9Y243 | 2X18 | HMDB0000161 | 4.977 |
| P26358 | 3EPZ | HMDB0000161 | 4.976 |
| P04271 | 2H61 | HMDB0000161 | 4.975 |
| P15056 | 3II5 | HMDB0000123 | 4.975 |
| P68871 | 1BAB | HMDB0002329 | 4.973 |
| Q06124 | 2SHP | HMDB0002329 | 4.971 |
| Q8NFD5 | 2EH9 | HMDB0000641 | 4.968 |
| P29475 | 1F20 | HMDB0000099 | 4.967 |
| P10082 | 2ZA5 | HMDB0000161 | 4.967 |
| P11274 | 1K1F | HMDB0000161 | 4.966 |
| P01920 | 1UVQ | HMDB0000122 | 4.964 |
| P51659 | 1IKT | HMDB0000641 | 4.962 |
| Q13936 | 1T0J | HMDB0004610 | 4.962 |
| P01375 | 2AZ5 | HMDB0000161 | 4.962 |
| P42336 | 3HHM | HMDB0000161 | 4.962 |
| P31749 | 2UZS | HMDB0000161 | 4.959 |
| P01303 | 1R9N | HMDB0000161 | 4.954 |
| Q5S007 | 3D6T | HMDB0000161 | 4.953 |
| P02787 | 1DAN | HMDB0000123 | 4.951 |
| P23771 | 3DFV | HMDB0000641 | 4.951 |
| P08397 | 3ECR | HMDB0000190 | 4.951 |
| P01308 | 1EVR | HMDB0000123 | 4.949 |
| P29475 | 1F20 | HMDB0000161 | 4.949 |
| P49917 | 3II6 | HMDB0001494 | 4.948 |
| P16278 | 1BOB | HMDB0000161 | 4.945 |
| P04062 | 2NT0 | HMDB0000190 | 4.944 |
| P60484 | 1D5R | HMDB0000641 | 4.942 |
| P21397 | 2Z5Y | HMDB0000097 | 4.941 |

| | | | |
|---|---|---|---|
| P03372 | 1HCQ | HMDB0000641 | 4.939 |
| Q9UBK2 | 1XB7 | HMDB0002329 | 4.939 |
| Q99497 | 1PDW | HMDB0000641 | 4.937 |
| P42345 | 4FAP | HMDB0000925 | 4.934 |
| Q9Y617 | 3E77 | HMDB0003681 | 4.933 |
| Q13255 | 1EWT | HMDB0000190 | 4.932 |
| P42336 | 3HHM | HMDB0000123 | 4.932 |
| P14416 | 2HLB | HMDB0000190 | 4.931 |
| P36551 | 2AEX | HMDB0000097 | 4.93 |
| P05231 | 1ALU | HMDB0000161 | 4.929 |
| P21359 | 2D4Q | HMDB0000123 | 4.929 |
| P42858 | 3IO4 | HMDB0034146 | 4.929 |
| P54098 | 1A1V | HMDB0000925 | 4.928 |
| P16435 | 3FJO | HMDB0000161 | 4.926 |
| P51659 | 1IKT | HMDB0000123 | 4.925 |
| P60484 | 1D5R | HMDB0000161 | 4.924 |
| P01579 | 1EKU | HMDB0002329 | 4.924 |
| P05067 | 2FK3 | HMDB0000123 | 4.924 |
| P01241 | 1AXI | HMDB0034146 | 4.923 |
| P10912 | 1AXI | HMDB0034146 | 4.923 |
| P15289 | 2AIJ | HMDB0002329 | 4.921 |
| P01241 | 1AXI | HMDB0000190 | 4.92 |
| P10912 | 1AXI | HMDB0000190 | 4.92 |
| Q13255 | 1EWT | HMDB0002329 | 4.917 |
| P35637 | 1G5G | HMDB0000123 | 4.917 |
| P03372 | 1HCQ | HMDB0000123 | 4.917 |
| P06850 | 1MO1 | HMDB0000161 | 4.917 |
| Q02750 | 3EQC | HMDB0000161 | 4.916 |
| P51608 | 3C2I | HMDB0034146 | 4.914 |
| P01112 | 1NVU | HMDB0000161 | 4.912 |
| Q06124 | 2SHP | HMDB0000883 | 4.912 |
| P78509 | 2DDU | HMDB0000123 | 4.91 |
| P35520 | 1M54 | HMDB0002329 | 4.909 |
| Q15303 | 2AHX | HMDB0034146 | 4.909 |
| P10082 | 2ZA5 | HMDB0001494 | 4.908 |
| P27338 | 1OJA | HMDB0000097 | 4.907 |
| P12821 | 1UZE | HMDB0002329 | 4.906 |
| P04049 | 3CU8 | HMDB0000925 | 4.906 |
| P11362 | 1CVS | HMDB0002329 | 4.905 |
| P06280 | 3HG3 | HMDB0000161 | 4.905 |
| P11362 | 1CVS | HMDB0000641 | 4.904 |
| Q14203 | 2HL5 | HMDB0000123 | 4.902 |
| P10082 | 2ZA5 | HMDB0000925 | 4.9 |
| P15056 | 3II5 | HMDB0002329 | 4.899 |
| P04637 | 1GZH | HMDB0000161 | 4.896 |
| P54098 | 1A1V | HMDB0000123 | 4.895 |
| P05231 | 1ALU | HMDB0002329 | 4.894 |
| P01375 | 2AZ5 | HMDB0000641 | 4.892 |
| P27487 | 1W1I | HMDB0034146 | 4.891 |
| P08833 | 1ZT3 | HMDB0000099 | 4.891 |
| P60568 | 1PY2 | HMDB0002329 | 4.89 |
| P29475 | 1F20 | HMDB0002329 | 4.887 |
| P01241 | 1AXI | HMDB0000161 | 4.886 |
| P10912 | 1AXI | HMDB0000161 | 4.886 |
| P00441 | 1HL5 | HMDB0000641 | 4.886 |
| P03372 | 1HCQ | HMDB0000161 | 4.885 |
| Q06787 | 2QND | HMDB0002329 | 4.885 |
| P02787 | 1DAN | HMDB0001494 | 4.883 |
| Q16620 | 1HCF | HMDB0002329 | 4.882 |
| P01270 | 3C4M | HMDB0000190 | 4.882 |
| P54098 | 1A1V | HMDB0002329 | 4.881 |
| P31749 | 2UZS | HMDB0002329 | 4.878 |
| P01270 | 3C4M | HMDB0002329 | 4.878 |
| P01584 | 2NVH | HMDB0000190 | 4.876 |
| Q9BYW2 | 3H6L | HMDB0002329 | 4.873 |
| P04637 | 1GZH | HMDB0000641 | 4.872 |
| Q8NFD5 | 2EH9 | HMDB0000161 | 4.87 |
| Q14203 | 2HL5 | HMDB0034146 | 4.866 |
| Q15465 | 3M1N | HMDB0000161 | 4.866 |

| | | | |
|---|---|---|---|
| P39905 | 2V5E | HMDB0002329 | 4.865 |
| P62834 | 3KUC | HMDB0000161 | 4.863 |
| P42345 | 4FAP | HMDB0002329 | 4.862 |
| P16435 | 3FJO | HMDB0002329 | 4.861 |
| P42336 | 3HHM | HMDB0002329 | 4.861 |
| Q9UBK2 | 1XB7 | HMDB0000190 | 4.86 |
| P04049 | 3CU8 | HMDB0002329 | 4.86 |
| P01584 | 2NVH | HMDB0002329 | 4.859 |
| Q8NER1 | 2NYJ | HMDB0000161 | 4.856 |
| P16278 | 1BOB | HMDB0000123 | 4.855 |
| Q99259 | 2OKJ | HMDB0000883 | 4.855 |
| Q06787 | 2QND | HMDB0000123 | 4.854 |
| Q30201 | 1DEA | HMDB0002329 | 4.853 |
| P08069 | 3F5P | HMDB0000161 | 4.853 |
| Q7LG56 | 3HF1 | HMDB0002329 | 4.853 |
| P01127 | 3MJG | HMDB0002329 | 4.853 |
| P04049 | 3CU8 | HMDB0000190 | 4.85 |
| P62834 | 3KUC | HMDB0002329 | 4.849 |
| P60568 | 1PY2 | HMDB0000123 | 4.845 |
| P01584 | 2NVH | HMDB0000123 | 4.844 |
| P11274 | 1K1F | HMDB0000099 | 4.842 |
| P27338 | 1OJA | HMDB0000883 | 4.842 |
| P27338 | 1OJA | HMDB0000190 | 4.841 |
| P43354 | 1OVL | HMDB0000925 | 4.841 |
| Q14203 | 2HL5 | HMDB0000190 | 4.841 |
| P27986 | 1H9O | HMDB0000161 | 4.84 |
| P06850 | 1MO1 | HMDB0034146 | 4.84 |
| O43612 | 1UV0 | HMDB0002329 | 4.84 |
| P23771 | 3DFV | HMDB0000161 | 4.839 |
| P41159 | 3IIQ | HMDB0000161 | 4.838 |
| P04637 | 1GZH | HMDB0000099 | 4.837 |
| P01116 | 1N4R | HMDB0000097 | 4.837 |
| P00450 | 2J5W | HMDB0002329 | 4.836 |
| Q9UBP0 | 3B9P | HMDB0000641 | 4.836 |
| O00555 | 3BXK | HMDB0002329 | 4.835 |
| Q9Y617 | 3E77 | HMDB0000190 | 4.834 |
| Q30201 | 1DEA | HMDB0000190 | 4.834 |
| P49917 | 3II6 | HMDB0000641 | 4.834 |
| P04271 | 2H61 | HMDB0002329 | 4.833 |
| P31749 | 2UZS | HMDB0000190 | 4.83 |
| P01579 | 1EKU | HMDB0000190 | 4.828 |
| P27986 | 1H9O | HMDB0000099 | 4.824 |
| P04150 | 1P93 | HMDB0000161 | 4.824 |
| P04062 | 2NT0 | HMDB0000123 | 4.824 |
| Q06787 | 2QND | HMDB0000190 | 4.823 |
| Q9Y243 | 2X18 | HMDB0000190 | 4.823 |
| P26358 | 3EPZ | HMDB0000190 | 4.822 |
| Q08499 | 1MKD | HMDB0001494 | 4.821 |
| P05231 | 1ALU | HMDB0000190 | 4.819 |
| Q13936 | 1T0J | HMDB0000122 | 4.819 |
| P17612 | 3MVJ | HMDB0000883 | 4.819 |
| P01241 | 1AXI | HMDB0002329 | 4.818 |
| P10912 | 1AXI | HMDB0002329 | 4.818 |
| Q30201 | 1DEA | HMDB0000883 | 4.818 |
| P01112 | 1NVU | HMDB0000123 | 4.818 |
| O15146 | 2IEP | HMDB0000161 | 4.817 |
| P05067 | 2FK3 | HMDB0001494 | 4.816 |
| P11362 | 1CVS | HMDB0000190 | 4.815 |
| O43612 | 1UV0 | HMDB0000123 | 4.815 |
| O00555 | 3BXK | HMDB0000190 | 4.815 |
| P07949 | 2IVS | HMDB0002329 | 4.814 |
| Q9NQC3 | 1OZN | HMDB0000123 | 4.813 |
| Q9Y2R2 | 2P6X | HMDB0000641 | 4.813 |
| P01579 | 1EKU | HMDB0000641 | 4.812 |
| P04278 | 1LHV | HMDB0002329 | 4.811 |
| P14902 | 2D0T | HMDB0000190 | 4.811 |
| P09619 | 1GQ5 | HMDB0000641 | 4.81 |
| P60484 | 1D5R | HMDB0000925 | 4.808 |
| Q16620 | 1HCF | HMDB0000190 | 4.806 |

| | | | |
|---|---|---|---|
| P27361 | 3FXW | HMDB0002329 | 4.805 |
| P55072 | 1Y8E | HMDB0002329 | 4.803 |
| P04637 | 1GZH | HMDB0000123 | 4.802 |
| P11274 | 1K1F | HMDB0000641 | 4.799 |
| P01138 | 1SG1 | HMDB0000925 | 4.798 |
| P22301 | 2ILK | HMDB0034146 | 4.798 |
| P27986 | 1H9O | HMDB0002329 | 4.797 |
| P04637 | 1GZH | HMDB0002329 | 4.796 |
| Q15067 | 1W07 | HMDB0000883 | 4.795 |
| P21359 | 2D4Q | HMDB0003681 | 4.794 |
| P35637 | 1G5G | HMDB0002329 | 4.793 |
| Q99259 | 2OKJ | HMDB0000097 | 4.793 |
| P04278 | 1LHV | HMDB0000883 | 4.792 |
| Q9Y2R2 | 2P6X | HMDB0000099 | 4.79 |
| P39905 | 2V5E | HMDB0000123 | 4.789 |
| P09619 | 1GQ5 | HMDB0000099 | 4.788 |
| P02741 | 1CGP | HMDB0000883 | 4.786 |
| P11362 | 1CVS | HMDB0000123 | 4.785 |
| P08833 | 1ZT3 | HMDB0000190 | 4.785 |
| P04150 | 1P93 | HMDB0000123 | 4.783 |
| P05231 | 1ALU | HMDB0000641 | 4.782 |
| Q99497 | 1PDW | HMDB0002329 | 4.782 |
| Q13936 | 1T0J | HMDB0034146 | 4.782 |
| P08833 | 1ZT3 | HMDB0000123 | 4.782 |
| P06280 | 3HG3 | HMDB0000190 | 4.781 |
| P60484 | 1D5R | HMDB0000190 | 4.78 |
| Q8NER1 | 2NYJ | HMDB0000641 | 4.78 |
| P68871 | 1BAB | HMDB0000190 | 4.779 |
| P01112 | 1NVU | HMDB0000925 | 4.774 |
| O00555 | 3BXK | HMDB0000123 | 4.774 |
| P01920 | 1UVQ | HMDB0034146 | 4.773 |
| Q02750 | 3EQC | HMDB0001494 | 4.771 |
| P21333 | 2WFN | HMDB0000641 | 4.77 |
| P54098 | 1A1V | HMDB0000190 | 4.769 |
| P27986 | 1H9O | HMDB0000641 | 4.769 |
| P21802 | 1DJS | HMDB0000190 | 4.766 |
| Q13255 | 1EWT | HMDB0034146 | 4.766 |
| Q5S007 | 3D6T | HMDB0002329 | 4.766 |
| P08397 | 3ECR | HMDB0003681 | 4.766 |
| P10082 | 2ZA5 | HMDB0002329 | 4.765 |
| P29475 | 1F20 | HMDB0000190 | 4.763 |
| P35637 | 1G5G | HMDB0000190 | 4.761 |
| P01127 | 3MJG | HMDB0000123 | 4.761 |
| P16278 | 1BOB | HMDB0000190 | 4.759 |
| Q9UBK2 | 1XB7 | HMDB0000641 | 4.753 |
| P15289 | 2AIJ | HMDB0034146 | 4.753 |
| Q8NER1 | 2NYJ | HMDB0000190 | 4.753 |
| P02741 | 1CGP | HMDB0002329 | 4.752 |
| P01911 | 2WBJ | HMDB0000161 | 4.751 |
| P06850 | 1MO1 | HMDB0000123 | 4.75 |
| Q9Y2R2 | 2P6X | HMDB0000161 | 4.75 |
| Q9Y617 | 3E77 | HMDB0002329 | 4.749 |
| P51659 | 1IKT | HMDB0000190 | 4.749 |
| P01579 | 1EKU | HMDB0000123 | 4.748 |
| P08069 | 3F5P | HMDB0000123 | 4.748 |
| P28329 | 2FY3 | HMDB0000097 | 4.746 |
| P09619 | 1GQ5 | HMDB0000123 | 4.744 |
| P21333 | 2WFN | HMDB0000161 | 4.744 |
| P43354 | 1OVL | HMDB0000123 | 4.742 |
| P42336 | 3HHM | HMDB0000925 | 4.741 |
| Q8NFD5 | 2EH9 | HMDB0000190 | 4.74 |
| Q14203 | 2HL5 | HMDB0002329 | 4.739 |
| Q9Y617 | 3E77 | HMDB0000097 | 4.738 |
| P01112 | 1NVU | HMDB0000190 | 4.732 |
| Q9UBP0 | 3B9P | HMDB0000161 | 4.732 |
| P14902 | 2D0T | HMDB0003681 | 4.73 |
| Q02750 | 3EQC | HMDB0003681 | 4.73 |
| P16435 | 3FJO | HMDB0000925 | 4.729 |
| P02741 | 1CGP | HMDB0000097 | 4.726 |

| | | | |
|---|---|---|---|
| P27487 | 1W1I | HMDB0000190 | 4.726 |
| P10082 | 2ZA5 | HMDB0000190 | 4.725 |
| P51608 | 3C2I | HMDB0002329 | 4.725 |
| P42858 | 3IO4 | HMDB0002329 | 4.725 |
| P06850 | 1MO1 | HMDB0002329 | 4.723 |
| P36551 | 2AEX | HMDB0002329 | 4.723 |
| P51659 | 1IKT | HMDB0003681 | 4.722 |
| P41159 | 3IIQ | HMDB0002329 | 4.722 |
| P78509 | 2DDU | HMDB0002329 | 4.721 |
| P00441 | 1HL5 | HMDB0002329 | 4.719 |
| P27986 | 1H9O | HMDB0000123 | 4.715 |
| P06850 | 1MO1 | HMDB0000190 | 4.713 |
| O15146 | 2IEP | HMDB0000123 | 4.712 |
| P04271 | 2H61 | HMDB0000123 | 4.709 |
| P08069 | 3F5P | HMDB0002329 | 4.704 |
| P01303 | 1R9N | HMDB0000123 | 4.703 |
| P16435 | 3FJO | HMDB0000123 | 4.703 |
| P42345 | 4FAP | HMDB0000123 | 4.698 |
| P00450 | 2J5W | HMDB0000099 | 4.697 |
| P00450 | 2J5W | HMDB0000641 | 4.696 |
| P01375 | 2AZ5 | HMDB0000123 | 4.692 |
| P01138 | 1SG1 | HMDB0000161 | 4.685 |
| P68871 | 1BAB | HMDB0003681 | 4.683 |
| P01116 | 1N4R | HMDB0000883 | 4.683 |
| P55072 | 1Y8E | HMDB0034146 | 4.682 |
| P08833 | 1ZT3 | HMDB0000641 | 4.682 |
| P28482 | 1PME | HMDB0003681 | 4.679 |
| P42858 | 3IO4 | HMDB0000190 | 4.678 |
| Q15465 | 3M1N | HMDB0000123 | 4.675 |
| Q16620 | 1HCF | HMDB0000123 | 4.674 |
| P78509 | 2DDU | HMDB0000190 | 4.672 |
| Q9NQC3 | 1OZN | HMDB0000190 | 4.671 |
| P04271 | 2H61 | HMDB0003681 | 4.67 |
| P21333 | 2WFN | HMDB0000190 | 4.67 |
| Q9Y243 | 2X18 | HMDB0000123 | 4.668 |
| Q08499 | 1MKD | HMDB0000097 | 4.667 |
| P04278 | 1LHV | HMDB0000097 | 4.665 |
| P49917 | 3II6 | HMDB0000161 | 4.664 |
| P05231 | 1ALU | HMDB0000099 | 4.661 |
| P02768 | 1E7A | HMDB0000097 | 4.661 |
| P04278 | 1LHV | HMDB0000190 | 4.66 |
| P01138 | 1SG1 | HMDB0000097 | 4.659 |
| Q08499 | 1MKD | HMDB0000883 | 4.655 |
| P62834 | 3KUC | HMDB0000123 | 4.655 |
| P00441 | 1HL5 | HMDB0000925 | 4.65 |
| P01911 | 2WBJ | HMDB0000123 | 4.65 |
| P28329 | 2FY3 | HMDB0000883 | 4.648 |
| Q5S007 | 3D6T | HMDB0000123 | 4.645 |
| P31749 | 2UZS | HMDB0000641 | 4.64 |
| P04278 | 1LHV | HMDB0000094 | 4.636 |
| P21359 | 2D4Q | HMDB0000094 | 4.636 |
| Q9Y2R2 | 2P6X | HMDB0000190 | 4.636 |
| Q9Y2R2 | 2P6X | HMDB0002329 | 4.634 |
| P16220 | 1DH3 | HMDB0000123 | 4.633 |
| P39905 | 2V5E | HMDB0000099 | 4.633 |
| P60484 | 1D5R | HMDB0000123 | 4.63 |
| P01116 | 1N4R | HMDB0000094 | 4.629 |
| P04150 | 1P93 | HMDB0000094 | 4.627 |
| P36551 | 2AEX | HMDB0060348 | 4.625 |
| P05231 | 1ALU | HMDB0000123 | 4.623 |
| P39905 | 2V5E | HMDB0000641 | 4.62 |
| P41159 | 3IIQ | HMDB0000123 | 4.618 |
| P26358 | 3EPZ | HMDB0000123 | 4.615 |
| P29475 | 1F20 | HMDB0000123 | 4.611 |
| Q9UBP0 | 3B9P | HMDB0000190 | 4.61 |
| P21397 | 2Z5Y | HMDB0002329 | 4.608 |
| P01584 | 2NVH | HMDB0000099 | 4.605 |
| P02768 | 1E7A | HMDB0000094 | 4.604 |
| Q8NER1 | 2NYJ | HMDB0000123 | 4.602 |

| | | | |
|---|---|---|---|
| P04150 | 1P93 | HMDB0060348 | 4.599 |
| P35520 | 1M54 | HMDB0003681 | 4.598 |
| P04278 | 1LHV | HMDB0060348 | 4.595 |
| P41159 | 3IIQ | HMDB0000641 | 4.595 |
| Q06124 | 2SHP | HMDB0000097 | 4.594 |
| Q03431 | 3L2J | HMDB0060348 | 4.593 |
| Q15067 | 1W07 | HMDB0000097 | 4.592 |
| P27986 | 1H9O | HMDB0000190 | 4.585 |
| P30793 | 1N3T | HMDB0000097 | 4.585 |
| P01138 | 1SG1 | HMDB0000883 | 4.582 |
| Q9BYW2 | 3H6L | HMDB0000094 | 4.578 |
| P00441 | 1HL5 | HMDB0000190 | 4.577 |
| Q9Y617 | 3E77 | HMDB0000883 | 4.573 |
| P06280 | 3HG3 | HMDB0000123 | 4.571 |
| P02768 | 1E7A | HMDB0000883 | 4.57 |
| P27338 | 1OJA | HMDB0000161 | 4.57 |
| Q15465 | 3M1N | HMDB0000641 | 4.57 |
| P11274 | 1K1F | HMDB0000123 | 4.568 |
| P10145 | 1QE6 | HMDB0000641 | 4.565 |
| P27361 | 3FXW | HMDB0000883 | 4.565 |
| P21802 | 1DJS | HMDB0000883 | 4.564 |
| P21964 | 3HVH | HMDB0000883 | 4.559 |
| P01270 | 3C4M | HMDB0060348 | 4.554 |
| Q9BYW2 | 3H6L | HMDB0000190 | 4.55 |
| P01911 | 2WBJ | HMDB0000641 | 4.546 |
| Q30201 | 1DEA | HMDB0060348 | 4.545 |
| P14416 | 2HLB | HMDB0000097 | 4.542 |
| Q30201 | 1DEA | HMDB0000097 | 4.535 |
| P28329 | 2FY3 | HMDB0060348 | 4.535 |
| Q08499 | 1MKD | HMDB0060348 | 4.534 |
| P21359 | 2D4Q | HMDB0060348 | 4.531 |
| P23771 | 3DFV | HMDB0000123 | 4.53 |
| P42336 | 3HHM | HMDB0003681 | 4.527 |
| Q8NFD5 | 2EH9 | HMDB0000123 | 4.525 |
| P21397 | 2Z5Y | HMDB0060348 | 4.524 |
| Q30201 | 1DEA | HMDB0000094 | 4.523 |
| P42336 | 3HHM | HMDB0000094 | 4.518 |
| P08397 | 3ECR | HMDB0000097 | 4.516 |
| P04062 | 2NT0 | HMDB0000099 | 4.512 |
| P01303 | 1R9N | HMDB0000641 | 4.511 |
| Q99259 | 2OKJ | HMDB0060348 | 4.508 |
| P01308 | 1EVR | HMDB0000094 | 4.507 |
| O15146 | 2IEP | HMDB0000641 | 4.506 |
| P21802 | 1DJS | HMDB0060348 | 4.5 |
| Q03431 | 3L2J | HMDB0000883 | 4.499 |
| P02768 | 1E7A | HMDB0060348 | 4.491 |
| P08397 | 3ECR | HMDB0000883 | 4.491 |
| P14416 | 2HLB | HMDB0000883 | 4.489 |
| Q06124 | 2SHP | HMDB0060348 | 4.489 |
| P27361 | 3FXW | HMDB0000094 | 4.488 |
| P27338 | 1OJA | HMDB0060348 | 4.487 |
| Q15067 | 1W07 | HMDB0060348 | 4.481 |
| P68871 | 1BAB | HMDB0060348 | 4.48 |
| P30793 | 1N3T | HMDB0000883 | 4.479 |
| P49917 | 3II6 | HMDB0000190 | 4.479 |
| P02787 | 1DAN | HMDB0003681 | 4.478 |
| P01308 | 1EVR | HMDB0060348 | 4.477 |
| P28482 | 1PME | HMDB0060348 | 4.477 |
| O43612 | 1UV0 | HMDB0000099 | 4.477 |
| P15056 | 3II5 | HMDB0003681 | 4.474 |
| P21333 | 2WFN | HMDB0000123 | 4.473 |
| P01308 | 1EVR | HMDB0001494 | 4.467 |
| P01308 | 1EVR | HMDB0000883 | 4.462 |
| P10145 | 1QE6 | HMDB0000099 | 4.462 |
| P28482 | 1PME | HMDB0000094 | 4.457 |
| Q9Y617 | 3E77 | HMDB0060348 | 4.455 |
| P36551 | 2AEX | HMDB0000094 | 4.454 |
| Q08499 | 1MKD | HMDB0000094 | 4.453 |
| P04150 | 1P93 | HMDB0000883 | 4.452 |

| | | | |
|---|---|---|---|
| P10082 | 2ZA5 | HMDB0003681 | 4.451 |
| P01270 | 3C4M | HMDB0000097 | 4.451 |
| P21964 | 3HVH | HMDB0000094 | 4.447 |
| P04637 | 1GZH | HMDB0000190 | 4.446 |
| P27361 | 3FXW | HMDB0060348 | 4.446 |
| P27361 | 3FXW | HMDB0000097 | 4.445 |
| P42336 | 3HHM | HMDB0000190 | 4.444 |
| P01138 | 1SG1 | HMDB0000123 | 4.442 |
| Q08499 | 1MKD | HMDB0000190 | 4.439 |
| P01270 | 3C4M | HMDB0000094 | 4.433 |
| Q15465 | 3M1N | HMDB0000099 | 4.431 |
| Q5S007 | 3D6T | HMDB0000099 | 4.43 |
| P21802 | 1DJS | HMDB0000094 | 4.428 |
| P30793 | 1N3T | HMDB0060348 | 4.426 |
| P21964 | 3HVH | HMDB0060348 | 4.425 |
| P51659 | 1IKT | HMDB0000094 | 4.421 |
| O43612 | 1UV0 | HMDB0000641 | 4.421 |
| Q15303 | 2AHX | HMDB0000641 | 4.421 |
| P14902 | 2D0T | HMDB0000094 | 4.421 |
| Q7LG56 | 3HF1 | HMDB0000641 | 4.419 |
| P15056 | 3II5 | HMDB0000094 | 4.415 |
| P01138 | 1SG1 | HMDB0060348 | 4.411 |
| P12821 | 1UZE | HMDB0000097 | 4.409 |
| P04271 | 2H61 | HMDB0000094 | 4.409 |
| P01589 | 3IU3 | HMDB0000099 | 4.407 |
| P05067 | 2FK3 | HMDB0000094 | 4.405 |
| Q02750 | 3EQC | HMDB0000094 | 4.4 |
| Q03431 | 3L2J | HMDB0000097 | 4.395 |
| P68871 | 1BAB | HMDB0000094 | 4.386 |
| Q06124 | 2SHP | HMDB0000094 | 4.384 |
| P07949 | 2IVS | HMDB0060348 | 4.383 |
| Q03431 | 3L2J | HMDB0000094 | 4.382 |
| P28329 | 2FY3 | HMDB0001494 | 4.38 |
| P17612 | 3MVJ | HMDB0000094 | 4.377 |
| P01308 | 1EVR | HMDB0000097 | 4.371 |
| Q9Y2R2 | 2P6X | HMDB0000123 | 4.364 |
| P08069 | 3F5P | HMDB0000094 | 4.362 |
| Q9UBP0 | 3B9P | HMDB0000123 | 4.357 |
| P01270 | 3C4M | HMDB0000883 | 4.357 |
| P04150 | 1P93 | HMDB0000097 | 4.356 |
| P01112 | 1NVU | HMDB0003681 | 4.355 |
| P35637 | 1G5G | HMDB0000099 | 4.354 |
| P01138 | 1SG1 | HMDB0000094 | 4.35 |
| P68871 | 1BAB | HMDB0000883 | 4.345 |
| P04150 | 1P93 | HMDB0001494 | 4.344 |
| P08397 | 3ECR | HMDB0060348 | 4.344 |
| P08069 | 3F5P | HMDB0000190 | 4.342 |
| P60568 | 1PY2 | HMDB0000099 | 4.336 |
| P28329 | 2FY3 | HMDB0000094 | 4.332 |
| P03372 | 1HCQ | HMDB0003681 | 4.331 |
| P03372 | 1HCQ | HMDB0000190 | 4.327 |
| P05067 | 2FK3 | HMDB0000883 | 4.325 |
| P03372 | 1HCQ | HMDB0000094 | 4.32 |
| P05019 | 1H02 | HMDB0000641 | 4.317 |
| Q99700 | 3KTR | HMDB0000641 | 4.317 |
| P12821 | 1UZE | HMDB0000883 | 4.314 |
| P54098 | 1A1V | HMDB0003681 | 4.31 |
| P23771 | 3DFV | HMDB0000094 | 4.308 |
| P02787 | 1DAN | HMDB0000883 | 4.303 |
| P42345 | 4FAP | HMDB0060348 | 4.302 |
| P27487 | 1W1I | HMDB0000099 | 4.296 |
| Q8NFD5 | 2EH9 | HMDB0003681 | 4.295 |
| Q9NQC3 | 1OZN | HMDB0000099 | 4.292 |
| P08397 | 3ECR | HMDB0000094 | 4.292 |
| Q02750 | 3EQC | HMDB0060348 | 4.292 |
| P07949 | 2IVS | HMDB0000094 | 4.287 |
| O00555 | 3BXK | HMDB0000641 | 4.282 |
| P14902 | 2D0T | HMDB0000883 | 4.281 |
| P23771 | 3DFV | HMDB0003681 | 4.28 |

| | | | |
|---|---|---|---|
| P16220 | 1DH3 | HMDB0000641 | 4.278 |
| P06850 | 1MO1 | HMDB0000099 | 4.274 |
| P04049 | 3CU8 | HMDB0000094 | 4.273 |
| P54098 | 1A1V | HMDB0000094 | 4.271 |
| P02787 | 1DAN | HMDB0000094 | 4.271 |
| P04150 | 1P93 | HMDB0002329 | 4.27 |
| P04049 | 3CU8 | HMDB0003681 | 4.268 |
| P14416 | 2HLB | HMDB0000094 | 4.262 |
| Q15067 | 1W07 | HMDB0000094 | 4.261 |
| P01241 | 1AXI | HMDB0000099 | 4.26 |
| P10912 | 1AXI | HMDB0000099 | 4.26 |
| P01308 | 1EVR | HMDB0000190 | 4.26 |
| P07949 | 2IVS | HMDB0000883 | 4.258 |
| P08069 | 3F5P | HMDB0060348 | 4.258 |
| P51608 | 3C2I | HMDB0000099 | 4.254 |
| P30793 | 1N3T | HMDB0000094 | 4.253 |
| P10082 | 2ZA5 | HMDB0000094 | 4.253 |
| P29475 | 1F20 | HMDB0000094 | 4.252 |
| P03372 | 1HCQ | HMDB0002329 | 4.251 |
| Q14203 | 2HL5 | HMDB0000099 | 4.249 |
| P43354 | 1OVL | HMDB0000094 | 4.248 |
| P35637 | 1G5G | HMDB0000641 | 4.247 |
| P42345 | 4FAP | HMDB0000094 | 4.247 |
| P10145 | 1QE6 | HMDB0000094 | 4.246 |
| P03372 | 1HCQ | HMDB0060348 | 4.245 |
| P78504 | 2VJ2 | HMDB0000099 | 4.24 |
| P16278 | 1BOB | HMDB0000094 | 4.237 |
| P01579 | 1EKU | HMDB0000094 | 4.237 |
| P05067 | 2FK3 | HMDB0060348 | 4.234 |
| P05019 | 1H02 | HMDB0000094 | 4.228 |
| P10082 | 2ZA5 | HMDB0060348 | 4.225 |
| P16435 | 3FJO | HMDB0000094 | 4.222 |
| P39905 | 2V5E | HMDB0000094 | 4.219 |
| P60568 | 1PY2 | HMDB0000094 | 4.218 |
| Q13255 | 1EWT | HMDB0000099 | 4.217 |
| P16278 | 1BOB | HMDB0003681 | 4.216 |
| P60484 | 1D5R | HMDB0000094 | 4.214 |
| P02787 | 1DAN | HMDB0060348 | 4.213 |
| Q9BYW2 | 3H6L | HMDB0000883 | 4.213 |
| P27986 | 1H9O | HMDB0000094 | 4.211 |
| P35520 | 1M54 | HMDB0060348 | 4.21 |
| Q99497 | 1PDW | HMDB0000094 | 4.209 |
| P21802 | 1DJS | HMDB0000097 | 4.206 |
| P12821 | 1UZE | HMDB0000094 | 4.206 |
| O15146 | 2IEP | HMDB0000094 | 4.205 |
| P28482 | 1PME | HMDB0000883 | 4.204 |
| P01375 | 2AZ5 | HMDB0000094 | 4.204 |
| Q02750 | 3EQC | HMDB0000883 | 4.2 |
| Q9NQC3 | 1OZN | HMDB0000641 | 4.197 |
| P16220 | 1DH3 | HMDB0000094 | 4.196 |
| P21359 | 2D4Q | HMDB0000883 | 4.196 |
| Q99700 | 3KTR | HMDB0000094 | 4.195 |
| P27487 | 1W1I | HMDB0000641 | 4.194 |
| Q9Y617 | 3E77 | HMDB0000094 | 4.19 |
| P35520 | 1M54 | HMDB0000094 | 4.189 |
| P14902 | 2D0T | HMDB0000097 | 4.188 |
| P11274 | 1K1F | HMDB0003681 | 4.187 |
| P35520 | 1M54 | HMDB0000883 | 4.187 |
| O43612 | 1UV0 | HMDB0000094 | 4.187 |
| P20783 | 1BND | HMDB0000641 | 4.186 |
| P23560 | 1BND | HMDB0000641 | 4.186 |
| P00441 | 1HL5 | HMDB0003681 | 4.186 |
| P01112 | 1NVU | HMDB0000094 | 4.186 |
| P43354 | 1OVL | HMDB0060348 | 4.181 |
| P04062 | 2NT0 | HMDB0000094 | 4.181 |
| P60568 | 1PY2 | HMDB0000641 | 4.18 |
| P01127 | 3MJG | HMDB0000094 | 4.18 |
| Q13255 | 1EWT | HMDB0000094 | 4.179 |
| P01584 | 2NVH | HMDB0000094 | 4.179 |

| | | | |
|---|---|---|---|
| P20783 | 1BND | HMDB0000094 | 4.178 |
| P23560 | 1BND | HMDB0000094 | 4.178 |
| O00555 | 3BXK | HMDB0000094 | 4.178 |
| P01589 | 3IU3 | HMDB0000094 | 4.176 |
| Q9UBK2 | 1XB7 | HMDB0000094 | 4.175 |
| P01589 | 3IU3 | HMDB0000641 | 4.175 |
| P78504 | 2VJ2 | HMDB0000094 | 4.174 |
| P51659 | 1IKT | HMDB0000883 | 4.173 |
| Q14203 | 2HL5 | HMDB0000094 | 4.171 |
| Q9Y243 | 2X18 | HMDB0000094 | 4.171 |
| P15289 | 2AIJ | HMDB0000094 | 4.17 |
| P01911 | 2WBJ | HMDB0000094 | 4.17 |
| P62834 | 3KUC | HMDB0000094 | 4.169 |
| Q15303 | 2AHX | HMDB0000094 | 4.167 |
| P51608 | 3C2I | HMDB0000094 | 4.167 |
| P04049 | 3CU8 | HMDB0060348 | 4.166 |
| P08069 | 3F5P | HMDB0000883 | 4.166 |
| P27487 | 1W1I | HMDB0000094 | 4.165 |
| P01920 | 1UVQ | HMDB0000099 | 4.164 |
| P04637 | 1GZH | HMDB0000094 | 4.162 |
| P54098 | 1A1V | HMDB0060348 | 4.161 |
| P01138 | 1SG1 | HMDB0000190 | 4.161 |
| P05231 | 1ALU | HMDB0000094 | 4.158 |
| P42858 | 3IO4 | HMDB0000094 | 4.158 |
| P01241 | 1AXI | HMDB0000094 | 4.157 |
| P10912 | 1AXI | HMDB0000094 | 4.157 |
| Q5S007 | 3D6T | HMDB0000641 | 4.156 |
| P09619 | 1GQ5 | HMDB0000094 | 4.155 |
| Q8NFD5 | 2EH9 | HMDB0000094 | 4.155 |
| P27986 | 1H9O | HMDB0003681 | 4.154 |
| P55072 | 1Y8E | HMDB0000094 | 4.151 |
| Q9NQC3 | 1OZN | HMDB0000094 | 4.15 |
| Q15465 | 3M1N | HMDB0000094 | 4.15 |
| P04271 | 2H61 | HMDB0000883 | 4.147 |
| P23771 | 3DFV | HMDB0060348 | 4.147 |
| P49917 | 3II6 | HMDB0000094 | 4.147 |
| Q9NQC3 | 1OZN | HMDB0003681 | 4.146 |
| P60568 | 1PY2 | HMDB0060348 | 4.145 |
| P02741 | 1CGP | HMDB0000094 | 4.144 |
| P00441 | 1HL5 | HMDB0000094 | 4.142 |
| P06850 | 1MO1 | HMDB0000094 | 4.142 |
| Q7LG56 | 3HF1 | HMDB0000094 | 4.141 |
| P11362 | 1CVS | HMDB0060348 | 4.14 |
| Q5S007 | 3D6T | HMDB0003681 | 4.14 |
| P16278 | 1BOB | HMDB0060348 | 4.138 |
| P04062 | 2NT0 | HMDB0060348 | 4.137 |
| P11362 | 1CVS | HMDB0000094 | 4.136 |
| P01920 | 1UVQ | HMDB0000094 | 4.135 |
| P29475 | 1F20 | HMDB0060348 | 4.134 |
| Q99700 | 3KTR | HMDB0060348 | 4.134 |
| Q9BYW2 | 3H6L | HMDB0000097 | 4.133 |
| P42858 | 3IO4 | HMDB0000641 | 4.133 |
| O15146 | 2IEP | HMDB0060348 | 4.131 |
| Q5S007 | 3D6T | HMDB0000094 | 4.131 |
| P21964 | 3HVH | HMDB0000097 | 4.13 |
| P01584 | 2NVH | HMDB0060348 | 4.129 |
| P01127 | 3MJG | HMDB0060348 | 4.128 |
| P00450 | 2J5W | HMDB0000094 | 4.124 |
| P35637 | 1G5G | HMDB0003681 | 4.122 |
| P05019 | 1H02 | HMDB0060348 | 4.121 |
| P20783 | 1BND | HMDB0060348 | 4.12 |
| P23560 | 1BND | HMDB0060348 | 4.12 |
| P06850 | 1MO1 | HMDB0060348 | 4.118 |
| P31749 | 2UZS | HMDB0000094 | 4.117 |
| P05231 | 1ALU | HMDB0060348 | 4.116 |
| P60484 | 1D5R | HMDB0003681 | 4.116 |
| Q9UBP0 | 3B9P | HMDB0003681 | 4.114 |
| Q7LG56 | 3HF1 | HMDB0060348 | 4.113 |
| O43612 | 1UV0 | HMDB0060348 | 4.112 |

| | | | |
|---|---|---|---|
| P01303 | 1R9N | HMDB0003681 | 4.111 |
| P78509 | 2DDU | HMDB0000641 | 4.11 |
| P35637 | 1G5G | HMDB0060348 | 4.109 |
| Q15303 | 2AHX | HMDB0003681 | 4.109 |
| O00555 | 3BXK | HMDB0060348 | 4.108 |
| Q99497 | 1PDW | HMDB0060348 | 4.106 |
| P01589 | 3IU3 | HMDB0060348 | 4.105 |
| P01241 | 1AXI | HMDB0000641 | 4.104 |
| P10912 | 1AXI | HMDB0000641 | 4.104 |
| Q16620 | 1HCF | HMDB0003681 | 4.104 |
| P51608 | 3C2I | HMDB0060348 | 4.102 |
| P42858 | 3IO4 | HMDB0060348 | 4.102 |
| Q16620 | 1HCF | HMDB0000094 | 4.101 |
| Q14203 | 2HL5 | HMDB0000641 | 4.1 |
| P06850 | 1MO1 | HMDB0003681 | 4.099 |
| Q13255 | 1EWT | HMDB0060348 | 4.098 |
| P78504 | 2VJ2 | HMDB0060348 | 4.098 |
| P31749 | 2UZS | HMDB0060348 | 4.097 |
| P05231 | 1ALU | HMDB0003681 | 4.096 |
| P01584 | 2NVH | HMDB0003681 | 4.096 |
| P51608 | 3C2I | HMDB0000641 | 4.096 |
| P60484 | 1D5R | HMDB0060348 | 4.095 |
| P10145 | 1QE6 | HMDB0060348 | 4.094 |
| P06850 | 1MO1 | HMDB0000641 | 4.093 |
| P35637 | 1G5G | HMDB0000094 | 4.091 |
| P68871 | 1BAB | HMDB0000097 | 4.089 |
| P01303 | 1R9N | HMDB0000094 | 4.086 |
| O43612 | 1UV0 | HMDB0003681 | 4.086 |
| P31749 | 2UZS | HMDB0003681 | 4.086 |
| Q9UBK2 | 1XB7 | HMDB0060348 | 4.084 |
| Q06787 | 2QND | HMDB0000094 | 4.084 |
| Q8NER1 | 2NYJ | HMDB0000094 | 4.083 |
| Q5S007 | 3D6T | HMDB0060348 | 4.083 |
| P16220 | 1DH3 | HMDB0060348 | 4.082 |
| P08833 | 1ZT3 | HMDB0003681 | 4.082 |
| P09619 | 1GQ5 | HMDB0060348 | 4.081 |
| P03372 | 1HCQ | HMDB0000883 | 4.081 |
| P10082 | 2ZA5 | HMDB0000097 | 4.081 |
| Q14203 | 2HL5 | HMDB0060348 | 4.079 |
| P26358 | 3EPZ | HMDB0000094 | 4.074 |
| P41159 | 3IIQ | HMDB0000094 | 4.074 |
| P78509 | 2DDU | HMDB0000094 | 4.073 |
| Q9NQC3 | 1OZN | HMDB0060348 | 4.071 |
| P01127 | 3MJG | HMDB0000641 | 4.07 |
| Q9Y243 | 2X18 | HMDB0003681 | 4.068 |
| Q13936 | 1T0J | HMDB0000094 | 4.067 |
| P22301 | 2ILK | HMDB0000094 | 4.067 |
| P08833 | 1ZT3 | HMDB0000094 | 4.066 |
| Q8NFD5 | 2EH9 | HMDB0060348 | 4.066 |
| P01911 | 2WBJ | HMDB0060348 | 4.066 |
| P51608 | 3C2I | HMDB0003681 | 4.065 |
| P01911 | 2WBJ | HMDB0003681 | 4.064 |
| Q15465 | 3M1N | HMDB0060348 | 4.063 |
| Q16620 | 1HCF | HMDB0060348 | 4.062 |
| Q14203 | 2HL5 | HMDB0003681 | 4.061 |
| P28329 | 2FY3 | HMDB0002329 | 4.06 |
| P00450 | 2J5W | HMDB0060348 | 4.058 |
| P41159 | 3IIQ | HMDB0060348 | 4.058 |
| P21397 | 2Z5Y | HMDB0000094 | 4.056 |
| P10082 | 2ZA5 | HMDB0000883 | 4.056 |
| P49917 | 3II6 | HMDB0060348 | 4.055 |
| P04062 | 2NT0 | HMDB0003681 | 4.054 |
| P21333 | 2WFN | HMDB0003681 | 4.053 |
| Q9Y2R2 | 2P6X | HMDB0000094 | 4.052 |
| P60568 | 1PY2 | HMDB0003681 | 4.052 |
| P01241 | 1AXI | HMDB0003681 | 4.05 |
| P10912 | 1AXI | HMDB0003681 | 4.05 |
| P78509 | 2DDU | HMDB0060348 | 4.05 |
| P06280 | 3HG3 | HMDB0000094 | 4.05 |

| | | | |
|---|---|---|---|
| P16220 | 1DH3 | HMDB0003681 | 4.048 |
| P01579 | 1EKU | HMDB0003681 | 4.047 |
| P05019 | 1H02 | HMDB0003681 | 4.044 |
| P01303 | 1R9N | HMDB0060348 | 4.04 |
| P20783 | 1BND | HMDB0003681 | 4.035 |
| P23560 | 1BND | HMDB0003681 | 4.035 |
| P01579 | 1EKU | HMDB0060348 | 4.035 |
| Q13255 | 1EWT | HMDB0000641 | 4.034 |
| O15146 | 2IEP | HMDB0003681 | 4.032 |
| Q9UBP0 | 3B9P | HMDB0000094 | 4.032 |
| Q13255 | 1EWT | HMDB0003681 | 4.03 |
| P11274 | 1K1F | HMDB0000094 | 4.03 |
| P00441 | 1HL5 | HMDB0060348 | 4.029 |
| P28482 | 1PME | HMDB0000097 | 4.027 |
| Q9UBP0 | 3B9P | HMDB0060348 | 4.025 |
| P03372 | 1HCQ | HMDB0000097 | 4.022 |
| P01920 | 1UVQ | HMDB0003681 | 4.02 |
| P21333 | 2WFN | HMDB0000094 | 4.02 |
| P27338 | 1OJA | HMDB0000094 | 4.019 |
| Q9Y243 | 2X18 | HMDB0060348 | 4.018 |
| P15289 | 2AIJ | HMDB0000641 | 4.013 |
| Q06787 | 2QND | HMDB0003681 | 4.011 |
| P21359 | 2D4Q | HMDB0000097 | 4.01 |
| P08833 | 1ZT3 | HMDB0060348 | 4.008 |
| P01375 | 2AZ5 | HMDB0000883 | 3.999 |
| Q8NER1 | 2NYJ | HMDB0060348 | 3.998 |
| P04049 | 3CU8 | HMDB0000883 | 3.997 |
| P22301 | 2ILK | HMDB0060348 | 3.991 |
| P15056 | 3II5 | HMDB0000883 | 3.99 |
| P42336 | 3HHM | HMDB0000883 | 3.978 |
| P04271 | 2H61 | HMDB0000097 | 3.966 |
| Q9Y2R2 | 2P6X | HMDB0060348 | 3.966 |
| P01920 | 1UVQ | HMDB0000641 | 3.935 |
| Q99259 | 2OKJ | HMDB0000094 | 3.93 |
| Q02750 | 3EQC | HMDB0000097 | 3.929 |
| P01138 | 1SG1 | HMDB0000159 | 3.927 |
| P55072 | 1Y8E | HMDB0000641 | 3.923 |
| P02787 | 1DAN | HMDB0000097 | 3.919 |
| P55072 | 1Y8E | HMDB0003681 | 3.915 |
| P35520 | 1M54 | HMDB0000097 | 3.9 |
| P16278 | 1BOB | HMDB0000883 | 3.892 |
| P42345 | 4FAP | HMDB0000883 | 3.887 |
| P22301 | 2ILK | HMDB0003681 | 3.884 |
| P42336 | 3HHM | HMDB0000097 | 3.868 |
| P51659 | 1IKT | HMDB0000097 | 3.861 |
| P01303 | 1SG1 | HMDB0001494 | 3.857 |
| P00441 | 1HL5 | HMDB0000883 | 3.846 |
| Q99497 | 1PDW | HMDB0000097 | 3.838 |
| P22301 | 2ILK | HMDB0000641 | 3.837 |
| Q8NFD5 | 2EH9 | HMDB0000883 | 3.819 |
| P23771 | 3DFV | HMDB0000883 | 3.819 |
| P62834 | 3KUC | HMDB0000883 | 3.804 |
| P00441 | 1HL5 | HMDB0000097 | 3.797 |
| P49917 | 3II6 | HMDB0000883 | 3.788 |
| P43354 | 1OVL | HMDB0000883 | 3.744 |
| P42345 | 4FAP | HMDB0000097 | 3.741 |
| P01375 | 2AZ5 | HMDB0000097 | 3.737 |
| P01112 | 1NVU | HMDB0000097 | 3.736 |
| P16435 | 3FJO | HMDB0000883 | 3.73 |
| P54098 | 1A1V | HMDB0000097 | 3.728 |
| P23771 | 3DFV | HMDB0000097 | 3.718 |
| P01112 | 1NVU | HMDB0000883 | 3.712 |
| P27986 | 1H9O | HMDB0000883 | 3.709 |
| O15146 | 2IEP | HMDB0000883 | 3.701 |
| P54098 | 1A1V | HMDB0000883 | 3.7 |
| Q13936 | 1T0J | HMDB0000641 | 3.678 |
| P04049 | 3CU8 | HMDB0000097 | 3.675 |
| P15056 | 3II5 | HMDB0000097 | 3.675 |
| Q16620 | 1HCF | HMDB0000883 | 3.671 |

| | | | |
|---|---|---|---|
| P04062 | 2NT0 | HMDB0000883 | 3.669 |
| P07949 | 2IVS | HMDB0000097 | 3.666 |
| P04637 | 1GZH | HMDB0000883 | 3.662 |
| Q9UBP0 | 3B9P | HMDB0000883 | 3.65 |
| P11362 | 1CVS | HMDB0000883 | 3.64 |
| P60568 | 1PY2 | HMDB0000883 | 3.637 |
| P16278 | 1BOB | HMDB0000097 | 3.633 |
| P27986 | 1H9O | HMDB0000097 | 3.624 |
| P21333 | 2WFN | HMDB0000883 | 3.617 |
| Q99497 | 1PDW | HMDB0000883 | 3.615 |
| Q7LG56 | 3HF1 | HMDB0000883 | 3.615 |
| P31749 | 2UZS | HMDB0000883 | 3.614 |
| P60484 | 1D5R | HMDB0000883 | 3.613 |
| Q8NER1 | 2NYJ | HMDB0000883 | 3.608 |
| P29475 | 1F20 | HMDB0000883 | 3.605 |
| P27487 | 1W1I | HMDB0000883 | 3.603 |
| O15146 | 2IEP | HMDB0000097 | 3.597 |
| P42858 | 3IO4 | HMDB0000883 | 3.597 |
| P01584 | 2NVH | HMDB0000883 | 3.593 |
| Q99700 | 3KTR | HMDB0000883 | 3.588 |
| P62834 | 3KUC | HMDB0000097 | 3.584 |
| P01303 | 1R9N | HMDB0000883 | 3.583 |
| P39905 | 2V5E | HMDB0000883 | 3.583 |
| P51608 | 3C2I | HMDB0000883 | 3.583 |
| P10145 | 1QE6 | HMDB0000883 | 3.582 |
| P11274 | 1K1F | HMDB0000097 | 3.581 |
| Q06787 | 2QND | HMDB0000883 | 3.579 |
| P26358 | 3EPZ | HMDB0000883 | 3.573 |
| Q9Y243 | 2X18 | HMDB0000883 | 3.572 |
| P01127 | 3MJG | HMDB0000883 | 3.572 |
| P35637 | 1G5G | HMDB0000883 | 3.571 |
| Q15465 | 3M1N | HMDB0000883 | 3.571 |
| P06280 | 3HG3 | HMDB0000883 | 3.57 |
| P16220 | 1DH3 | HMDB0000883 | 3.568 |
| P06850 | 1MO1 | HMDB0000883 | 3.566 |
| P41159 | 3IIQ | HMDB0000883 | 3.566 |
| P01241 | 1AXI | HMDB0000883 | 3.56 |
| P10912 | 1AXI | HMDB0000883 | 3.56 |
| P01911 | 2WBJ | HMDB0000883 | 3.557 |
| P05231 | 1ALU | HMDB0000883 | 3.555 |
| Q5S007 | 3D6T | HMDB0000883 | 3.555 |
| P09619 | 1GQ5 | HMDB0000883 | 3.549 |
| P01579 | 1EKU | HMDB0000883 | 3.547 |
| P05019 | 1H02 | HMDB0000883 | 3.547 |
| P43354 | 1OVL | HMDB0000097 | 3.547 |
| Q9Y2R2 | 2P6X | HMDB0000883 | 3.547 |
| Q9NQC3 | 1OZN | HMDB0000883 | 3.543 |
| P00450 | 2J5W | HMDB0000883 | 3.542 |
| P11274 | 1K1F | HMDB0000883 | 3.54 |
| P08833 | 1ZT3 | HMDB0000883 | 3.537 |
| P49917 | 3II6 | HMDB0000097 | 3.533 |
| P04062 | 2NT0 | HMDB0000097 | 3.531 |
| Q15303 | 2AHX | HMDB0000883 | 3.521 |
| P20783 | 1BND | HMDB0000883 | 3.518 |
| P23560 | 1BND | HMDB0000883 | 3.518 |
| O00555 | 3BXK | HMDB0000883 | 3.516 |
| Q14203 | 2HL5 | HMDB0000883 | 3.511 |
| P11362 | 1CVS | HMDB0000097 | 3.505 |
| P78509 | 2DDU | HMDB0000883 | 3.502 |
| Q9UBK2 | 1XB7 | HMDB0000883 | 3.5 |
| P16435 | 3FJO | HMDB0000097 | 3.487 |
| P01589 | 3IU3 | HMDB0000883 | 3.481 |
| Q9UBP0 | 3B9P | HMDB0000097 | 3.48 |
| P01589 | 3IU3 | HMDB0000097 | 3.476 |
| O43612 | 1UV0 | HMDB0000883 | 3.47 |
| P01584 | 2NVH | HMDB0000097 | 3.469 |
| P15289 | 2AIJ | HMDB0000883 | 3.462 |
| Q13255 | 1EWT | HMDB0000883 | 3.461 |
| Q16620 | 1HCF | HMDB0000097 | 3.461 |

| | | | |
|---|---|---|---|
| P10145 | 1QE6 | HMDB0000097 | 3.457 |
| P01920 | 1UVQ | HMDB0000883 | 3.449 |
| P21333 | 2WFN | HMDB0000097 | 3.444 |
| P01303 | 1R9N | HMDB0000097 | 3.44 |
| P35637 | 1G5G | HMDB0000097 | 3.437 |
| P04150 | 1P93 | HMDB0000159 | 3.434 |
| P04637 | 1GZH | HMDB0000097 | 3.433 |
| Q9Y243 | 2X18 | HMDB0000097 | 3.429 |
| P60568 | 1PY2 | HMDB0000097 | 3.425 |
| P29475 | 1F20 | HMDB0000097 | 3.424 |
| Q7LG56 | 3HF1 | HMDB0000097 | 3.422 |
| P60484 | 1D5R | HMDB0000097 | 3.416 |
| P06280 | 3HG3 | HMDB0000097 | 3.404 |
| P01579 | 1EKU | HMDB0000097 | 3.403 |
| P00450 | 2J5W | HMDB0000097 | 3.403 |
| P03372 | 1HCQ | HMDB0000159 | 3.399 |
| P39905 | 2V5E | HMDB0000097 | 3.399 |
| P08833 | 1ZT3 | HMDB0000097 | 3.397 |
| Q99700 | 3KTR | HMDB0000097 | 3.397 |
| P05019 | 1H02 | HMDB0000097 | 3.396 |
| P42858 | 3IO4 | HMDB0000097 | 3.394 |
| P78504 | 2VJ2 | HMDB0000883 | 3.389 |
| O00555 | 3BXK | HMDB0000097 | 3.386 |
| P41159 | 3IIQ | HMDB0000097 | 3.386 |
| Q15303 | 2AHX | HMDB0000097 | 3.385 |
| P22301 | 2ILK | HMDB0000097 | 3.385 |
| P06850 | 1MO1 | HMDB0000097 | 3.384 |
| P51608 | 3C2I | HMDB0000097 | 3.384 |
| P01241 | 1AXI | HMDB0000097 | 3.382 |
| P10912 | 1AXI | HMDB0000097 | 3.382 |
| Q13255 | 1EWT | HMDB0000097 | 3.382 |
| P20783 | 1BND | HMDB0000097 | 3.38 |
| P23560 | 1BND | HMDB0000097 | 3.38 |
| P01920 | 1UVQ | HMDB0000097 | 3.38 |
| Q06787 | 2QND | HMDB0000097 | 3.38 |
| Q13936 | 1T0J | HMDB0000097 | 3.379 |
| P15289 | 2AIJ | HMDB0000097 | 3.376 |
| P55072 | 1Y8E | HMDB0000883 | 3.374 |
| Q5S007 | 3D6T | HMDB0000097 | 3.374 |
| P16220 | 1DH3 | HMDB0000097 | 3.373 |
| P78509 | 2DDU | HMDB0000097 | 3.373 |
| Q14203 | 2HL5 | HMDB0000097 | 3.372 |
| P27487 | 1W1I | HMDB0000097 | 3.367 |
| Q15465 | 3M1N | HMDB0000097 | 3.367 |
| Q8NFD5 | 2EH9 | HMDB0000097 | 3.365 |
| Q9Y2R2 | 2P6X | HMDB0000097 | 3.352 |
| P26358 | 3EPZ | HMDB0000097 | 3.352 |
| P09619 | 1GQ5 | HMDB0000097 | 3.35 |
| Q9UBK2 | 1XB7 | HMDB0000097 | 3.348 |
| Q9NQC3 | 1OZN | HMDB0000097 | 3.346 |
| Q13936 | 1T0J | HMDB0000883 | 3.344 |
| P22301 | 2ILK | HMDB0000883 | 3.341 |
| P05231 | 1ALU | HMDB0000097 | 3.325 |
| O43612 | 1UV0 | HMDB0000097 | 3.323 |
| P55072 | 1Y8E | HMDB0000097 | 3.305 |
| P78504 | 2VJ2 | HMDB0000097 | 3.299 |
| P01116 | 1N4R | HMDB0000159 | 3.291 |
| P28329 | 2FY3 | HMDB0000159 | 3.243 |
| Q08499 | 1MKD | HMDB0000159 | 3.212 |
| P02768 | 1E7A | HMDB0000159 | 3.183 |
| P27338 | 1OJA | HMDB0010384 | 3.183 |
| P36551 | 2AEX | HMDB0000159 | 3.175 |
| P17612 | 3MVJ | HMDB0000159 | 3.145 |
| P01308 | 1EVR | HMDB0000159 | 3.128 |
| P05067 | 2FK3 | HMDB0000097 | 3.0963 |
| P10082 | 2ZA5 | HMDB0000159 | 3.086 |
| Q02750 | 3EQC | HMDB0000159 | 3.085 |
| Q9BYW2 | 3H6L | HMDB0000159 | 3.081 |
| P04278 | 1LHV | HMDB0000159 | 3.077 |

| | | | |
|---|---|---|---|
| P02787 | 1DAN | HMDB0000159 | 3.065 |
| P08069 | 3F5P | HMDB0000159 | 3.06 |
| P04271 | 2H61 | HMDB0000159 | 3.036 |
| P05067 | 2FK3 | HMDB0000159 | 3.034 |
| P01270 | 3C4M | HMDB0000159 | 3.031 |
| Q99259 | 2OKJ | HMDB0000159 | 3.028 |
| Q03431 | 3L2J | HMDB0000159 | 3.023 |
| P21964 | 3HVH | HMDB0000159 | 3.017 |
| P02741 | 1CGP | HMDB0000159 | 3.008 |
| Q30201 | 1DEA | HMDB0000159 | 2.996 |
| Q9Y617 | 3E77 | HMDB0000159 | 2.981 |
| Q15067 | 1W07 | HMDB0000159 | 2.959 |
| P28482 | 1PME | HMDB0000159 | 2.957 |
| Q06124 | 2SHP | HMDB0000159 | 2.955 |
| P21802 | 1DJS | HMDB0000159 | 2.946 |
| P14902 | 2D0T | HMDB0000159 | 2.93 |
| P68871 | 1BAB | HMDB0000159 | 2.927 |
| P27361 | 3FXW | HMDB0000159 | 2.903 |
| P51659 | 1IKT | HMDB0000159 | 2.898 |
| P42336 | 3HHM | HMDB0000159 | 2.87 |
| P21359 | 2D4Q | HMDB0000159 | 2.865 |
| P35520 | 1M54 | HMDB0000159 | 2.863 |
| P12821 | 1UZE | HMDB0000159 | 2.859 |
| P14416 | 2HLB | HMDB0000159 | 2.859 |
| P07949 | 2IVS | HMDB0000159 | 2.855 |
| P08397 | 3ECR | HMDB0000159 | 2.847 |
| P15056 | 3II5 | HMDB0000159 | 2.841 |
| P30793 | 1N3T | HMDB0000159 | 2.825 |
| P04049 | 3CU8 | HMDB0000159 | 2.801 |
| Q99497 | 1PDW | HMDB0000159 | 2.797 |
| P01375 | 2AZ5 | HMDB0000159 | 2.775 |
| P54098 | 1A1V | HMDB0000159 | 2.771 |
| P62834 | 3KUC | HMDB0000159 | 2.767 |
| P43354 | 1OVL | HMDB0000159 | 2.755 |
| P01112 | 1NVU | HMDB0000159 | 2.728 |
| P29475 | 1F20 | HMDB0000159 | 2.71 |
| P60484 | 1D5R | HMDB0000159 | 2.701 |
| P42345 | 4FAP | HMDB0000159 | 2.689 |
| Q9Y243 | 2X18 | HMDB0000159 | 2.671 |
| P23771 | 3DFV | HMDB0000159 | 2.669 |
| P06280 | 3HG3 | HMDB0000159 | 2.636 |
| P16435 | 3FJO | HMDB0000159 | 2.61 |
| P16278 | 1BOB | HMDB0000159 | 2.608 |
| P49917 | 3II6 | HMDB0000159 | 2.568 |
| Q8NFD5 | 2EH9 | HMDB0000159 | 2.522 |
| Q16620 | 1HCF | HMDB0000159 | 2.513 |
| P00450 | 2J5W | HMDB0000159 | 2.491 |
| P11362 | 1CVS | HMDB0000159 | 2.469 |
| P21333 | 2WFN | HMDB0000159 | 2.425 |
| Q9UBP0 | 3B9P | HMDB0000159 | 2.415 |
| P00441 | 1HL5 | HMDB0000159 | 2.396 |
| P01127 | 3MJG | HMDB0000159 | 2.395 |
| P55072 | 1Y8E | HMDB0000159 | 2.384 |
| O43612 | 1UV0 | HMDB0000159 | 2.379 |
| Q15303 | 2AHX | HMDB0000159 | 2.375 |
| Q9Y2R2 | 2P6X | HMDB0000159 | 2.374 |
| P22301 | 2ILK | HMDB0000159 | 2.37 |
| Q9UBK2 | 1XB7 | HMDB0000159 | 2.368 |
| P20783 | 1BND | HMDB0000159 | 2.367 |
| P23560 | 1BND | HMDB0000159 | 2.367 |
| Q13255 | 1EWT | HMDB0000159 | 2.366 |
| P01589 | 3IU3 | HMDB0000159 | 2.348 |
| P31749 | 2UZS | HMDB0000159 | 2.347 |
| P09619 | 1GQ5 | HMDB0000159 | 2.346 |
| Q06787 | 2QND | HMDB0000159 | 2.344 |
| Q99700 | 3KTR | HMDB0000159 | 2.344 |
| Q7LG56 | 3HF1 | HMDB0000159 | 2.343 |
| P78504 | 2VJ2 | HMDB0000159 | 2.342 |
| P27986 | 1H9O | HMDB0000159 | 2.341 |

| | | | |
|---|---|---|---|
| P04062 | 2NT0 | HMDB0000159 | 2.338 |
| P01241 | 1AXI | HMDB0000159 | 2.337 |
| P10912 | 1AXI | HMDB0000159 | 2.337 |
| P39905 | 2V5E | HMDB0000159 | 2.323 |
| P05019 | 1H02 | HMDB0000159 | 2.317 |
| P26358 | 3EPZ | HMDB0000159 | 2.317 |
| P10145 | 1QE6 | HMDB0000159 | 2.315 |
| O15146 | 2IEP | HMDB0000159 | 2.312 |
| Q15465 | 3M1N | HMDB0000159 | 2.309 |
| P01579 | 1EKU | HMDB0000159 | 2.303 |
| P04637 | 1GZH | HMDB0000159 | 2.296 |
| P01584 | 2NVH | HMDB0000159 | 2.295 |
| Q9NQC3 | 1OZN | HMDB0000159 | 2.292 |
| O00555 | 3BXK | HMDB0000159 | 2.287 |
| P60568 | 1PY2 | HMDB0000159 | 2.266 |
| Q14203 | 2HL5 | HMDB0000159 | 2.262 |
| P35637 | 1G5G | HMDB0000159 | 2.254 |
| P01911 | 2WBJ | HMDB0000159 | 2.254 |
| P15289 | 2AIJ | HMDB0000159 | 2.253 |
| P01920 | 1UVQ | HMDB0000159 | 2.251 |
| Q13936 | 1T0J | HMDB0000159 | 2.25 |
| P08833 | 1ZT3 | HMDB0000159 | 2.249 |
| P42858 | 3IO4 | HMDB0000159 | 2.247 |
| P06850 | 1MO1 | HMDB0000159 | 2.245 |
| Q5S007 | 3D6T | HMDB0000159 | 2.245 |
| P05231 | 1ALU | HMDB0000159 | 2.244 |
| P27487 | 1W1I | HMDB0000159 | 2.242 |
| P51608 | 3C2I | HMDB0000159 | 2.241 |
| Q8NER1 | 2NYJ | HMDB0000159 | 2.233 |
| P11274 | 1K1F | HMDB0000159 | 2.225 |
| P16220 | 1DH3 | HMDB0000159 | 2.207 |
| P01303 | 1R9N | HMDB0000159 | 2.18 |
| P41159 | 3IIQ | HMDB0000159 | 2.166 |
| P78509 | 2DDU | HMDB0000159 | 2.138 |
